# AI-Based Measurement Tools for Intrinsic Capacity in Older Adults: Scoping Review

**DOI:** 10.2196/90411

**Published:** 2026-10-05

**Authors:** Chengji Yu, Ping Lu, Ying Zhou, Juan Zhao, Xiaodie Yang, Dayu Tang, Liying Ying

**Affiliations:** 1 Department of Nursing, The Second Affiliated Hospital of Zhejiang University School of Medicine, No. 88 Jiefang Road, Shangcheng District, Hangzhou, Zhejiang, 310009, China, 86 13706501420; 2 School of Nursing, Zhejiang University School of Medicine, Hangzhou, Zhejiang, China

**Keywords:** intrinsic capacity, IC, artificial intelligence, AI, digital biomarker, older adults, measurement

## Abstract

**Background:**

Intrinsic capacity (IC) has become a central concept in healthy aging because it emphasizes functional ability across the aging trajectory rather than disease alone. However, current IC assessment primarily relies on episodic clinical evaluations, which are insufficient for continuous monitoring and early identification of functional decline. Recent advances in AI and digital health technologies have created new opportunities for objective, continuous, and real-world assessment of IC. However, existing evidence remains fragmented across AI-enabled devices, digital biomarkers (DBs), AI techniques, and IC domains.

**Objective:**

This scoping review aimed to systematically synthesize the current evidence on AI-based measurement tools for IC and to characterize the landscape of AI-enabled IC assessment using a 3D analytical framework integrating AI-enabled digital devices and systems, DBs, and AI techniques.

**Methods:**

A comprehensive search of PubMed, Embase, CINAHL, PsycINFO, the Cochrane Library, SinoMed, and China National Knowledge Infrastructure (CNKI) was conducted from database inception to July 2025 and updated on May 31, 2026, in accordance with the PRISMA-ScR (Preferred Reporting Items for Systematic Reviews and Meta-Analyses extension for Scoping Reviews) guideline. Studies investigating AI-based measurement tools applicable to one or more IC domains were included.

**Results:**

A total of 161 studies met the inclusion criteria. Research on AI-based measurement tools for IC has expanded rapidly since 2016, with studies conducted in 28 countries, predominantly the United States and China. Most studies focused on a single IC domain, with cognition accounting for the largest proportion. Eleven categories of AI-enabled digital devices and systems were identified, among which multimodal data acquisition devices, computer vision (CV) systems, and AI-driven health platforms were the most frequently reported. Twenty-one types of DBs were extracted and classified into 3 major categories, with gait parameters, digital task performance, physical activity features, speech and language features, and facial features representing the most commonly used biomarkers. Machine learning and deep learning were the predominant AI techniques, while CV and natural language processing played central roles in multimodal data interpretation. The distribution and maturity of evidence varied substantially across domains, with cognition and locomotor capacity representing the most developed areas, whereas vitality, hearing, and multidomain IC assessment remained comparatively underrepresented.

**Conclusions:**

This scoping review provides a 3D synthesis of AI-enabled digital devices and systems, DBs, and AI techniques across the 6 World Health Organization (WHO)–defined domains of IC. Unlike previous technology-, disease-, or domain-specific reviews, it compares evidence across the broader IC framework, identifying more developed areas, key evidence gaps, and priorities for standardization, external validation, and multidomain assessment. AI-based measurement tools may complement conventional assessment in community, primary care, and home settings, although their clinical translation will require robust validation, integration into care pathways, and implementation approaches that address the needs of older adults.

## Introduction

Population aging has become one of the most significant global demographic trends, shifting the focus of health care for older adults from disease-centered management toward the maintenance of functional ability and healthy aging [1]. To support this transition, the World Health Organization (WHO) introduced the concept of intrinsic capacity (IC), defined as the composite of an individual’s physical and mental capacities [2]. As a multidimensional construct, IC reflects the functional resources available to an individual throughout the aging process [1-3]. Declining IC has been associated with an increased risk of frailty, disability, care dependency, and mortality, making it an important target for early identification and preventive intervention [3,4]. Because IC is dynamic rather than static, repeated assessment is needed to characterize trajectories of functional change, detect early deterioration, and support timely interventions before substantial functional decline occurs. Monitoring changes in IC over time is therefore increasingly recognized as an important component of healthy aging strategies and person-centered care [2,5].

Current approaches to IC assessment primarily rely on questionnaires, standardized performance tests, and clinical or laboratory-based assessments [6]. Although these methods remain central to the evaluation of functional capacity in older adults, some require in-person administration, trained assessors, and considerable clinical time and resources, which may limit the feasibility of frequent reassessment in routine practice [3,5,7]. Conventional assessments are also commonly administered at discrete time points and may therefore provide limited information on changes in IC over time or variation across everyday settings [6]. Assessments conducted in clinical settings may not fully reflect an individual’s habitual functioning in daily life [5,6]. These limitations constrain the scalability of current IC assessment and its use for longitudinal monitoring. Complementary approaches are therefore needed to support more frequent evaluation across the aging trajectory [2,4].

Recent advances in AI and digital health technologies have created new opportunities for objective and longitudinal assessment of IC [8,9]. According to the WHO integrated care for older people (ICOPE) framework, IC comprises 6 interrelated domains, including cognition, locomotor capacity, vitality, psychological capacity, vision, and hearing. Across these domains, AI-enabled digital devices and systems can capture multimodal functional, behavioral, and physiological data during structured assessments and in real-world settings [10-14]. Features derived from these data, such as movement and activity patterns, digital task performance, and speech and language characteristics, may serve as digital biomarkers (DBs) of functional capacity [15]. AI techniques support the extraction, integration, and interpretation of relevant features, enabling the identification, classification, or prediction of IC-related impairment and decline [16]. This measurement pathway may complement conventional assessments by enabling more frequent evaluation of functional capacity in settings that more closely reflect older adults’ daily lives.

Previous reviews have addressed related aspects of IC measurement and digital health in older adults. Reviews of IC measurement have mainly examined the characteristics and methodological quality of available assessment instruments [6], whereas reviews of AI and digital health have focused on broader applications of AI in health care for older adults [17], DBs for IC monitoring within the ICOPE framework [18], digital health approaches for dementia and cognitive impairment [19], or AI-based fall risk assessment [20]. These reviews have provided important insights into different aspects of technology-enabled assessment in older adults. However, they have generally addressed AI methods, DBs, clinical conditions, and functional domains as separate areas of inquiry. This approach provides limited insight into how AI-enabled digital devices and systems, the DBs derived from them, and the AI techniques used for analysis are connected across the full range of IC domains. Cross-domain differences in the distribution and maturity of evidence, together with the potential of current approaches for multidomain assessment and application in primary care, community, and home-based settings, have also received limited attention. These limitations highlight the need for a broader synthesis of AI-based measurement tools across the full range of IC domains, with particular attention to cross-domain evidence gaps and priorities for further validation and clinical application.

To address these gaps, this scoping review systematically maps and synthesizes the evidence on AI-based measurement tools for IC across the 6 WHO-defined domains. Using a 3D analytical framework, we examine the AI-enabled digital devices and systems used for data acquisition, the DBs derived from these data, and the AI techniques applied for data analysis and interpretation. We further examine how these components have been applied across IC domains, compare the distribution and maturity of the available evidence, and identify areas where evidence remains limited. The findings are intended to inform future research, support multidomain assessment, and facilitate the development of clinically applicable approaches for primary care, community, and home-based settings.

## Methods

### Overview

A scoping review was conducted following the methodological framework proposed by Arksey and O’Malley [21] and refined by Levac et al [22]. This approach provides a systematic and flexible framework for reviewing rapidly evolving and methodologically diverse evidence, such as AI-based tools for measuring IC. The review was reported in accordance with the PRISMA-ScR (Preferred Reporting Items for Systematic Reviews and Meta-Analyses extension for Scoping Reviews) checklist [23].

### Identifying the Research Question

This scoping review was guided by the population-concept-context (PCC) framework [19]. The overall aim was to clarify how AI-based measurement tools have been used to assess IC in older adults and to summarize the corresponding AI-enabled digital devices, DBs, and AI techniques. The population was older adults aged 60 years or older. The concept was AI-based measurement tools for IC assessment, including device and system types, DBs, and AI techniques. The context was primary care and routine care settings for older adults. IC was considered across 6 domains, including cognition, locomotor capacity, vitality, vision, hearing, and psychological capacity.

Based on the PCC framework, this review addressed the following research questions:

- In primary care, including community and home-based settings, which types of AI-based measurement tools or AI-enabled digital devices are used to assess IC in older adults?
- What DBs have been used to identify declines or impairments in the 6 domains of IC among older adults?
- What categories of AI techniques have been used to assess, classify, or predict IC status or decline among older adults?

### Protocol and Registration

The study protocol was registered in the Open Science Framework Registry [24]. The completed PRISMA-ScR checklist is provided in Checklist 1.

### Eligibility Criteria

Studies were selected according to predefined inclusion and exclusion criteria based on the PCC framework. Eligible studies included older adults aged 60 years or older. Studies were included if the measurement tools assessed IC or at least one of the 6 IC domains: cognition, locomotor capacity, vitality, vision, hearing, and psychological capacity. The tools also had to use digital devices or systems to collect DBs and apply AI techniques, such as machine learning (ML), deep learning (DL), natural language processing (NLP), or computer vision (CV). Eligible settings were defined with reference to the WHO ICOPE handbook as settings that support first-contact, accessible, continuous, comprehensive, and coordinated care delivered close to where older people live. These settings included primary care facilities, community health centers, outpatient clinics, home-based assessment, telehealth, mobile or outreach services, and other routine care settings for older adults. Peer-reviewed studies published in English or Chinese were included. Eligible study designs included experimental, quasi-experimental, observational, and mixed methods studies.

In this review, IC decline was defined as measurable impairment, deterioration, or an increased risk of decline in one or more of the 6 IC domains. Disease-based outcomes were included when they reflected decline within a specific IC domain. For example, mild cognitive impairment and dementia were mapped to cognition; gait impairment, falls, frailty, and limited mobility were mapped to locomotor capacity; malnutrition, undernutrition, sarcopenia, and low grip strength were mapped to vitality; visual impairment and eye disease screening outcomes were mapped to vision; hearing loss and hearing-threshold decline were mapped to hearing; and depression or depressive symptoms were mapped to psychological capacity.

Studies were excluded if the tools could not distinguish IC decline from healthy functioning, identify impairment in at least one IC domain, or assess the degree or risk of decline. Studies were also excluded if the assessment required highly specialized equipment that is not usually available in routine primary care or community-based assessment, such as magnetic resonance imaging (MRI), positron emission tomography–computed tomography (PET-CT), electroencephalography (EEG), or optical coherence tomography (OCT). Studies involving invasive procedures, such as venipuncture for blood tests or mydriatic eye drops, were also excluded. The full inclusion and exclusion criteria are presented in Table 1.

**Table 1.** Inclusion and exclusion criteria.

PCC^a^	Eligibility criteria and variables	Rationale
Inclusion criteria		
Population	Older adults aged ≥60 years	Focuses on the population targeted by IC^b^ assessment in aging care
Concept	AI-based measurement tools assessing IC or at least one of the 6 IC domains: cognition, locomotor capacity, vitality, vision, hearing, and psychological capacity	Ensures that included studies address IC or a clearly defined IC domain
Concept	Digital devices or systems used to collect digital biomarkers	Ensures that IC assessment is based on digitally captured, measurable indicators
Concept	AI techniques applied to analyze or interpret the collected data, such as ML^c^, DL^d^, NLP^e^, or CV^f^	Ensures that the tool includes an AI-based analytic component rather than digital data collection alone
Context	Settings consistent with primary care or routine care for older adults, including primary care facilities, community health centers, outpatient clinics, home-based assessment, telehealth, mobile, or outreach services	Defines primary care in an internationally applicable way and avoids restriction to any country-specific health system
Others	Peer-reviewed studies published in English or Chinese	Ensures that included evidence has undergone peer review and can be reliably screened by the review team
Others	Experimental, quasi-experimental, observational, or mixed methods studies	Includes empirical studies that report original data on AI-based measurement tools for IC
Exclusion criteria		
Concept	Tools unable to distinguish IC decline from healthy status, identify impairment in at least one IC domain, or assess the degree or risk of decline	Excludes tools that do not provide an interpretable assessment of IC status or decline
Context	Tools requiring highly specialized infrastructure, such as EEG^g^, MRI^h^, PET-CT^i^, or OCT^j^	Excludes approaches that usually require specialist equipment, trained personnel, or referral to specialized services beyond routine primary care assessment
Others	Invasive procedures, such as venipuncture for blood tests or mydriatic eye drops	Ensures consistency with the review focus on noninvasive digital and AI-based assessment

a PCC: population-concept-context framework.

b IC: intrinsic capacity.

c ML: machine learning.

d DL: deep learning.

e NLP: natural language processing.

f CV: computer vision.

g EEG: electroencephalography.

h MRI: magnetic resonance imaging.

i PET-CT: positron emission tomography–computed tomography.

j OCT: optical coherence tomography.

### Information Sources

A comprehensive literature search was conducted in 7 electronic databases: PubMed, Embase, CINAHL, APA PsycINFO, the Cochrane Library, SinoMed, and China National Knowledge Infrastructure (CNKI). Google Scholar was searched for gray literature, and the reference lists of eligible studies and relevant reviews were manually screened to identify additional records.

### Search Strategy

The search strategy was reported with reference to PRISMA-S [25]. Search strategies were developed separately for each database by combining controlled vocabulary and free-text terms. The main search terms covered older adults, IC, AI, digital devices, and DBs. Because the assessment of single or multiple IC domains had been studied before and after the WHO introduced the concept of IC, terms for cognition, locomotor capacity, vitality, vision, hearing, and psychological capacity were also included.

The original search included records published up to July 2025. Before resubmission, the same search strategies were run again in all databases on May 31, 2026, to identify newly published studies. The full search strategies for each database, including database names, search dates, search terms, limits, and numbers of records retrieved, are provided in Multimedia Appendix 1.

### Selection of Sources of Evidence

All retrieved records were imported into EndNote X9 (Clarivate) for automatic and manual deduplication. After deduplication, 2 authors (CY and PL) independently screened the titles and abstracts against the eligibility criteria. Records considered potentially eligible by either reviewer were retained for full-text assessment. The same 2 authors (CY and PL) then independently reviewed the full texts and determined final inclusion according to the predefined inclusion and exclusion criteria. Reasons for exclusion were recorded during full-text assessment. Any disagreements were resolved through discussion, and unresolved disagreements were resolved through adjudication by a third author (LY). The same selection process was applied to records identified in the updated search.

### Data Charting Process

Data from the included studies were charted using a predesigned standardized extraction form. The form was organized by IC domain and covered study characteristics, AI-based measurement tool characteristics, DBs, and AI techniques. Before formal data charting, the form was tested on a small sample of included studies and refined by the review team. Two authors (CY and PL) independently charted the data, and any uncertainties were resolved through discussion among the review team. Study authors were not contacted for additional or missing data.

### Data Items

For each included study, we extracted information on the publication year and source, sample size, participant age, IC-related condition, IC domains assessed, type of AI-based measurement tool, DBs, and AI techniques applied for data analysis and interpretation. IC-related outcomes were mapped to the 6 IC domains according to the operational definition described in the eligibility criteria.

### Synthesis of Results

Descriptive analyses were used to summarize publication characteristics, participant characteristics, IC domains, and study settings. The identified AI-based measurement tools, DBs, and AI techniques were then grouped and summarized according to their reported functions, measurement targets, and IC domains. The findings were presented in tables and figures, with detailed descriptions provided in the Results section.

## Results

### Literature Search

The study identification process is illustrated in Figure 1. In the original search, 13,235 records were identified. After removal of 2516 duplicates, 10,719 records underwent title and abstract screening, and 10,372 were excluded. Most excluded records did not assess IC or its domains, did not involve AI technologies or AI-enabled digital devices, focused on populations other than older adults, or did not meet the predefined eligibility criteria. Of the 347 studies assessed for full-text eligibility, 151 studies met the inclusion criteria. Citation searching identified 25 additional records, of which 6 studies were included. Thus, 157 studies were included in the original review.

An updated search conducted on May 31, 2026, identified 6781 records. After duplicate removal, 5492 records were screened, 5469 were excluded, and 23 full-text studies were assessed for eligibility. Four additional studies met the inclusion criteria. Consequently, 161 studies [26-186] were included in the final review.

**Figure 1.**
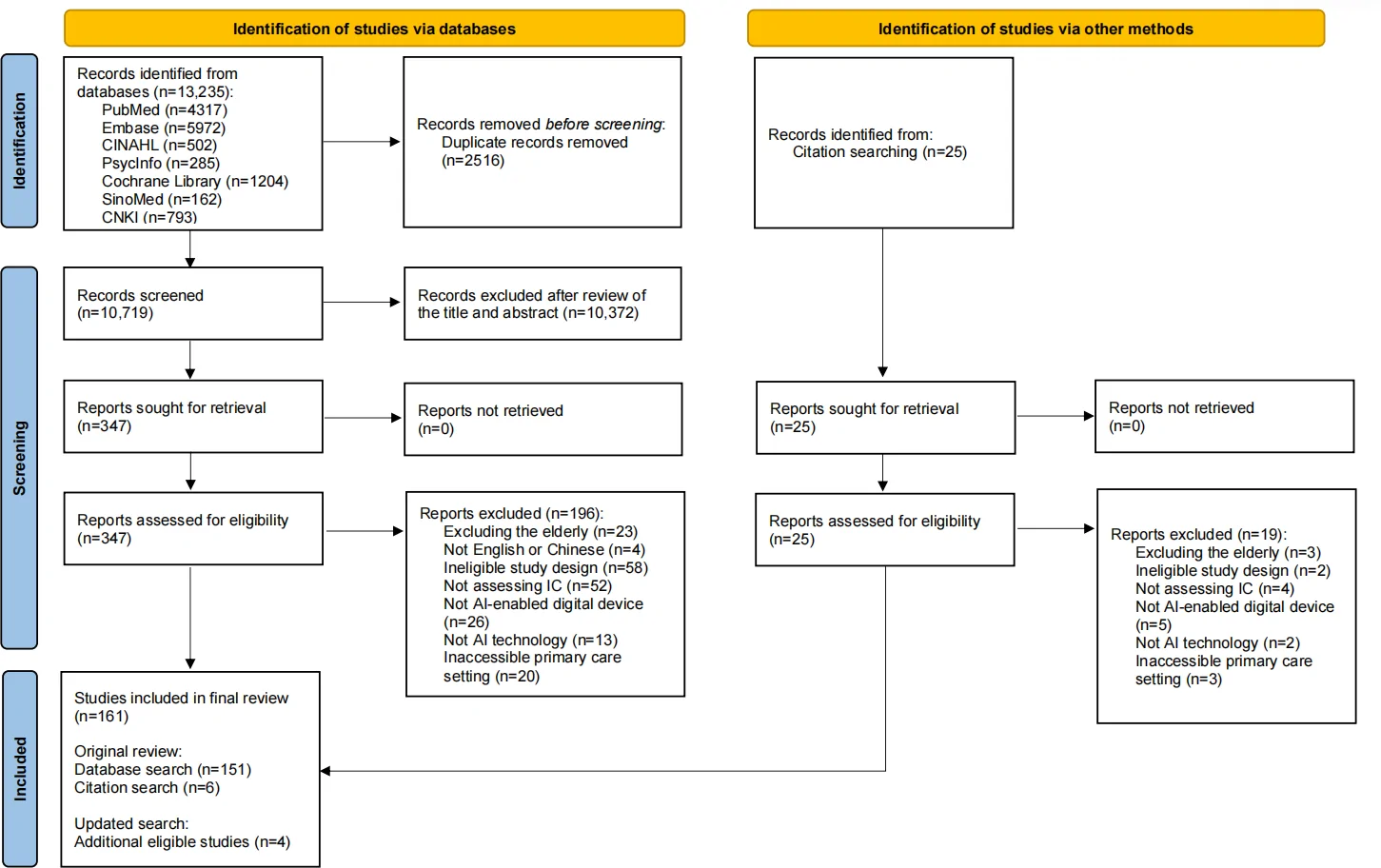
PRISMA (Preferred Reporting Items for Systematic Reviews and Meta-Analyses) flowchart of the selection process.

### Characteristics of the Included Studies

Among the 161 included studies, 4 were published in Chinese (2.48%). The number of studies increased markedly after 2016, with most studies published between 2021 and 2026 (71.43%), indicating a rapidly growing interest in AI-based measurement of IC among older adults (Table 2, Multimedia Appendix 2).

**Table 2.** Characteristics of the included studies.

Item	Number of studies (N=161)
Years of publication, n (%)	
2008‐2010	3 (1.86)
2011‐2015	4 (2.48)
2016‐2020	39 (24.22)
2021‐2026	115 (71.43)
The domain of IC^a^	
Cognition	70 (43.48)
Locomotor capacity	21 (13.04)
Vitality	9 (5.59)
Vision	16 (9.94)
Hearing	8 (4.97)
Psychological capacity	28 (17.39)
Multiple IC domains	9 (5.59)

a IC: intrinsic capacity.

The studies were conducted across 28 countries. The United States (n=33, 20.50%) [30,40,47,48,51,63,64,66,67,78,80,83,88,97,101,106,118,122,126,128-132,136,138,139,144,158,163,164,176,179] and China contributed the largest number of studies (n=33, 20.50% ) [32,45,46,50,58,59,61,62,68,70,72,76,84,93,102,103,110,114,117,133,134,140,148,150,152,154,155,159,160,173,184-186], followed by Korea (n=20, 12.42%) [27-29,31,56,77,82,96,99,108,109,111,112,153,165,170-172,177,181] (Figure 2). Most studies adopted observational designs and were conducted in settings relevant to primary care, including clinical or laboratory testing, community, home, and online environments.

**Figure 2.**
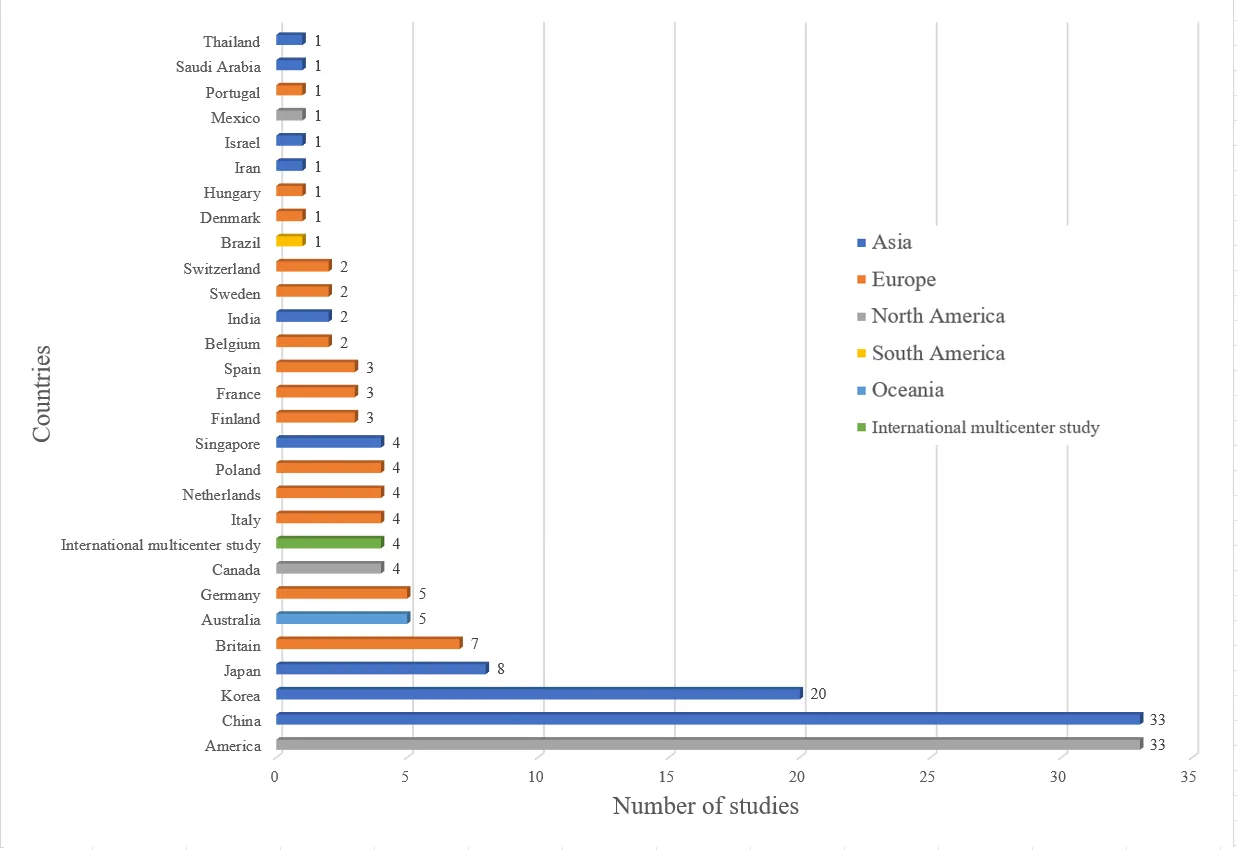
Number of studies by country.

All included studies involved older adults (aged ≥60 years), with sample sizes ranging from 4 participants in a feasibility study [26] to 1,904,927 participants. Participants included older adults with intact IC as well as those with impairment in one or more IC domains. According to the WHO ICOPE framework, IC outcomes were mapped to 6 domains, including cognition, locomotor capacity, vitality, vision, hearing, and psychological capacity. Cognition was the most frequently assessed domain (n=70, 43.48%), followed by psychological capacity (n=28, 17.39%), locomotor capacity (n=21, 13.04%), vision (n=16, 9.94%), vitality (n=9, 5.59%), and hearing (n=8, 4.97%). Nine studies assessed multiple IC domains simultaneously (Figure 3). Detailed characteristics of the included studies are provided in Multimedia Appendix 3.

**Figure 3.**
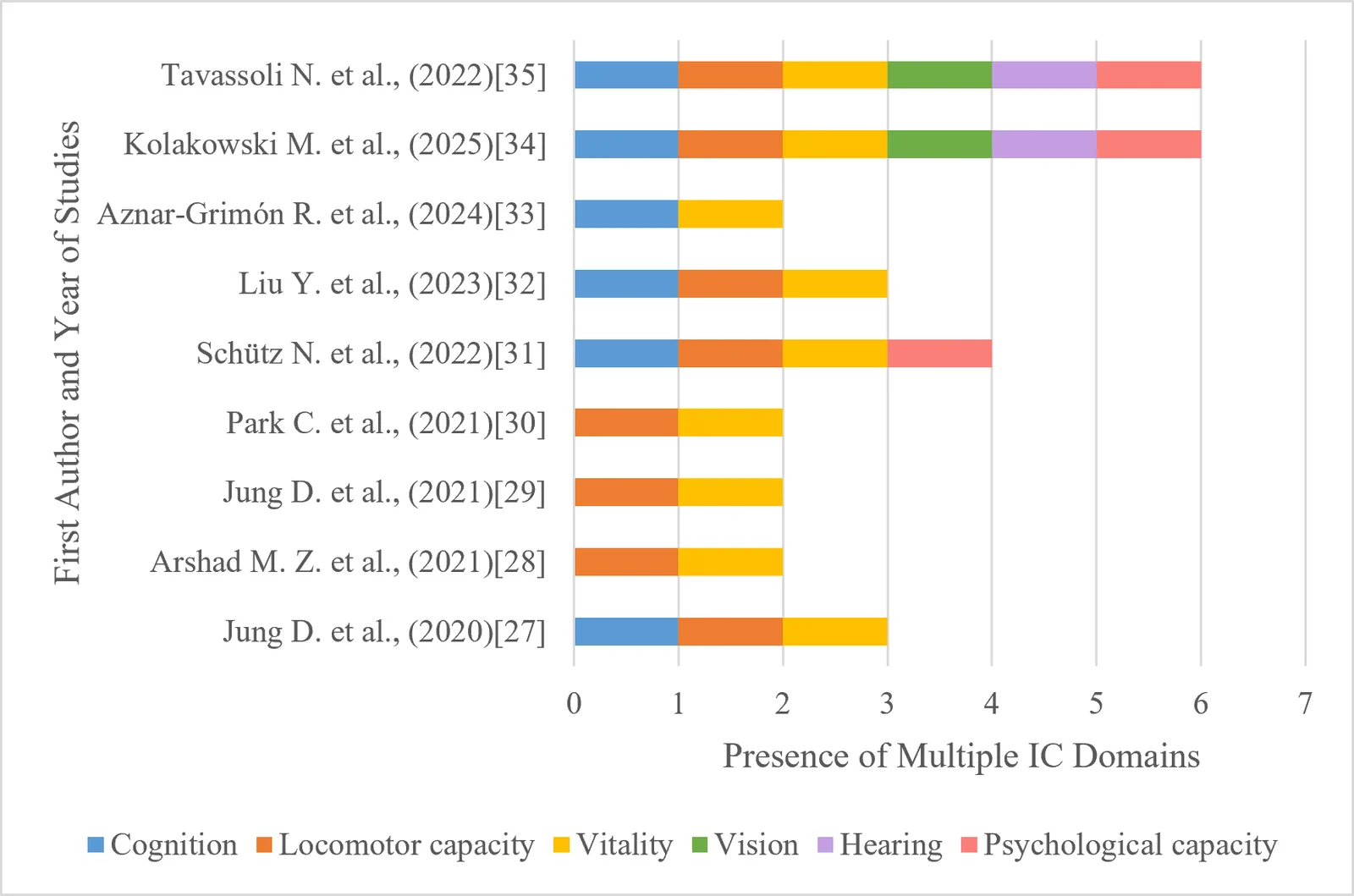
Studies on the measurement of multiple intrinsic capacity (IC) domains [27-35]. IC: intrinsic capacity.

### AI-Based Measurement Tools for IC

In this review, AI-based IC measurement tools were conceptualized as integrated assessment systems comprising 3 interrelated layers, including AI-enabled digital devices and systems, DBs, and AI techniques. AI-enabled digital devices and systems collect IC-related data, DBs represent measurable features derived from the collected data, and AI techniques process, classify, or interpret these features to support the assessment of one or more IC domains. The resulting IC assessments were mapped to the 6 WHO ICOPE domains: cognition, locomotor capacity, vitality, vision, hearing, and psychological capacity. This conceptual framework is illustrated in Figure 4.

**Figure 4.**
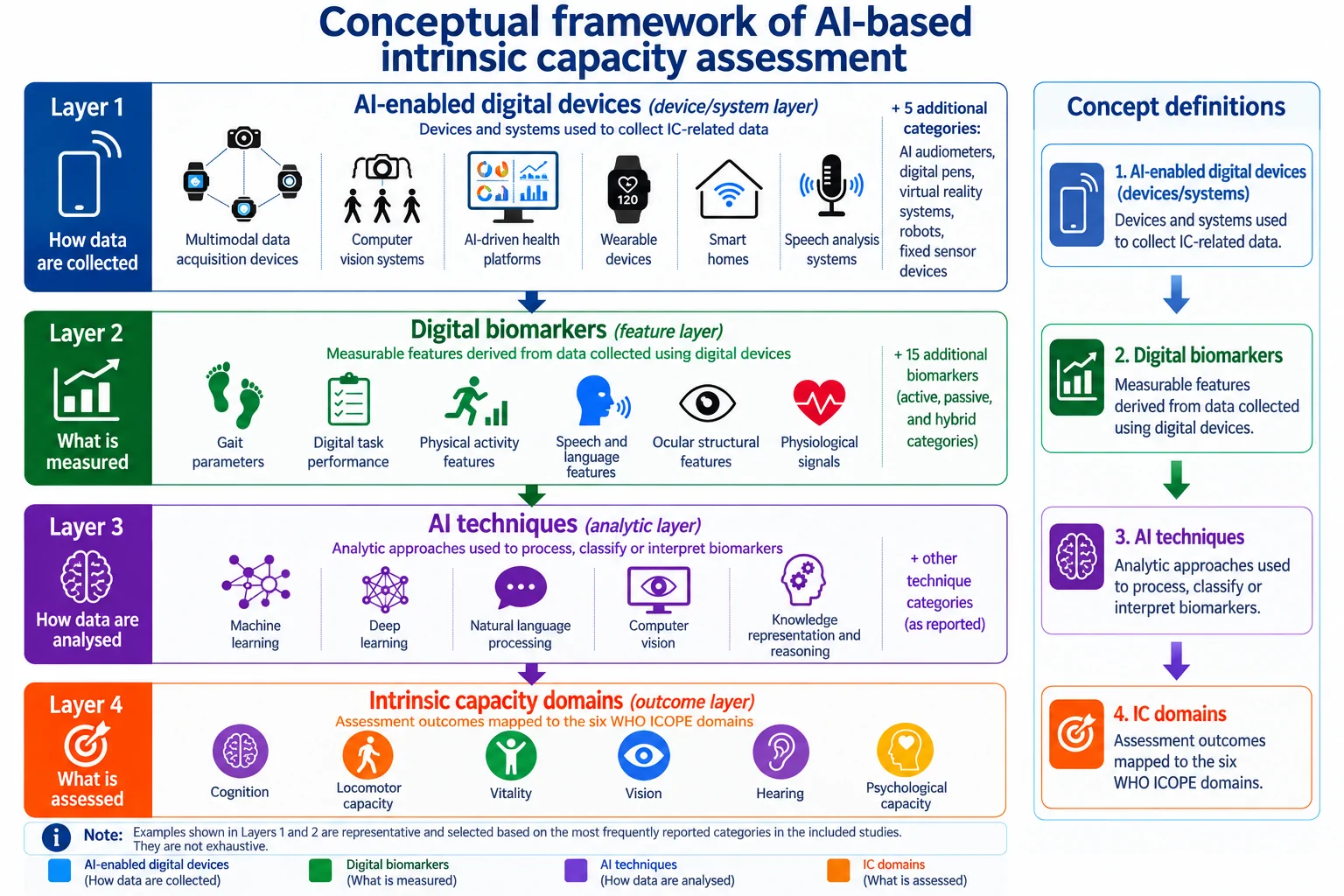
Conceptual framework of AI-based intrinsic capacity assessment. IC: intrinsic capacity; ICOPE: integrated care for older people; WHO: World Health Organization.

The following sections therefore summarize the findings according to these 3 layers. First, we describe the types and applications of AI-enabled digital devices and systems used for IC-related data collection. Second, we summarize the DBs derived from these data. Third, we report the AI techniques used to process, classify, or interpret these biomarkers for IC assessment. Detailed study-level information on these 3 components is provided in Multimedia Appendix 4. A glossary of key concepts and technical terms used in this review is provided in Multimedia Appendix 5.

### AI-Enabled Digital Devices and Systems

The distribution of IC domains across AI-enabled digital devices and systems is shown in Figure 5. Eleven types of AI-enabled digital devices and systems were identified. Multimodal data acquisition devices (MDADs) were the most common (21.12%), followed by CV systems (18.63%), AI-driven health platforms (17.39%), wearable devices (16.15%), smart homes (6.83%), speech analysis systems (6.83%), AI audiometers (3.73%), digital pens (3.11%), virtual reality (VR) systems (2.48%), robots (1.86%), and fixed sensor devices (1.86%).

**Figure 5.**
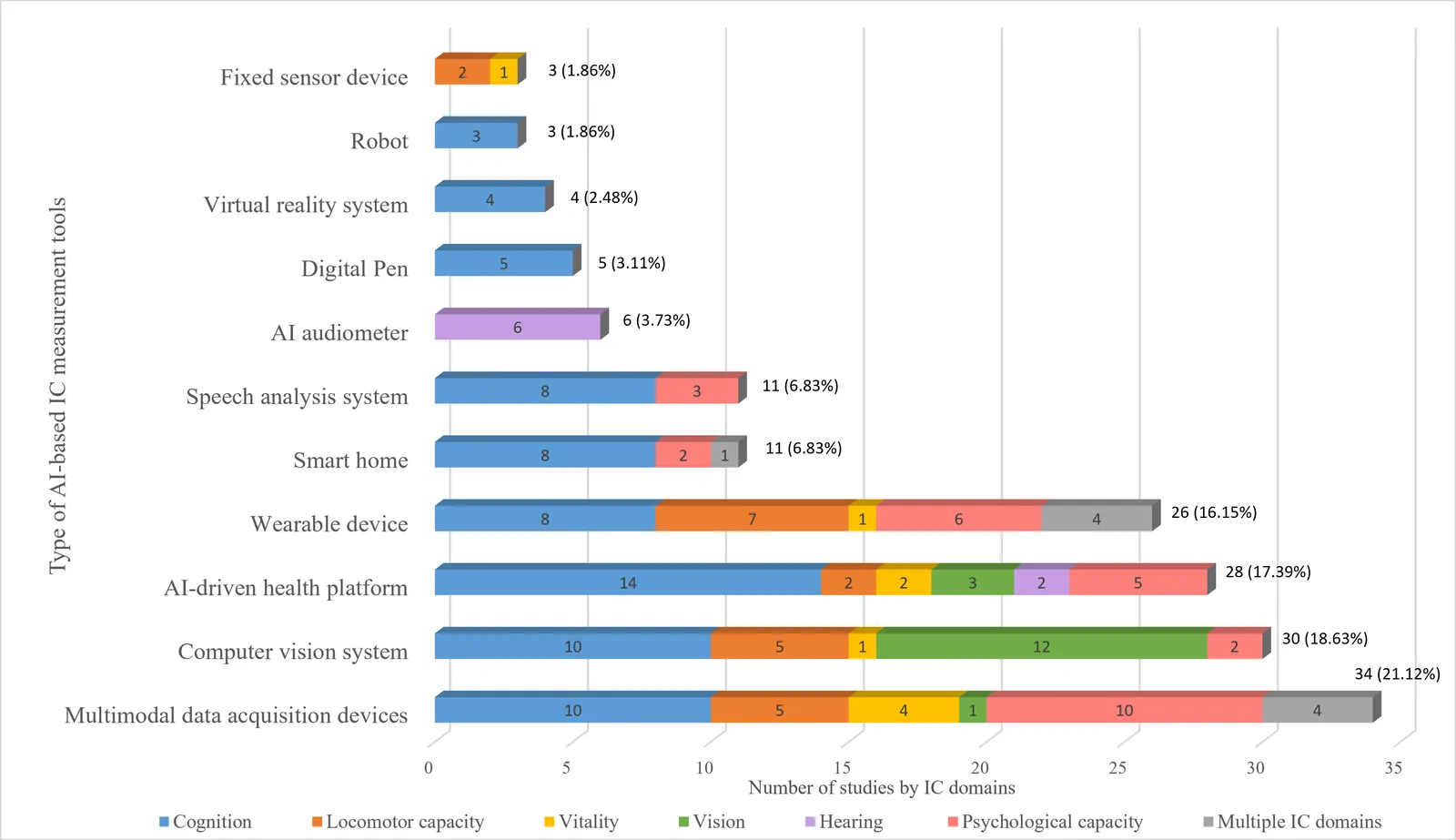
Distribution of intrinsic capacity (IC) domains across AI-enabled digital devices and systems. IC: intrinsic capacity.

To further synthesize the distribution of evidence across IC domains and device categories, an evidence gap map was constructed (Figure 6). The map shows that evidence was concentrated in cognition, where multiple device categories were represented, including AI-driven health platforms, CV systems, MDADs, wearable devices, smart homes, and speech analysis systems. Locomotor capacity was mainly assessed using wearable devices, CV systems, and MDADs. Vision and hearing showed more device-specific patterns, with evidence mainly concentrated in CV systems and AI audiometers, respectively. In contrast, vitality and multidimensional IC assessment were relatively underrepresented, with sparse evidence across most device categories. Several device-domain combinations showed no available evidence, indicating clear gaps in the current literature.

The usage proportions of different AI-enabled digital devices and systems across the 6 IC domains and multidimensional IC are illustrated in Figure 7. In the cognition domain, 9 of the 11 categories were involved. Notably, robots, digital pens, and VR systems were specifically used for cognitive capacity assessment. In the locomotor capacity domain, wearable devices were the most commonly used tools. In the vitality and psychological capacity domains, MDADs were the most frequently used, such as combinations of a 3D camera and a bioelectrical impedance analysis (BIA) scale for vitality assessment and smartwatches together with ecological momentary assessment (EMA) apps for psychological capacity assessment. CV systems were the predominant tools for vision assessment, while AI audiometers were most commonly used for hearing assessment, followed by AI-driven health platforms. For multidimensional IC, MDADs and wearable devices were the most frequently used categories.

**Figure 6.**
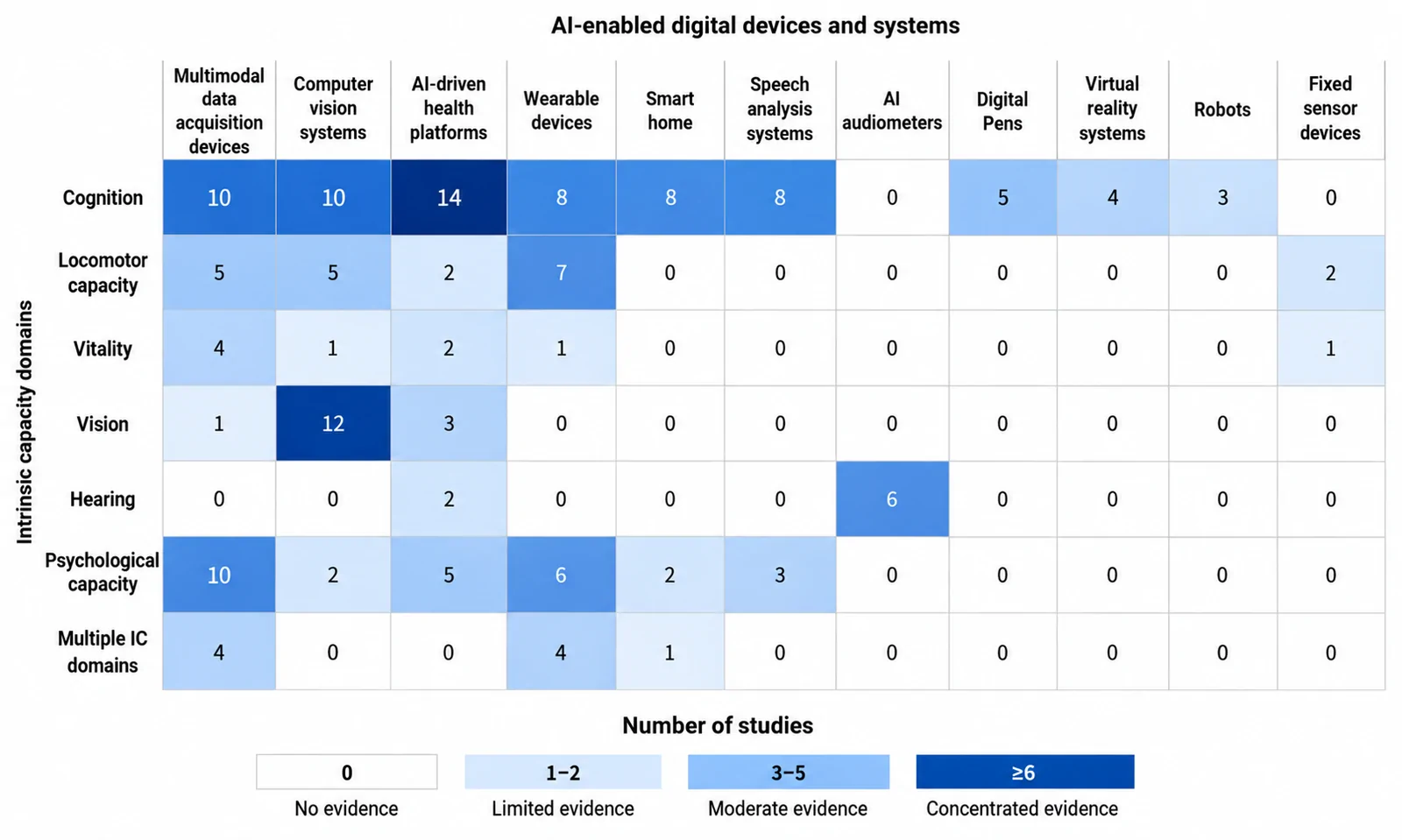
Evidence gap map of AI-enabled digital devices and systems. Darker colors indicate a larger number of studies, whereas white indicates no available evidence. IC: intrinsic capacity.

**Figure 7.**
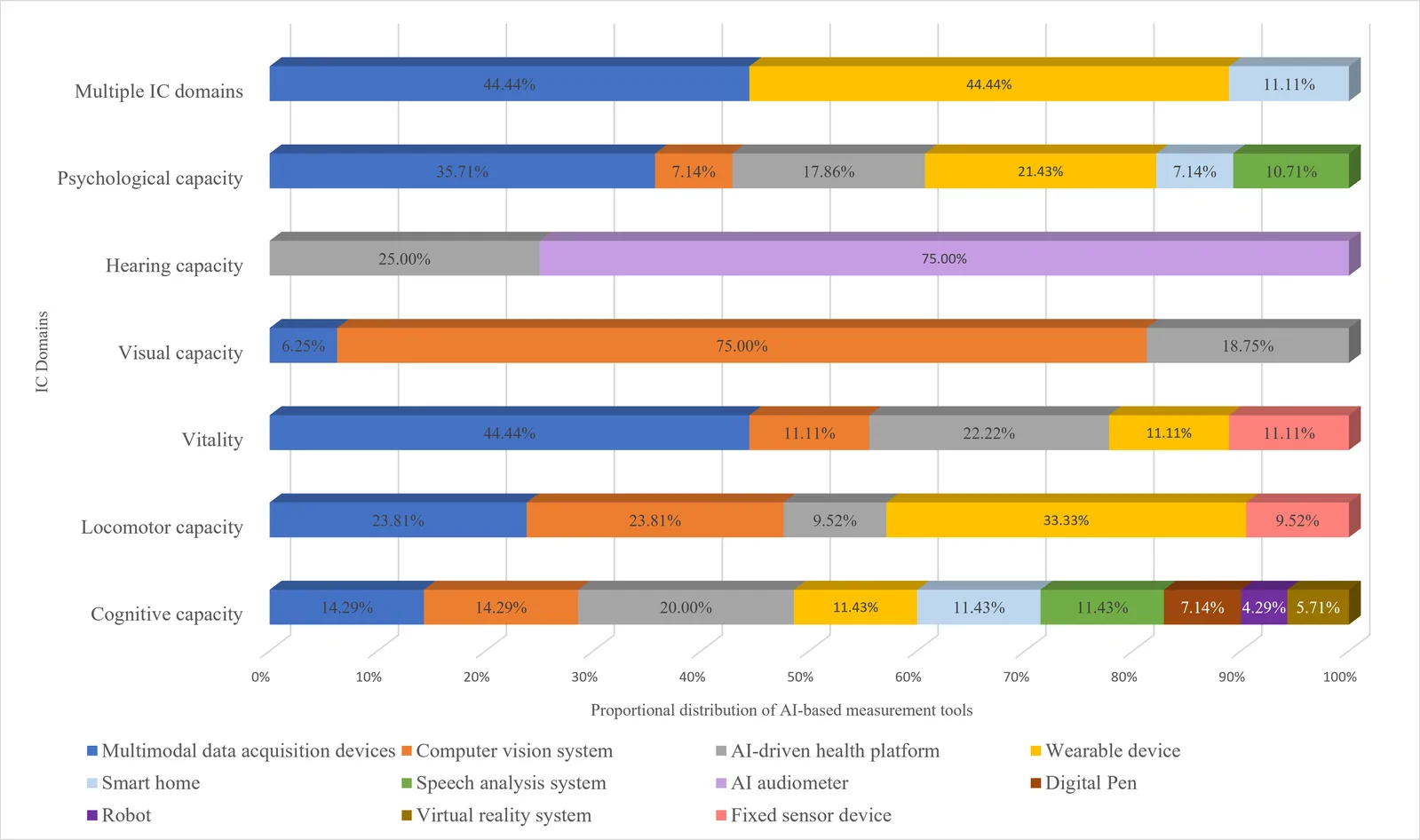
Distribution of AI-enabled digital devices and systems across intrinsic capacity (IC) domains. IC: intrinsic capacity.

These findings suggest that AI-enabled digital devices and systems have been increasingly applied to IC-related assessment, but their application remains uneven across domains and tool categories. The following sections describe the specific characteristics of each device category in the order of CV systems, AI-driven health platforms, wearable and fixed sensor devices, smart homes, speech analysis systems, AI audiometers, digital pens, robots, VR systems, and MDADs (Tables 3 and 4 and Multimedia Appendix 4).

**Table 3.** Characteristics of 10 AI-enabled digital devices and systems (n=127).

Number	Digital biomarkers	Name of AI-enabled digital devices and systems
Computer vision systems		
1‐12	Ocular structural features	Digital nonmydriatic fundus camera [36-47]
13‐21	Gait parameters	Kinect [48-51], RGB^a^ camera [52], Dual-task system [53,54], ELITE system [55], Webcams [56]
22‐24	Eye movement features	Eye-tracking system [57-59]
25‐27	Facial features	Camera NR^b^ [60], 3D Camera [61], Canon camera [62]
28	Digital task performance	Image of CDT^c^ from FHS^d^ Cohort [63]
29	Body posture features	Webcam [64]
30	Balance parameters	Kinect [65]
AI-driven health platforms		
1‐12	Digital task performance	dTMT^e^ [66], Online M-CRT^f^ [67], Computerized cognitive assessment system [68], EAVET^g^ system [69], “Drag-and-drop” [70], CognICA [71]^h^, AI Mini-Cog^i^ [72], dDSST^j^ [73], TAS^k^ system [74], PENSIEVE-AI [75], Intelligent application platform [76], Mobile application (no specific name reported) [77]
13‐17	Speech and language features	AI Dialogue Agent [78], Novoic App [79], Ellipsis Health App [80], DEPR-A chatbot [81], Naver Clova AI CareCall [82]
18 and 19	Eye movement features	Mobile eye-tracking system [83,84]
20 and 21	Ocular structural features	e-Paarvai platform [85], FOP NM 10 platform [86]
22	Ocular functional features	AI Pupillometer [87]
23 and 24	Hearing threshold level	Online ML^l^ audiometer [88], self-administered online hearing test (web application) [89]
25	Gait parameters	LINDERA Mobility Analysis mHealth^m^ app (LINDERA GmbH) [90]
26	Passive mobile sensing data	The Carat app [91]
27	Anthropometric parameters	Shaped AI 2D-photo app [92]
28	Facial features, anthropometric parameters	*R*+Dietitia platform [93]
Wearable devices		
1‐13	Gait parameters	Multisite sensors [27,28,94-96], trunk sensors [97-99], hearing aid with IMU^n^ [100], smart insole [101], joints sensors [102], and foot sensors [29,103]
14‐18	Physical activity features	Wrist sensors [104,106,107,186] and trunk sensors [105]
19‐21	fNIRS^o^ signals	fNIRS system [108-110]
22‐24	Physiological signals and physical activity features	Wrist sensors [111-113]
25	Plantar pressure parameters	Smart insole [114]
26	Gait parameters and physical activity features	PAMSys pendant sensor [30]
Fixed sensor devices		
1 and 2	Gait parameters	2D-motion capture apparatus [115], i-Walker [116]
3	Physiological signals	Noninvasive arterial applanation tonometry system [117]
Smart home		
1‐4	Digital task performance	CASAS^p^ smart home [118], event monitoring system [119], Alberdi A.’s smart homeCASAS smart home [120], and TraMiner system [121]
5‐7	Physical activity features and digital task performance	CASAS smart home [122,123], Mendez J. I.’s Smart Home Gamified smart home HMI^q^ [124]
8	Gait parameters	A real-time location sensor system [125]
9	Sleep parameters	SleepMove [126]
10	Physical activity features	Wi-Fi-based motion sensor system [26]
11	Physical activity features, sleep parameters, and physiological signals	DomoCare system [31]
Speech analysis systems		
1‐11	Speech and language features	Recording system [127-129], Phone-call system [130], Datasets recording [131-133], Computer with microphone [134,135], Sony digital recorder [136], Personal computer [137]
AI audiometers		
1‐6	Hearing threshold level	Automated ML–based audiometer [138,139], portable automated audiometer [140], Audiogram from NHANES^r^ [141], clinical audiometer [142], Automated ML–based BC^s^ audiometer [143]
Digital pens		
1‐3	Digital task performance	Tablet with digital pen [144-146]
4 and 5	Handwriting features	Livescribe Echo Pen [147], handwriting data from DARWIN^t^ dataset [148]
Robots		
1	Speech and language features	Softbank Pepper [149]
2 and 3	Digital task performance	RobiRobot [150], PaPeRo [151]
VR^u^ systems		
1‐3	VR task performance	Virtual shopping task system [152,153], virtual kiosk test system [154]
4	VR task performance and eye movement features	AI-VR digital cognitive measurement tool [155]

a RGB: red green blue

b NR: not reported.

c CDT: Clock Drawing Test.

d FHS: Framingham Heart Study.

e dTMT: digital Trail Making Test.

f M-CRT: Montreal Cognitive Recall Test.

g EAVET: emotional arousal and valence evaluation task.

h CognICA: Cognitivity’s Integrated Cognitive Assessment.

i Mini-Cog: Mini-Cognitive Assessment.

j dDSST: digital-Digit Symbol Substitution Test.

k TAS: Toronto Alexithymia Scale.

l ML: machine learning.

m mHealth: mobile health.

n IMU: inertial measurement unit.

o fNIRS: functional near-infrared spectroscopy.

p CASAS: Center for Advanced Studies in Adaptive Systems.

q HMI: human-machine interface.

r NHANES: National Health and Nutrition Examination Survey.

s BC: bone conduction.

t DARWIN: Diagnosis Alzheimer with handwriting.

u VR: virtual reality.

**Table 4.** Characteristics of multimodal data acquisition devices (n=34).

Number	Combination of devices	Digital biomarkers	Name of multimodal data acquisition devices
Cognition			
1,2	CV^a^+speech^b^	Eye movement^c^ and speech and language^d^	Eye-tracking system+recording device [156,157]
3	CV+speech	Speech and language, face^e^, and body posture^f^	Digital video recording system [158]
4	CV+pen^g^	Eye movement, gait^h^, and digital task^i^	Mobile eye-tracking system and ReadyGo+tablet with digital pen [159]
5	CV	Eye movement and gait	Mobile eye-tracking system+ReadyGo [160]
6	Wear^j^+AI platform^k^	Gait and digital task	Multisite sensors+finger tapping task platform [161]
7	Wear+AI platform	Gait, PA^l^, digital task, and speech and language	Limb sensors+RADAR AD^m^ study platform [162]
8	Wear+AI platform	Digital task, PA, PS^n^, PMS^o^, and speech and language	Apple Watch+Apple Research app [163]
9	Wear+SH^p^	PA and sleep^q^	Wrist sensors+Smart Mattress and Move [164]
10	Wear+fixed^r^	Gait, AP^s^, and sleep	electronic walkway, DXA^t^ scanner+portable polysomnography device [165]
Locomotor capacity			
11,12	Wear+ AI platform	Plantar pressure^u^ and balance^v^	Smart insole+video game-based app [166,167]
13	Wear	Plantar pressure and gait	Multisite sensors+smart insole [168]
14	Wear+CV	PA and gait	Kinect+wrist sensors [169]
15	CV+Fixed	Gait and balance	DSFT^w^ system [170]
Vitality			
16	CV+Wear	Gait and plantar pressure	Smartphone+Smart insole [171]
17	CV+fixed	Gait, GS^x^, and plantar pressure	Kinect+floor pressure plate, and digital dynamometer [172]
18	CV+fixed	Face and AP	3D Camera+BIA^y^ scale [173]
19	AI platform+fixed	PA and AP	DIEW app+Bluetooth weight scale, and BIA scale [174]
Vision			
20	AI platform+Robot	Digital task, ocular function^z^, ocular structure	DORIA platform+EyetLib Robotized scan [175]
Psychological capacity			
21	AI platform	PMS, PA, and speech and language	Custom Verily app [176]
22,23	Wear+AI platform	PA EMA^aa^	Wrist sensors+EMA app [177,178]
24	Wear+AI platform	PS, PA, and PMS	Wrist sensors+AWARE app [179]
25	Wear+AI platform	PA, PMS, and EMA	Oura Ring+AWARE app [180]
26	Wear+AI platform	PA and EMA	Wrist sensors+mental health platform [181]
27	Wear+AI platform	PA, PS, and digital task	Wrist sensors+RADAR-based app [182]
28	Wear+speech	PA and speech and language	Custom wrist-worn acoustic sensor [183]
29	CV+speech	Face and speech and language	Webcam+Computer microphone [184]
30	CV	Face and body posture^ab^	Canon camera (for video recording) [185]
Multiple IC domains			
31	CV+wear+fixed+ AI platform	Digital task, gait, balance GS, PS, and AP	Digital rehabilitation evaluation system [32]
32	Wear+CV	Gait	Wrist sensors+Smartphone and GoPro [33]
33	Wear+SH+ AI platform	Digital task, GS, PS, and PA	Wrist sensors+CAREUP^ac^ platforms+CAREUP-based smart home [34]
34	AI platform	Digital task and speech and language	ICOPE^ad^ MONITOR App+ICOPEBOT conversational robot [35]

a CV: computer vision system.

b Speech: speech analysis system.

c Eye movement: eye movement features.

d Speech and language: speech and language features.

e Face: facial features.

f Body posture: body posture features.

g Pen: digital pen.

h Gait: gait parameters.

i Digital task: digital task performance.

j Wear: wearable device.

k AI platform: AI-driven health platform.

l PA: physical activity.

m RADAR-AD: Remote Assessment of Disease and Relapse-Alzheimer Disease.

n PS: physiological signals.

o PMS: passive mobile sensing data.

p SH: smart home.

q Sleep: sleep parameters.

r Fixed: fixed sensor device.

s AP: anthropometric parameters.

t DXA: dual-energy X-ray absorptiometry.

u Plantar pressure: plantar pressure parameters.

v Balance: balance parameters.

w DSFT: Digital Senior Fitness Test.

x GS: grip strength.

y BIA: bioelectrical impedance analysis.

z Ocular function: ocular functional features.

aa EMA: ecological momentary assessment.

ab Body posture: body posture features.

ac CAREUP: an integrated care platform based on the monitoring of older individuals’ intrinsic capacity for inclusive health.

ad ICOPE: Integrated Care for Older People.

### CV Systems

A CV system refers to an AI-enabled digital system that uses CV technology to interpret and analyze visual data, such as images or videos. In this section, CV systems refer to the device/system category used to acquire and process visual data, whereas CV as an AI technique refers to the analytic approach used to extract or interpret visual features. These systems have been applied to support the assessment of vision, cognition, locomotor capacity, psychological capacity, and vitality [187,188]. A total of 30 studies on CV-based systems (hereinafter “CV systems”) were included. CV systems captured or derived 7 types of DBs, namely ocular structural features, gait parameters, eye movement features, facial features, digital task performance, balance parameters, and body posture features. Vision assessment was most commonly performed using CV systems. Large retinal image datasets acquired through nonmydriatic fundus cameras have been used to develop ML-based systems [45-47] for diabetic retinopathy (DR) screening, the retinal AI diagnosis system (RAIDS), and referral systems for vision-threatening diseases (VTDs). Depth cameras, such as Kinect, enable dual-task assessments like the Timed Up and Go (TUG) test and walking tests to extract gait parameters for identifying cognitive impairments, locomotor issues (eg, high fall risk and stroke), and depression [48,49,51,53,54,65,185]. CV systems were also extensively used in the cognitive domain. Eye-tracking technology, which measures gaze and saccadic movements [57-59,189,190], is emerging as a valuable tool for detecting cognitive impairments such as mild cognitive impairment (MCI) due to its noninvasive, low-burden, and scalable nature.

### AI-Driven Health Platforms

AI-driven health platforms (hereinafter “AI platforms”), combining computing devices and software, including smartphone and tablet apps, computer programs, online platforms, and chatbots, have seen wide application in health promotion and disease prevention among older adults [191]. A total of 28 studies on AI platforms were included.

The findings indicate that AI platforms can be used to assess all 6 IC domains by capturing 9 types of DBs, among which digital task performance was the most prevalent. “Digital task” can be understood as a digital interactive adaptation or an innovative digital game designed based on clinical routine scales or tests. Examples include the digital trail-making test [66], the emotional arousal and valence evaluation task (EAVET) system [69], and the “drag-and-drop” hand movement task [70]. Speech and language features are among the most commonly collected DBs through AI platforms. They are derived from dialogues between older adults and chatbots designed to support mental health, as well as from speech-based cognitive tasks.

AI platforms equipped with video recording functions are capable of capturing a range of DBs, such as facial features, ocular structural traits, and gait parameters. More specifically, using the smartphone’s camera to capture photos of the participant’s face [93], full-body or side-profile images [92], or eye images [85-87], or videos of walking [90] through customized mobile apps, it is possible to identify individuals at risk of malnutrition, patients with common eye diseases (such as cataracts and glaucoma), and individuals who have fallen. Moreover, facial 3D imaging combined with DL can noninvasively and accurately predict malnutrition and related complications in patients.

### Wearable and Fixed Sensor Devices

A wearable device is a body-worn sensor for data collection that has become a mature and cost-effective technology widely used in older adult care [192,193]. A total of 26 studies on wearable devices were included. Wearable devices were used to assess the IC domains of cognition, locomotor capacity, psychological capacity, and vitality, as well as multidimensional IC. DBs collected using wearable devices included gait parameters, physical activity features, functional near-infrared spectroscopy (fNIRS) signals, physiological signals (eg, heart rate and blood pressure), and plantar pressure parameters. Wearable devices can be classified, according to the body parts on which they are worn [11], into the following categories: limbs (58.14%), mixed (18.60%), trunk (11.63%), head (6.98%), and joints (4.65%). The most commonly used wearable devices were wrist and foot sensors. Devices such as smartwatches [106,107,111,112,162], ring sensors [180], pendant sensors [30], and smart insoles [101,114,166,168,171] enable prolonged remote monitoring, providing insights into trends. Additionally, wearable devices like wearable inertial sensor sets [29,95,168] and fNIRS cap [108-110] provided immediate measurement results in structured assessment settings.

In contrast to the widespread use of wearable devices, fixed sensor devices were used independently in only 3 studies, allowing for the assessment of locomotor capacity and vitality. In this review, we define fixed sensor devices as nonwearable, contact-based measurement systems installed in fixed spatial positions that acquire biomechanical or physiological signals through direct human-device interaction [194,195]. Examples of fixed sensor devices included a treadmill-based 2D motion-capture apparatus [115], a smart rollator [116], and a noninvasive arterial applanation tonometry system [117]. When used as part of MDADs, fixed sensors also included force plates, electronic handgrip dynamometers, and the BIA scale [33,34,180,185]. These fixed sensor devices provide higher measurement accuracy and standardization with lower dependence on user compliance.

### Smart Homes

A smart home is a residential environment built on connected sensors and automation technologies, in which AI serves as the core driver that transforms it from simple automation to intelligent adaptation [196,197]. Eleven studies on smart homes were included. Smart homes can measure IC and are applied primarily to the monitoring and assessment of cognition [118-123,125,126], while also tracking psychological functions [26,124] and multiple domains of IC [31]. Smart homes can construct dynamic user profiles [198] and digital twins [199] by integrating multimodal sensor data and AI modeling, enabling personalized and preventive health management. Smart homes are particularly suitable for older adults living alone [200]. In addition to health monitoring, they offer multiple functions such as environmental control, activity recognition and assistance, safety and emergency response, and emotional and social support, enabling continuous sensing and adaptive regulation to create a comfortable, safe, and health-oriented living environment [201,202].

### Speech Analysis Systems

Eleven studies on speech analysis systems were included. A speech analysis system typically comprises three stages: (1) speech acquisition, (2) extraction of acoustic or linguistic features (the latter via automatic speech recognition (R) or transcription), and (3) modeling and analysis with AI methods [203,204]. Speech analysis systems are limited to the assessment of cognitive and psychological capacities. Their function is to capture speech and language features through elicited speech tasks [127,130,133-135,137], neuropsychological interviews [63,128], and semistructured interviews [129,132,136], enabling the identification of individuals with cognitive impairment and depression. The speech and language features of cognitively impaired older adults are mainly marked by slow speech, frequent pauses, and degraded syntax and semantics, reflecting declines in cognitive processing and linguistic organization [205]. However, the features of older adults with depression are characterized by low energy, monotonous prosody, brief verbal content, and negative emotional tone, indicating reduced affective expression and diminished psychological vitality [206].

### AI Audiometers, Digital Pens, Robots, and VR Systems

Only a few studies included 4 types of single-domain or task-specific measurement tools, namely AI audiometers, digital pens, robots, and VR systems. AI audiometers, as reported in 6 studies [138-143], showed that ML enhanced hearing threshold estimation, increased testing efficiency, and facilitated the automation and broader applicability of professional audiometry.

Digital pens, robots, and VR systems were primarily used for the assessment of cognitive capacity. Digital pens were applied in motion-capturing handwriting systems to record drawing and writing tasks performed by participants [144-148]. Robots were used to administer clinical cognitive scales through interactive communication with participants [149-151]. VR systems were used to engage participants in immersive cognitive gaming tasks, while ML techniques transformed complex interaction data into digital diagnostic tools for the early detection of cognitive impairment [152-155].

### MDADs

Multimodal data refer to information derived from multiple perceptual channels or data modalities [207]. Accordingly, MDADs are formed by combining different types of AI-enabled digital devices and systems. A total of 34 studies on MDADs were included, demonstrating that MDADs can be used to assess psychological, cognitive, locomotor, vitality, visual, and multidimensional IC. The reviewed studies further indicate that the integration of multimodal data improves the classification performance of AI models [157,184].

The most common MDAD configurations involve combinations of wearable devices and AI-driven platforms. In psychological assessment, these combined systems capture both active and passive DBs, enabling the construction of digital phenotypes. The integration of ML further supports the classification and prediction of depression based on these multimodal digital phenotypes [177-182].

MDADs play a pivotal role in multidimensional IC assessment. A representative digital rehabilitation evaluation system developed in China enables participants to complete assessments of cognition, locomotor function, and vitality within approximately 30 minutes with minimal assistance [32]. In a multinational European project led by Poland, CAREUP is an integrated platform specifically designed for IC monitoring and prediction using multimodal data from wearable devices, questionnaires, and environmental sensors, with user-friendly interfaces for older adults and caregivers [34]. Another example is the INSPIRE ICOPE-CARE program in France, which includes the ICOPE Monitor mobile app and a customized chatbot (ICOPEBOT) [35]. The ICOPE monitor app collects self-reported and task-based data through touchscreen inputs, while the chatbot assesses users’ health status through natural language interactions via text or voice.

### DBs

A total of 21 types of DBs related to IC were identified from the 161 included studies. According to data collection methods, these DBs can be classified into 3 main types (Figure 8), including active (30.63%), passive (32.88%), and hybrid DBs (36.49%) [208,209].

**Figure 8.**
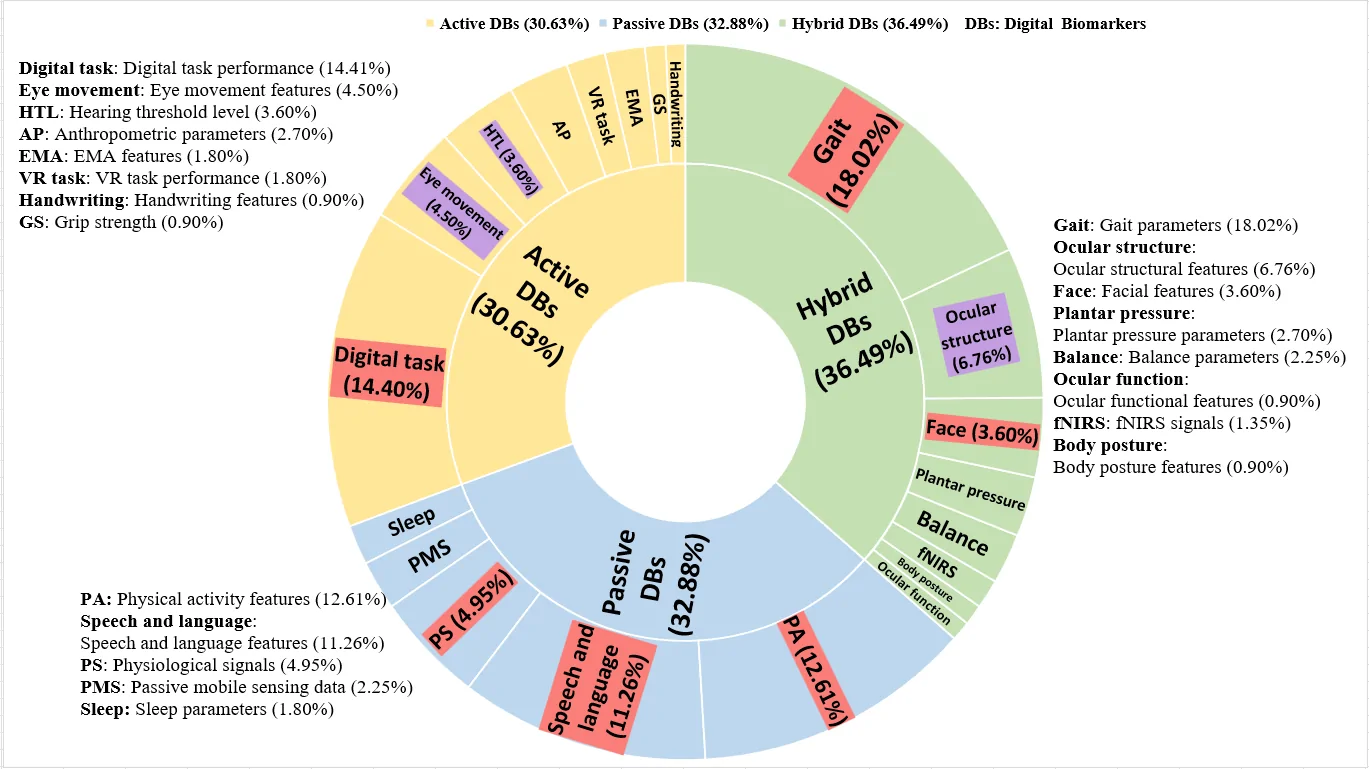
Sunburst diagram of digital biomarkers related to intrinsic capacity. AP: anthropometric parameter; DB: digital biomarker; EMA: ecological momentary assessment; fNIRS: functional near-infrared spectroscopy; GS: grip strength; HTL: hearing threshold level; PA: physical activity; PMS: passive mobile sensing; PS: physiological signal; VR: virtual reality.

Active interaction DBs are derived from data generated when participants actively perform tasks, respond to prompts, or engage in digital interactions and are used to assess specific functions [210]. Among the 8 active interaction DBs, the top 3 were digital task performance (14.41%), eye movement features (4.50%), and hearing threshold level (3.60%). Passive sensing DBs refer to quantifiable physiological or behavioral data that are automatically collected by digital devices or sensor systems through background or continuous monitoring, without requiring active participation from the user [209]. Among the 5 passive sensing DBs, the top 3 were physical activity features (12.61%), speech and language features (11.26%), and physiological signals (4.95%). Hybrid DBs, also termed semiactive DBs, represent a fusion of active and passive modalities [211]. For example, gait parameters derived from structured walking tasks or wearable sensor monitoring may integrate task-based and sensor-based data sources. Among the 8 hybrid DBs, the 3 most common were gait parameters (18.02%), ocular structural features (6.76%), and facial features (3.60%).

DBs were associated with multiple IC domains. A total of 8 overlapping DBs were identified (Figure 9). Overlaps across 2 IC domains were identified in the following DBs: plantar pressure parameters, physiological signals, speech and language features, and body posture features. Facial features overlapped across 3 domains. Gait parameters and physical activity features overlapped across 4 domains. Digital task performance overlapped across all 6 IC domains, suggesting its broad applicability across multidomain IC assessment.

**Figure 9.**
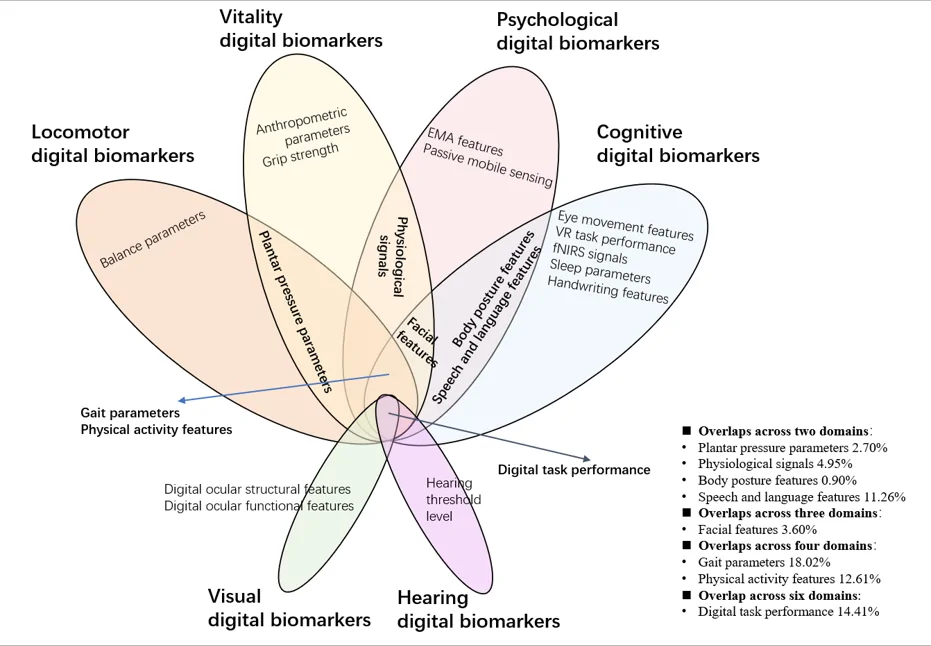
Venn diagram of digital biomarkers across intrinsic capacity domains. EMA: ecological momentary assessment; fNIRS: functional near-infrared spectroscopy; VR: virtual reality.

### Application of AI Techniques in IC

Five major categories of AI techniques were identified in the included studies [212,213], including ML, CV, NLP, robotics and chatbots, and knowledge representation and reasoning (KRR; Table 5 and Multimedia Appendix 2). In this review, CV was conceptually distinguished into 2 levels. At the system level, CV-based technologies are embedded within AI-enabled digital measurement systems used to acquire and process visual data (eg, cameras, depth sensors, or eye-tracking systems combined with analytic pipelines), as described in **“AI-Enabled Digital Devices and Systems” subsection of the Results**. At the algorithmic level, CV refers to computational techniques used for visual feature extraction and interpretation from images and videos, which are further reported in this section.

ML was the most frequently used AI technique. It encompasses supervised learning (SL), unsupervised learning (UL), DL, and reinforcement learning (RL). ML techniques extracted and integrated digital features, built classification models to distinguish healthy individuals from those with impaired IC, developed predictive models for IC decline risk, and analyzed feature weights to identify key contributing dimensions [164]. CV algorithms were mainly applied to image and video data for extracting visual features such as facial expressions, gait patterns, eye movements, and retinal imaging biomarkers. NLP techniques were used to process speech and text data, particularly in chatbot-based conversational assessments and speech-based cognitive evaluations. Robotics and chatbot systems were mainly applied for interactive assessment and automated administration of cognitive tasks. KRR techniques were used in a limited number of studies for structured reasoning and knowledge-based inference.

**Table 5.** Summary of AI techniques in intrinsic capacity.

Item	Data
Categories of AI techniques, n (%)	Frequency of appeared categories^a^ (n=284)
ML^b^	195 (68.66)
CV^c^	56 (19.72)
NLP^d^	22 (7.75)
Robotics and chatbots	6 (2.11)
KRR^e^	5 (1.76)
Combination of AI techniques, n (%)	Number of studies (n=161)
ML (SL^f^/UL^g^; DL; SL/UL; and DL^h^)	82 (50.93)
ML and CV	50 (31.06)
ML and NLP	13 (8.07)
ML, CV, and NLP	6 (3.73)
Chatbots	3 (1.86)
KRR	2 (1.24)
KRR and robotics	2 (1.24)
NLP and robotics	1 (0.62)
ML, NLP, and KRR	1 (0.62)
NLP	1 (0.62)

a Frequency of appeared categories: the frequency of occurrence of categories included in the study.

b ML: machine learning.

c CV: computer vision.

d NLP: natural language processing.

e KRR: Knowledge Representation and Reasoning.

f SL: supervised learning.

g UL: unsupervised learning.

h DL: deep learning.

A substantial proportion of studies used multiple AI techniques in combination, particularly by integrating ML with CV or NLP, or combining all 3 approaches. These multimodal AI strategies improved model performance by enabling complementary feature extraction and more robust interpretation across heterogeneous data modalities.

To further characterize the maturity of AI-enabled IC assessment, the predictive performance reported in the included studies was summarized descriptively according to IC domains (Table 6). Because the included studies varied considerably in target outcomes, datasets, reference standards, AI algorithms, and evaluation metrics, a quantitative synthesis of model performance was not appropriate. Therefore, reported AUC and accuracy values were summarized descriptively to provide an overview of the current level of evidence and the clinical readiness of AI-enabled measurement approaches.

**Table 6.** Descriptive summary of the reported predictive performance of AI-enabled digital devices and systems across intrinsic capacity domains. Studies reporting performance included studies reporting at least one quantitative performance metric. Performance ranges were summarized descriptively because of substantial heterogeneity in target outcomes, datasets, AI algorithms, and evaluation metrics across studies.

Main AI-enabled digital devices and systems	Common target outcomes	Reported AUC^a^ range	Reported accuracy range	Interpretation
Cognition (studies reporting performance, n=64)				
AI-driven health platforms, multimodal data acquisition devices, computer vision systems, wearable devices, smart homes, and speech analysis systems	MCI^b^, dementia, Alzheimer disease, cognitive impairment, and cognitive frailty	0.676‐1.000	0.688‐1.000	Most mature evidence with consistently favorable predictive performance
Locomotor capacity (studies reporting performance, n=17)				
Wearable devices, computer vision systems, multimodal data acquisition devices, and fixed sensor devices	Fall risk, gait impairment, mobility limitation, balance impairment, and walking ability	0.760‐1.000	0.674‐1.000	Strong predictive performance for gait- and mobility-related assessment
Vitality (studies reporting performance, n=8)				
Multimodal data acquisition devices, AI-driven health platforms, wearable devices, computer vision systems, and fixed sensor devices	Sarcopenia, frailty, malnutrition, body composition abnormality, and nutritional risk	0.700‐0.978	0.778‐0.968	Promising performance despite limited evidence
Vision (studies reporting performance, n=9)				
Computer vision systems, AI-driven health platforms, and multimodal data acquisition devices	Diabetic retinopathy, cataract, glaucoma, and retinal and optic nerve diseases	0.631‐0.958	0.920‐1.000	High performance in image-based disease detection
Hearing (studies reporting performance, n=4)				
AI audiometers and AI-driven health platforms	Hearing loss, hearing threshold estimation, and audiogram classification	0.963	0.890‐0.956	Promising performance from limited available evidence
Psychological capacity (studies reporting performance, n=21)				
Multimodal data acquisition devices, wearable devices, AI-driven health platforms, speech analysis systems, computer vision systems, and smart homes	Depression, depressive symptoms, anxiety, stress, mood disorders, and social isolation	0.656‐0.993	0.800‐0.951	Performance varied across target outcomes and data modalities
Multiple IC^c^ domains (studies reporting performance, n=7)				
Wearable devices, multimodal data acquisition devices, and smart homes	Multidomain IC decline, frailty, cognitive decline, and integrated IC monitoring	0.780‐0.800	0.810‐0.962	Integrated multidomain assessment remains underexplored

a AUC: area under the receiver operating characteristic curve.

b MCI:mild cognitive impairment.

c IC: intrinsic capacity.

AI-based measurement tools demonstrated generally favorable predictive performance across most IC domains. Cognition represented the most mature area of research, showing consistently favorable predictive performance across diverse AI-enabled digital devices and systems. Locomotor capacity also demonstrated strong predictive performance, particularly in gait- and mobility-related assessment. In contrast, vitality, hearing, and multidomain IC assessment were supported by relatively limited evidence despite promising reported performance. Psychological capacity exhibited greater variability in predictive performance, reflecting differences in target outcomes, data modalities, and reference standards across studies. These findings indicate that AI-enabled IC assessment has reached a relatively mature stage in cognition and locomotor evaluation, whereas further validation is still required for underrepresented domains and integrated multidomain assessment.

## Discussion

### Principal Findings

This scoping review systematically mapped the current landscape of AI-based measurement tools for IC by synthesizing evidence on AI-enabled digital devices and systems, DBs, and AI techniques. Unlike previous reviews that primarily examined individual AI technologies or disease-specific applications in older adults, the present review adopts IC as the organizing framework and systematically integrates these 3 complementary dimensions into a coherent analytical framework. Rather than focusing on individual algorithms or disease-specific applications, this review characterizes how AI technologies are being applied across different IC domains and evaluates their current level of evidence and clinical maturity.

Several important findings emerged from this review. First, AI-based measurement tools for IC have emerged as a multidisciplinary field, bringing together digital sensing technologies, multimodal data acquisition, and AI-driven analytical approaches to support more objective and scalable IC assessment. The convergence of these technologies has enabled continuous, objective, and scalable assessment of IC beyond conventional clinic-based evaluations. This development is consistent with the growing emphasis on digital health technologies to support healthy aging and aligns with the WHO framework, which recognizes IC as a core determinant of healthy aging and promotes person-centered care through the ICOPE approach [1].

Second, DBs have become a central component of AI-based IC assessment. Compared with traditional questionnaire-based or episodic clinical assessments, multimodal DBs provide objective, continuous, and ecologically valid measures of IC. Their integration with AI algorithms reflects a transition from isolated measurements toward comprehensive characterization of functional health, consistent with recent advances in DB research for healthy aging and geriatric care [15,18,214].

Third, the maturity of AI-based measurement tools varied considerably across IC domains. Cognition and locomotor capacity represented the most extensively investigated areas, supported by relatively mature measurement systems and consistently favorable predictive performance. In contrast, evidence for vitality, hearing, and multidomain IC assessment remained comparatively limited, despite growing research interest. The evidence synthesized in this review indicates that AI-based measurement tools for IC are transitioning from proof-of-concept research toward clinical applications. Although encouraging progress has been achieved, important questions regarding evidence maturity and clinical translation remain [215]. These issues are discussed in the following sections.

### Current Maturity of AI-Based Measurement Tools for IC

The evidence synthesized in this review indicates that the maturity of AI-based measurement tools varies substantially across IC domains. Rather than reflecting differences in the intrinsic capability of AI technologies, this variation appears to be more closely associated with the availability of measurable DBs, the degree of standardization in clinical assessment, and the feasibility of collecting reliable longitudinal data [6,15,18]. Consequently, the current landscape of AI-based IC assessment is characterized by uneven evidence maturity, with cognition and locomotor capacity representing the most developed domains, whereas vitality, hearing, and multidomain IC assessment remained at relatively early stages of development.

Cognition and locomotor capacity appear to have progressed more rapidly than other IC domains because they are supported by relatively well-established clinical assessment frameworks, objective DBs, and mature measurement technologies. In cognition, standardized instruments such as the Mini-Mental State Examination (MMSE) and Montreal Cognitive Assessment (MoCA) provide widely accepted reference standards for AI model development and validation [216]. Locomotor capacity can be assessed using objectively quantifiable indicators, including gait, balance, mobility, and physical activity, which are readily acquired through wearable sensors and CV systems [217]. The combination of standardized clinical reference standards and continuously measurable DBs has facilitated the development of robust AI models, contributing to the comparatively higher maturity of AI-based measurement tools in these domains. Similar trends have been reported in previous reviews of digital health and AI applications in older adults, which consistently identify cognition and mobility as the most advanced domains for technology-assisted assessment and monitoring [17,218].

The comparatively limited evidence for vitality, hearing, and multidomain IC assessment appears to be driven primarily by challenges in defining and measuring these domains rather than by limitations inherent to AI technologies themselves. Vitality remains the least standardized IC domain, with studies adopting diverse indicators related to nutritional status, muscle strength, frailty, body composition, and physiological reserve, resulting in substantial heterogeneity in assessment approaches and outcome definitions [219,220]. Hearing assessment has predominantly focused on disease-specific applications, such as automated audiometry or hearing impairment detection, rather than comprehensive evaluation within the IC framework [221,222]. Multidomain IC assessment, although conceptually aligned with the WHO ICOPE model, requires the integration of heterogeneous data sources, harmonized assessment across multiple IC domains, and clinically meaningful composite outcomes [223]. These methodological and conceptual challenges continue to slow the development of comprehensive AI-based multidomain IC assessment despite growing research interest.

### Toward Clinical Translation of AI-Based Measurement Tools for IC Assessment

Although AI-based measurement tools have demonstrated encouraging performance across several IC domains, routine clinical adoption remains limited. The findings of this review suggest that the principal barriers to translation are no longer solely related to algorithm development but to the broader process of translating AI into clinically meaningful IC assessment. Successful clinical translation requires 3 interdependent components: standardized IC assessment, robust clinical evidence, and effective integration into routine health care delivery.

The first prerequisite for clinical translation is standardized IC assessment. Although the WHO ICOPE framework provides a conceptual foundation for evaluating IC, substantial variation remains in the operational definitions, assessment instruments, and composite scoring approaches adopted across studies, particularly for vitality and multidomain IC. Such heterogeneity not only limits comparisons among studies but also hinders the establishment of robust reference standards for AI model development and evaluation [224]. Because AI models learn from the labels generated by existing assessment frameworks, inconsistency in IC measurement inevitably affects model reproducibility, transferability, and comparability across different health care settings. Clinical translation therefore requires greater harmonization of IC assessment protocols to establish reliable reference standards that support both clinical practice and AI model development [225].

The second prerequisite is the generation of robust clinical evidence. Most studies included in this review were conducted using relatively small datasets from single institutions or geographically restricted populations. Although many studies reported encouraging internal performance, evidence regarding external validation, prospective evaluation, and real-world clinical effectiveness remains limited. Reliable clinical adoption requires AI models to demonstrate consistent performance across diverse populations, health care systems, and care settings. Recent reporting and evaluation guidelines for clinical AI have similarly emphasized that rigorous external validation, transparent reporting, and prospective evaluation are fundamental steps before AI technologies can be considered ready for routine clinical use [218,226].

The third prerequisite is successful implementation within clinical workflows. Accurate prediction alone is unlikely to improve health care unless AI-based measurement tools can be integrated into routine IC assessment and clinical decision-making. In practice, this requires interoperability with existing digital health platforms, compatibility with electronic health records, interpretable outputs that support clinicians’ decisions, and alignment with person-centered care pathways such as the WHO ICOPE framework [223]. Equally important, successful implementation depends on the acceptance of older adults, health care professionals, and health care organizations, as well as adequate digital infrastructure and implementation strategies that support sustainable use in routine practice [227]. These implementation factors are increasingly recognized as key determinants of whether AI technologies can generate meaningful improvements in healthy aging care beyond proof-of-concept research. The 3 prerequisites outlined above indicate that future progress should be evaluated not only in terms of predictive performance but also with regard to standardization, clinical evidence, and implementation readiness. Advancing these areas in parallel will be essential if AI-based measurement tools are to develop into reliable instruments for person-centered IC assessment within routine health care systems.

### Implications for Future Research and Clinical Practice

The findings of this review have several important implications for future research. Rather than continuing to develop isolated AI algorithms for individual IC domains, future studies should prioritize the establishment of standardized IC assessment frameworks, large multicenter datasets, and longitudinal cohorts that enable consistent evaluation of IC trajectories over time [228-230]. Such efforts will facilitate more robust comparisons across studies while supporting the development of AI-based measurement tools that are generalizable across diverse populations and health care settings. In addition, increasing attention should be given to multimodal approaches that integrate physiological, behavioral, speech, mobility, and environmental data to better capture the multidimensional nature of IC [231,232].

From a clinical perspective, AI-based measurement tools may extend IC assessment beyond scheduled clinic visits by enabling more frequent observation of changes in functional capacity. In community and primary care settings, these tools could support early identification of individuals who may require further assessment or intervention, while home-based monitoring may help detect changes between routine visits and track responses over time. These applications are consistent with the preventive and person-centered orientation of the WHO ICOPE framework [233] and may contribute to a broader shift toward more proactive management of functional health in older adults. Realizing this potential will require close collaboration among clinicians, geriatric researchers, data scientists, engineers, and policymakers to ensure that technological development remains aligned with clinical needs, implementation requirements, and the goals of healthy aging. Such interdisciplinary collaboration will be important for translating AI-based measurement tools into sustainable and acceptable approaches across different care settings.

### Strengths and Limitations

A major strength of this review is that it examines AI-based measurement tools from the perspective of IC rather than individual AI technologies or disease-specific applications. By integrating AI-enabled digital devices and systems, DBs, and AI techniques within a unified analytical framework, this review provides a comprehensive overview of the current evidence landscape while offering a structured perspective on evidence maturity and clinical translation. This approach also enables comparison across the 6 IC domains and helps identify areas in which evidence and tool development remain limited.

Several limitations should also be acknowledged. First, relatively few AI-based measurement tools have been specifically developed for IC assessment. Most included studies evaluated AI technologies originally designed for disease-specific or function-specific assessment, and their relevance to IC was interpreted according to the WHO IC framework. Although this reflects the current developmental stage of the field, it may contribute to conceptual heterogeneity when interpreting the maturity of AI-based IC assessment. Second, substantial methodological heterogeneity in study populations, AI methodologies, outcome definitions, and IC assessment approaches limited direct comparisons across studies. Finally, because AI and digital health technologies continue to evolve rapidly, newly emerging evidence may not have been captured in this review. Consequently, the conclusions should be interpreted as reflecting the current state of the evidence and should be updated as the field continues to evolve.

### Conclusions

This scoping review provides a 3D synthesis of AI-enabled digital devices and systems, DBs, and AI techniques across the 6 WHO-defined domains of IC. Unlike previous reviews that focused primarily on individual technologies, DBs, specific diseases, or single functional domains, this review examines these components together within the broader IC framework and compares the distribution and maturity of evidence across domains. The review identifies cognition and locomotor capacity as the most developed areas, highlights evidence gaps in vitality, hearing, and multidomain assessment, and outlines priorities for standardization, external validation, and integrated IC assessment. In practice, AI-based measurement tools may complement conventional assessment by supporting community screening, primary care assessment, and longitudinal monitoring in home and routine care settings. Their successful translation into person-centered healthy aging care will depend on robust clinical validation, integration into existing care pathways, adequate digital infrastructure, and implementation approaches that address the needs and preferences of older adults.

## Supplementary material

10.2196/90411 Multimedia Appendix 1 Database search strategy.

10.2196/90411 Multimedia Appendix 2 Four supplementary figures.

10.2196/90411 Multimedia Appendix 3 Characteristics of the 161 included studies.

10.2196/90411 Multimedia Appendix 4 Characteristics of AI-based intrinsic capacity measurement tools (expanded version).

10.2196/90411 Multimedia Appendix 5 Glossary.

10.2196/90411 Checklist 1 PRISMA-ScR checklist.

## Abbreviations

BIA: bioelectrical impedance analysis
CNKI: China National Knowledge Infrastructure
CV: computer vision
DB: digital biomarker
DL: deep learning
DR: diabetic retinopathy
EAVET: emotional arousal and valence evaluation task
EEG: electroencephalography
EMA: ecological momentary assessment
fNIRS: functional near-infrared spectroscopy
IC: intrinsic capacity
ICOPE: integrated care for older people
KRR: knowledge representation and reasoning
MCI: mild cognitive impairment
MDAD: multimodal data acquisition device
ML: machine learning
MMSE: Mini-Mental State Examination
MoCA: Montreal Cognitive Assessment
MRI: magnetic resonance imaging
NLP: natural language processing
OCT: optical coherence tomography
PCC: population-concept-context
PET-CT: positron emission tomography–computed tomography
PRISMA-ScR: Preferred Reporting Items for Systematic Reviews and Meta-Analyses extension for Scoping Reviews
RAIDS: retinal AI diagnosis system
RL: reinforcement learning
SL: supervised learning
TUG: Timed Up and Go
UL: unsupervised learning
VR: virtual reality
VTD: vision-threatening disease
WHO: World Health Organization
