# Supplementary material for: AI-Based Measurement Tools for Intrinsic Capacity in Older Adults: Scoping Review
Source: J Med Internet Res. 2026 Oct 5;28:e90411. doi: 10.2196/90411 (PMC13639135; doi:10.2196/90411)
Supplement: Supplementary file 1 [file jmir-v28-e90411-s003.docx]

Multimedia Appendix 3

The characteristics of included studies(N=161).

Contents

1.	The domain of IC: Cognition (70)	1

2.	The domain of IC: Locomotor capacity (21)	8

3.	The domain of IC: Vitality (9)	11

4.	The domain of IC: Vision (16)	12

5.	The domain of IC: Hearing (8)	15

6.	The domain of IC: Psychological capacity (28)	16

7.	The domain of IC: multiple IC domains (9)	19

Description of the file:

Multimedia Appendix 3 presents a table summarizing the characteristics of the 161 included studies. The studies are listed in the following order according to the intrinsic capacity domains: Cognition, locomotor capacity, vitality, vision, hearing, psychological capacity, and multiple IC domains. The table includes the following information for each study: author, year, country, number of participants, mean age (age range), percentage of female participants, participants’ IC condition, setting, and type of AI-enabled digital device.

Table 2 The characteristics of included studies.

| 1. The domain of IC: Cognition (70) | | | | | | |
| --- | --- | --- | --- | --- | --- | --- |
| **Number) Author (Year) Country [Ref]** | **Number of participants** | **Mean age (Range age)** | **Female %** | **Participants’ IC condition** | **Setting** | **Type of AI-enabled digital devices** |
| 1) Dawadi P. N. et.al. (2013) America [118] | N=179 | <45-75+ | 78.77% | Healthy, MCI,  Dementia | Lab | Smart home |
| 2) König A. et al. (2015) France [119] | N=49 | 77.7 ± 7.3 | 53.10% | Healthy, MCI, AD | Lab | Smart home |
| 3) Dawadi P. N. et al. (2016) America [122] | N=18 | 84.71 ± 5.24 (73-92) | 27.78% | Healthy, At risk,  CI (MCI/AD) | Lab | Smart home |
| 4) Dahmen J. et al. (2017) America [66] | N = 54 | 68.4 ± 9.3 (50–93) | 80% | Health, neurological conditions | Lab,  Clinical | AI-driven health platforms |
| 5) Kawa J. et al. (2017) Poland [147] | N=74 | MCI: 76.1 ± 5.8 (61–85),  Health: 74.8 ± 5.7 (65-85) | 12.16% | Healthy, MCI | Clinical | Digital pen |
| 6) Alberdi A. et al. (2018) Spain, America, France [120] | N=29 | Healthy: 82.85 (73–92),  At risk: 86.20 (73–97),  MCI: 84.50 (82–90) | 83% | Healthy, At risk, MCI | Lab | Smart home |
| 7) Ghosh S. et al. (2018) Germany, Italy [104] | N=140 | Healthy:  69.49 ± 4.15 (65-75),  Dementia:  83.79 ± 6.2 (72-89) | Healthy: 50%  Dementia:43% | Healthy, Dementia | Community,  Clinical | Wearable device |
| 8) Toth L. et al. (2018) Hungary [127] | N=84 | MCI:  73.08 ± 7.95 (55–93),  Healthy:  64.13 ± 7.08 (57–84) | 65.47% | Healthy, MCI | Clinical | Speech analysis system |
| 9) Bergeron M. F. et al. (2019) America [67] | N=4645 | 21-99 | NR | Healthy, At risk | Online, Home | AI-driven health platform |
| 10) Fraser K. C. et al. (2019) Sweden [156] | N=55 | Healthy: 67.8 ± 7.7,  MCI: 70.6 ± 5.8;(50–79) | 63.64% | Healthy, MCI | Lab | Multimodal data acquisition devices |
| 11) Matsuura T. et al. (2019) Japan [53] | N=90 | 55-95 | 75.60% | Healthy, MCI | Lab,  Clinical | Computer vision system |
| 12) Jia Y. et al. (2019) China [68] | N=359 | 63(57–69) | 67.70% | Healthy, MCI | Community, Clinical | AI-driven health platform |
| 13) Mengoudi K. et al. (2020) Britain [57] | N=205 | Controls A: 62.03± 7.79,  Controls B: 69-71,  Dementia: 68.9±9.16 | 53.17% | Healthy, CI, Dementia | Lab | Computer vision system |
| 14) Rutkowski T.M. et al. (2020) Japan [69] | N=35 | 73.5± 4.85 | 62.90% | Health, MCI | Lab | AI-driven health platform |
| 15) Tang F. et al. (2020) America [78] | N=41 | 80.43 (≥70) | 71% | Health, MCI | Online, Home | AI-driven health platform |
| 16) Thomas J.A. et al. (2020) America [128] | N=135 | 73± 16 | 51% | Health, CI | Lab,  Clinical | Speech analysis system |
| 17) Amini S. et al. (2021) America [63] | N=3,423 | Health:61.8 ± 13.2,  Dementia: 82.1 ± 7.3 | Health: 54.3%, Dementia: 53.75% | Health, CI, Dementia | Lab,  Clinical | Computer vision system |
| 18) Haque R. U. et al. (2021) America [83] | N=250 | 72.3 ± 9.1 | 56% | Healthy, MCI, AD | Clinical | AI-driven health platform |
| 19) Souillard-Mandar W. et al. (2021) America [144] | N=1833 | 69.9 | 50.85% | Healthy, CI | Clinical | Digital Pen |
| 20) Tsai C. F. et al. (2021) China [152] | N=12 | MCI/AD: 74.83 ± 4.07,  Healthy: 70.17 ± 5.15 | 50% | Healthy, MCI | Lab,  Clinical | Virtual reality system |
| 21) Umeda-Kameyama Y. et al. (2021) Japan [60] | N=238 | Healthy: 75.8±6.0,  CI: 80.6±6.5 | 59.67% | Healthy, CI | Lab,  Clinical | Computer vision system |
| 22) Yang D. et al. (2021) Korea [108] | N=24 | MCI: 69.27±7.09  Health: 68.33 ±4.69 | 53.30% | Healthy, MCI | Clinical | Wearable device |
| 23) Yoshii K. et al. (2021) Japan [149] | N=94 | HC: 70.6±4.9,  MCI: 74.0±5.4 | 50% | Healthy, MCI | Lab,  Clinical | Robot |
| 24) Zhang Y. et al. (2021) China [70] | N=61 | MCI: 68.25±6.15,  Healthy: 67.36±4.76  (50-81) | 52.46% | Healthy, MCI | Lab | AI-driven health platform |
| 25) Chang Y. L. et al. (2022) China [150] | N=66 | Healthy young:  21.52 ± 2.24,  Healthy older:  71.11 ± 3.55,  MCI: 73.53 ± 6.39 | 51.52% | Healthy, MCI | Lab | Robot |
| 26) Diaz-Asper C. et al. (2022) America [130] | N=91 | 73.67± 6.94(57–93) | 55% | Healthy, MCI, AD | Online, Home | Speech analysis system |
| 27) Fristed E. et al. (2022) Britain [79] | N=115 | 69.63(50-85) | 54.78% | Healthy, MCI, AD | Online, Home | AI-driven health platform |
| 28) Ho T.K. et al. (2022) Korea [109] | N=140 | Healthy: 72.7 ± 5.3,  aAD: 74.5 ± 4.3,  AD: 75.4 ± 6.8 | 50.71% | Healthy, aMCI, AD | Lab,  Clinical | Wearable device |
| 29) Kobayashi M. et al. (2022) Japan [145] | N=144 | 73.9± 5.2 | 53.50% | Healthy, MCI, AD | Clinical | Digital Pen |
| 30) Ruengchaijatuporn N. et al. (2022) Thailand [146] | N=918 | 67 (55–89) | 77% | Healthy, MCI | Clinical | Digital Pen |
| 31) Seifallahi M. et al. (2022) America [48] | N=85 | Health: 68.47 ± 2.60,  AD: 75.75 ± 7.17 | 54.12% | Healthy, AD | Clinical | Computer vision system |
| 32) Sun J. et al. (2022) China [58] | N=210 | 40-92 | NR | Healthy, AD | Clinical | Computer vision system |
| 33) Zolfaghari S. et al. (2022) Italy [121] | N=153 | 60-74 | NR | Healthy, MCI, Dementia | Lab | Smart home |
| 34) Alsubai S. et al. (2023) Saudi Arabia [123] | N=79 | 66 | NR | Healthy, Dementia | Lab | Smart home |
| 35) Amini S. et al. (2023) America [131] | N=1084 | Health:77.2±9.7,  MCI:81.6±8.0,  Dementia: 85.1±7.5 | 56.82% | Healthy, MCI, Dementia | Lab,  Clinical | Speech analysis system |
| 36) Faruk T. et al. (2023) Canada [125] | N=10 | 62-98 | 90% | Health, MCI, Severe CI | Clinical | Smart home |
| 37) Khosroazad S., Abedi A. et al. (2023) America [126] | N=40 | Health: 72.2±1.6,  MCI: 74.3±1.2 | 75% | Health, MCI | Lab,  Clinical | Smart home |
| 38) Khosroazad S., Gilbert C. F. et al.  (2023) America [163] | N=95 | 60-90 | 70.53 % | Healthy, MCI | Home | Multimodal data acquisition devices |
| 39) Kim S.Y. et al. (2023) Korea [153] | N=51 | MCI: 72.68 ± 7.75,  Healthy: 70.95 ± 6.02 | 50.98% | Healthy, MCI | Lab | Virtual reality system |
| 40) Modarres M. et al. (2023) Britain [71] | N=86 | 76.0(56–89) | NR | Healthy, At risk, MCI, Dementia | Community,  Online, Home | AI-driven health platform |
| 41) Leng F. et al. (2023) China [72] | N=217 | 73.2 ± 7.4 (60–86) | 57.14% | Health, MCI, Dementia | Clinical,  Online, Home | AI-driven health platform |
| 42) Peng M. et al. (2023) China [134] | N=215 | 50–80 | 49.75% | Health, MCI | Lab,  Clinical | Speech analysis system |
| 43) Ambrosini E. et al. (2024) Italy, Spain [135] | N=266 | ≥65 | 72.56% | Healthy, MCI, moderate to severe CI | Clinical | Speech analysis system |
| 44) Farrell B. et al. (2024) Iran [52] | N=55 | Healthy: 68.33 ± 2.15,  MCI: 69.76 ± 6.45 | Healthy: 53%,  MCI: 56% | Healthy, MCI | Lab | Computer vision system |
| 45) Kang L. et al. (2024) China [148] | N=174 | 60–85 | NR | Healthy, AD | Lab | Digital pen |
| 46) Li Q. et al. (2024) China [84] | N=273 | 70.46(50–90) | 61.20% | Healthy, AD | Lab | AI-driven health platform |
| 47) Lin J. et al. (2024) China [160] | N=1481 | CI: 69.76 ± 6.54,  Healthy: 68.82 ± 5.84 | 65.90% | Healthy, MCI, Dementia | Community | Multimodal data acquisition devices |
| 48) Obuchi S. P. et al. (2024) Japan [94] | N=879 | 73.5 | 60.60% | Health, CI | Community | Wearable device |
| 49) Poor F. et al. (2024) America [158] | N=155 | >75 | NR | Healthy, MCI | Lab | Multimodal data acquisition devices |
| 50) Pourramezan Fard A. et al. (2024) America [132] | N=68 | >75 | NR | Healthy, MCI | Lab | Multimodal data acquisition devices |
| 51) Segev O. et al. (2024) Israel [73] | N=100 | 74.2± 6 | 37% | Health, CI | Clinical | AI-driven health platform |
| 52) Wang Y. et al. (2024) China [154] | N=1328 | Healthy: 71.25±6.20,  MCI: 72.14±8.66 | 59.94% | Healthy, MCI | Lab | Virtual reality system |
| 53) Zuo F. et al. (2024) China [59] | N=106 | AD: 68±7(54-80),  Health: NR | AD:60.5%,  Health: NR | Health, AD | Lab,  Clinical | Computer vision system |
| 54) Fan L. et al. (2025) China [186] | N=3363 | ≥60 | 52.10% | Healthy, CI | Community,  Clinical | Wearable device |
| 55) Gramkow M. H. et al. (2025) Denmark [105] | N=170 | 55–95 | 44.71% | MCI, Mild dementia, Moderate dementia, Healthy | Home | Wearable device |
| 56) Guimarães V. et al. (2025) Portugal [161] | N=53 | Healthy: 72.59± 4.95,  MCI: 75.88 ± 5.49 | 56.60% | Healthy, MCI | Lab | Multimodal data acquisition devices |
| 57) Huang G. et al. (2025) Australia [74] | N=2351 | 65.35±7.51 (50-89) | 71.76% | Health, SCD, MCI, AD | Home,  Lab | AI-driven health platform |
| 58) Lentzen M. et al. (2025) Germany [162] | N=229 | 69.2±7.7 | 49.30% | Health, Pre AD, Pro AD, Mild AD | Home | Multimodal data acquisition devices |
| 59) Liew T. M. et al. (2025) Singapore [75] | N=1758 | 72 (65–101) | 63.59% | Healthy, MCI, Dementia | Community | AI-driven health platform |
| 60) Najafi B. et al. (2025) America [64] | N=413 | 64.24 ± 7.64 | 67.10% | Healthy, MCI | Lab | Computer vision system |
| 61) Nishiura Y. et al. (2025) Japan [151] | N=51 | 80.5±5.6(68–91) | 74.50% | Health, CI | Community,  Home | Robot |
| 62) Pu Z. et al. (2025) China [110] | N=246 | Healthy: 61.44±4.46,  SCD: 63.30±6.54,  MCI: 64.80±6.60 | 50.00% | Healthy, SCD, MCI | Clinical | Wearable device |
| 63) Satake Y. et al. (2025) Japan [54] | N=346 | neurocognitive  disorders: 78.0 ± 8.7,  Healthy: 73.8 ± 3.8 | 59.25% | Healthy, AD, Dementia with Lewy bodies, MCI | Clinical | Computer vision system |
| 64) Shah H. A. et al. (2025) Finland [157]59 | N=78 | Healthy: 71±5.1,  MCI: 72±6.4,  AD: 61.9±13 | 53.85% | Healthy, MCI, AD | Clinical | Multimodal data acquisition devices |
| 65) Zhang X. et al. (2025) China [159] | N=1083 | 70 (65-93) | 55% | Healthy, CI, MCI, Dementia | Community,  Clinical | Multimodal data acquisition devices |
| 66) Zhang Z. et al. (2025) China [133] | N=156 (ADReSS), External test: N=443 (Pitt) | ADReSS: 50–79 | 55.13%(ADReSS), 63.21%(Pitt) | Healthy, AD | Community,  Lab | Speech analysis system |
| 67) Butler, P. M. et al. (2025) America [164] | N=23,004 | 58.0 ± 15.6 | 64.40% | Healthy, MCI | Community,  Online, Home | Multimodal data acquisition devices |
| 68) Park I. et al. (2025) South Korea [165] | N=80 | 74.6±5.74 | 72.50% | Early-stage MCI， Late-stage MCI | Clinical | Multimodal data acquisition devices |
| 69) Moon G. et al. (2025) South Korea [96] | N=139 | 75.0 (72.0–83.0) | 64.75% | Healthy; MCI; mild dementia; moderate dementia | Lab | Wearable device |
| 70) Fang L. et al. (2025) China [155] | N=428 | GHABS: 64.2;  STAR: 63.6 | GHABS: 67.1%;  STAR: 67.5% | Healthy; MCI; dementia | Community | virtual reality systems |

Table 2 (Continued).

| 2. The domain of IC: Locomotor capacity (21) | | | | | | |
| --- | --- | --- | --- | --- | --- | --- |
| **Number) Author (Year) Country [Ref]** | **Number of participants** | **Mean age (Range age)** | **Female %** | **Participants’ IC condition** | **Setting** | **Type of AI-enabled digital devices** |
| 71) Lai D. T. H. et al. (2008) Australia [115] | N=23 | Healthy: 71.0±2.1  Fallers: 72.2 ±3.1 | NR | Healthy, Fallers | Lab | Fixed sensors device |
| 72) Kargar A. H. et al. (2014) America [51] | N=12 | 65–90 | NR | Low risk of fall,  High risk of fall | Lab | Computer vision system |
| 73) Mannini A. et al. (2016) Italy [95] | N=42 | HD: 54.3 ± 12.2  PS: 61.3 ± 13.0  Healthy: 69.7 ± 5.8 | 42.86% | Healthy, locomotor capacity impaired  (post stroke, Huntington’s disease) | Lab,  Clinical | Wearable device |
| 74) Altilio R. et al. (2017) Italy [55] | N=115 | 56.65 (23–86) | 45.20% | Healthy, locomotor capacity impaired  (Arthropathic, Neurologic) | Lab | Computer vision system |
| 75) Ballesteros J. et al. (2017) Spain [117] | N=19 | 68 ± 9.25(50–80) | 68.40% | Low risk of fall,  Moderate risk of fall,  High risk of fall | Clinical | Fixed sensors device |
| 76) Dubois A. et al. (2017) Switzerland [49] | N=37 | 83.63 (61–93) | 62.16% | locomotor capacity impaired (FRAIL),  Low risk of falls,  High risk of falls (TUG) | Clinical | Computer vision system |
| 77) Howcroft J. et al. (2017) Canada [168] | N=75 | 75.2 ± 6.6 | 57.89% | Healthy, Fallers | Lab | Multimodal data  acquisition devices |
| 78) Dubois A. et al. (2019) Switzerland [65] | N=84 | Young: 24.5 ± 2.4 (21–29)  Elderly: 72.9 ± 5.2 (65–85) | 71.43% | low/no risk of fall,  high risk of fall | Lab | Computer vision system |
| 79) Nguyen T. O. et al. (2019) America [9] | N=60 | 51.8(21–80) | 63% | Normal Gait,  Abnormal Gait | Lab,  Clinical | Wearable device |
| 80) Buissière F. et al. (2020) Belgium [98] | N=73 | 83.0 ± 8.3 | 61.60% | Healthy, Fallers | Clinical | Wearable device |
| 81) Lincke A. et al. (2023) Sweden [169] | N=49 | ≥65 | NR | Healthy | Lab,  Clinical | Multimodal data acquisition devices |
| 82) Alves S.A. et al. (2024) Germany [90] | N=857 | 83.3± 8.7 | 71.76% | Healthy,  locomotor capacity impaired (Fallers, Using walking aids) | Clinical | AI-driven health platform |
| 83) Lusi A. et al. (2024) China [76] | N=240 | 60–89 | 60.83% | Healthy,  locomotor capacity impaired (squat ability limited) | Lab,  Home | AI-driven health platform |
| 84) Ming A., Clemens, V. et al. (2024) Germany [166] | N=329 | 67(59-72) | 38% | Healthy,  locomotor capacity impaired (PNP) | Clinical | Multimodal data acquisition devices |
| 85) Ming A., Schubert, T. et al. (2024) Germany [167] | N=152 | 65.0 ±10.5 | 37.50% | Healthy, Fallers | Clinical | Multimodal data acquisition devices |
| 86) Seifer A. K. et al. (2024) Germany [100] | N=48 | Young: 30.6(18–59),  Elderly: 70.6(60–83) | 56.25% | Healthy (Young, Elderly) | Lab | Wearable device |
| 87) Wipperman M. T. et al. (2024) America [101] | N=79 | OA: 62.75 (52–77)  Controls: 39 (19–85) | OA: 65%  Controls: 50% | Healthy,  locomotor capacity impaired (OA) | Lab | Wearable device |
| 88) Yan F. et al. (2024) China [102] | N=18 | Healthy: 45(30-60)  PD: 65(50-80) | 33.33% | Healthy,  locomotor capacity impaired (Mild PD, Severe PD) | Lab | Wearable device |
| 89) Cai J. et al. (2025) China [103] | N=163 | 82.6± 6.2 | 72.40% | Healthy,  locomotor capacity impaired (Fallers, Frailty Index) | Clinical | Wearable device |
| 90) Kim J. H. et al. (2024) Korea [56] | N=385 | Stroke patients:  65±12.2 (40–79)  Healthy:  66.7±7.3 (60–79) | 56.88% | Healthy,  locomotor capacity impaired (Stroke) | Lab,  Clinical | Computer vision system |
| 91) Kong H. H. et al. (2025) Korea [170] | N=83 | 71.6± 4.7 (65–85) | 83.10% | Healthy,  locomotor capacity impaired(K-FRAIL) | Community | Multimodal data acquisition devices |

Table 2 (Continued).

| 3. The domain of IC: Vitality (9) | | | | | | |
| --- | --- | --- | --- | --- | --- | --- |
| **Number) Author (Year) Country [Ref]** | **Number of participants** | **Mean age (Range age)** | **Female %** | **Participants’ IC condition** | **Setting** | **Type of AI-enabled digital devices** |
| 92) Wu L.-W. et al. (2022) China [117] | N=133 | 71.01 ± 4.14 | 59.40% | Healthy, Dynapenia, Pre-sarcopenia, Sarcopenia | Clinical | Fixed sensors device |
| 93) Di Martino F. et al. (2023) Italy [174] | N=42 | 65-95 | 77% | Healthy, At risk, Malnutrition | Clinical | Multimodal data acquisition devices |
| 94) Kim S. et al. (2023) Korea [171] | N=83 | Sarcopenia: 77.8 ±8.6,  Control: 63.4 ±15.4 | 53.01% | Healthy, Sarcopenia | Lab,  Clinical | Multimodal data acquisition devices |
| 95) Han S. et al. (2024) China [114] | N=51 | Controls 69.38 ± 5.48,  Sarcopenia 72.63 ± 5.35 | NR | Healthy, Sarcopenia | Lab | Wearable device |
| 96) Lin N. et al. (2024) China [93] | N=619 | 54.64 ±14.57 | 38.45% | Healthy, At risk of malnutrition  (NSR2002, PG-SGA SF) | Clinical | AI-driven health platform |
| 97) Naseem M. T. et al. (2024) Korea [172] | N=100 | 55(30-70+) | 77% | Healthy, Sarcopenia | Lab | Multimodal data acquisition devices |
| 98) Wang X et al. (2024) China [61] | N=482 | Normal: 56 ±14.4,  Malnutrition: 57 ±15.2 | 34.02% | Healthy, Malnutrition | Clinical | Computer vision system |
| 99) Wang X et al. (2024) China [173] | N=482 | 56.7 ± 10.1(18-80) | 34.02% | Healthy, Malnutrition | Clinical | Computer vision system |
| 100) Ferreira T. J. et al. (2025) Brazil [92] | N=1273 | 35 (18–65) | 54.60% | Normal-BMI,  Overweight,  Obese | Lab | AI-driven health platform |

Table 2 (Continued).

| 4. The domain of IC: Vision (16) | | | | | | |
| --- | --- | --- | --- | --- | --- | --- |
| **Number) Author (Year) Country [Ref]** | **Number of participants** | **Mean age (Range age)** | **Female %** | **Participants’ IC condition** | **Setting** | **Type of AI-enabled digital devices** |
| 101) Abràmoff M. D. et al. (2010) Netherlands [36] | N=16,670 | 71 ± 7.4 | 51.2% | Healthy, Visual capacity impaired (Minimal DR, more than minimal DR) | Community | Computer vision system |
| 102) Fleming A. D. et al. (2010) UK (Scotland) [37] | N=33,535 | 62 | 45.4% | Healthy, Visual capacity impaired (DR) | Community | Computer vision system |
| 103) Ting D. S. W. et al. (2017) Singapore [38] | N=14,880 | 60.2± 2.2 | 45.40% | Diabetes (with/without diabetic retinopathy) | Community | Computer vision system |
| 104) Tufail A. et al. (2017) Britain [39] | N=20,258 | 10–98 | NR | Healthy, Visual capacity impaired (DR) | Community,  Clinical | Computer vision system |
| 105) Abràmoff M. D. et al. (2018) America [40] | N=900 | 22–84 | 52.5% | Visual capacity impaired (DR, DME) | Community,  Clinical | Computer vision system |
| 106) Kanagasingam Y. et al. (2018) Australia [41] | N=193 | 55 (18–87) | 48% | Visual capacity impaired (Mild NPDR, Moderate NPDR, Severe NPDR) | Community,  Clinical | Computer vision system |
| 107) Keel S. et al. (2018) Australia [42] | N=96 | 44.26 ± 16.56 (20–90) | 42.71% | Visual capacity impaired (DR, Vision< 6/12) | Community,  Clinical | Computer vision system |
| 108) van der Heijden A. et al. (2018) Netherlands [43] | N=708 | 65 ± 11.9 | 43.90% | Healthy, Visual capacity impaired (DR) | Clinical | Computer vision system |
| 109) Verbraak F. D. et al. (2019) Netherlands [44] | N=1,616 | 63 ± 11.3 | 47.00% | Visual capacity impaired (DR, DME) | Community,  Clinical | Computer vision system |
| 110) Du et al. (2021) China [45] | N=8,948 | 50.04 ±11.98 | 65.39% | Visual capacity impaired (DR) | Community,  Clinical | Computer vision system |
| 111) Vasan C. S. et al. (2023) India [85] | N=1,407 | 60.8±8.4(40–90) | 59.60% | Visual capacity impaired (visual acuity <20/40) | Clinical | AI-driven health platform |
| 112) Jaworski D. et al. (2024) Poland [87] | N=453 | 1.Experiment 1:NR;  2.Experiment 2:  Glaucoma:73.18 ± 0.75  Control:71.28 ± 0.39  3.the expanded study:  ≥60 | NR | Healthy, Visual capacity impaired (Glaucoma) | Lab,  Clinical | AI-driven health platform |
| 113) Zhang R. et al. (2024) China [46] | N=1,904,927 | age groups reported:  ≤29, 30-39, 40-49,  50-59, ≥60 years | 48.50% | Healthy,  Visual capacity impaired (Ten retinal and optic nerve diseases) | Community,  Clinical | Computer vision system |
| 114) Zhu A. et al. (2024) America [47] | N=195 | 52.43±14.5 | 40% | Healthy,  Visual capacity impaired (AMD, DR, Glaucoma suspect) | Community | Computer vision system |
| 115) Armadá-Maresca F. et al. (2025) Spain [175] | N=2,478 | 58.5 ± 14.5 (18–96) | 61.90% | Healthy, Visual capacity impaired (General ophthalmology referrals) | Community,  Clinical | Multimodal data acquisition devices |
| 116) Senthil S. et al. (2025) India [86] | N=213 | 55 ± 14.7(18–88) | 40.18% | Healthy,  Visual capacity impaired (Definite glaucoma, Disc suspect) | Community,  Clinical | AI-driven health platform |

Table 2 (Continued).

| 5. The domain of IC: Hearing (8) | | | | | | |
| --- | --- | --- | --- | --- | --- | --- |
| **Number) Author (Year) Country [Ref]** | **Number of participants** | **Mean age (Range age)** | **Female %** | **Participants’ IC condition** | **Setting** | **Type of AI-enabled digital devices** |
| 117) Song X. D. et al. (2015) America [138] | N=21 | 47(18–90) | 61.90% | Healthy,  hearing-impaired | Lab | AI audiometer |
| 118) Barbour DL et al. (2019) America [88] | N=21 | 41±21 (19–79) | 61.90% | Health,  Mild hearing loss | Lab | AI-driven health platforms |
| 119) Zhang M. et al. (2019) China [140] | N=1,793 | ≥60 | 51.48% | Healthy,  Hearing Loss (Mild, Moderate, Severe, Profound) | Community | AI audiometer |
| 120) Charih F. et al. (2020) Canada [141] | N=270 | 39 ± 21 (12–85) | NR | Healthy,  Hearing Loss | Community | AI audiometer |
| 121) Heisey K. L. et al. (2020) America [139] | N=29 | 43(21–83) | 69% | Healthy, Hearing Loss | Lab,  Clinical | AI audiometer |
| 122) Kaszyński M. et al. (2024) Poland [142] | N=9,663 | 18–98 | 57.86% | Healthy, Hearing Loss | Lab,  Clinical | AI audiometer |
| 123) Wallaert N. et al. (2024) France [143] | N=43 | 55.3 ± 12.03 (18–88) | 51.62% | Healthy, Hearing Loss | Lab,  Clinical | AI audiometer |
| 124) Vercammen C. et al. (2025) Belgium [89] | N=408 | 18–95 | NR | Healthy,  hearing-impaired (At risk, Hearing Loss) | Home,  Online | AI-driven health platforms |

Table 2 (Continued).

| 6. The domain of IC: Psychological capacity (28) | | | | | | |
| --- | --- | --- | --- | --- | --- | --- |
| **Number) Author (Year) Country [Ref]** | **Number of participants** | **Mean age (Range age)** | **Female %** | **Participants’ IC condition** | **Setting** | **Type of AI-enabled digital devices** |
| 125) Jacobson N.C. et al. (2019) America [106] | N=55 | Patients: 42.8 ± 11.0,  Controls: 38.2 ± 13.0 | 40% | Healthy, Depression (MDD, Bipolar I/II) | Clinical | Wearable device |
| 126) Kim H. et al. (2019) Korea [177] | N=47 | 78±5.24 (65–94) | 85.10% | Health, Depression | Home | Multimodal data acquisition devices |
| 127) Minaeva O. et al. (2020) Netherlands [178] | N=179 | 20-65 | 64.20% | Healthy, Depression  (IDS-SR, BDI-II) | Home | Multimodal data acquisition devices |
| 128) Pedrelli P. et al. (2020) America [179] | N=31 | 33.7±14 (19–73) | 74% | MDD | Home | Multimodal data acquisition devices |
| 129) Smith M. et al. (2020) America [136] | N=46 | 81 (66–93) | 78.26% | Healthy, Depression (PHQ-9) | Lab,  Home | Speech analysis system |
| 130) Badal V. D. et al. (2021) America [129] | N=97 | 66-94 | 64.95% | Healthy,  Depression (PHQ-9) | Lab,  Community | Speech analysis system |
| 131) Little B. et al. (2020) Britain [183] | N=58 | LLD: 74.2±6.0  Controls: 74.0±5.9 | 74.14% | Healthy, LLD | Home | Multimodal data acquisition devices |
| 132) Mendez J. I. et al. (2021) Mexico [124] | N=7,882 | 50.73±17.42 (20-80) | 51% | Healthy,  Depression (PHQ-9) | Home | Smart home |
| 133) Nickels S. et al. (2021) America [176] | N=384 | 18–79 | 87.50% | Healthy,  Depression (PHQ-9) | Home,  Online | Multimodal data acquisition devices |
| 134) Opoku Asare K. et al. (2021) Finland [91] | N=629 | 18–65+ | 11.00% | Healthy,  Depression (PHQ-9) | Home,  Online | AI-driven health platforms |
| 135) Rykov Y. et al. (2021) Singapore [107] | N=267 | 33 ± 8.6 (21-64) | 63.70% | Healthy, Depression (PHQ-9) | Home,  Community | Wearable device |
| 136) Asare, K. O. et al. (2022) Finland [180] | N=54 | 43.04 ± 11.58 (24–68) | 55.56% | Healthy, Depression (DASS-21) | Home,  Community | Multimodal data acquisition devices |
| 137) Choi J. et al. (2022) Korea [111] | N=14 | 76 (65–86) | 85.70% | Healthy, Depression (with past depression diagnosis) | Home,  Community | Wearable device |
| 138) Jung D. et al. (2022) Korea [99] | N=45 | ≥65 | 60% | Healthy, MDD | Community | Wearable device |
| 139) Lin D. et al. (2022) America [80] | N=263 | 63±10.3 (22–80) | 48.08% | Healthy, Depression | Home,  Online | AI-driven health platforms |
| 140) Kaywan P. et al. (2023) Australia [81] | N=50 | 34.7(18–80) | 45% | Healthy, Depressive (IDS-SR) | Home,  Online | AI-driven health platforms |
| 141) Ye J. et al. (2023) China [62] | N=164 | 18–65 | NR | Healthy, Depression (HAMD) | Lab | Computer vision system |
| 142) Zhou Y. et al. (2023) China [184] | N=319 | 70.1 ± 6.9 | 60.50% | MCI(n=319),  subdivided into Normal, Depressed, Anxious, Apathetic, and Comorbid groups | Clinical | Multimodal data acquisition devices |
| 143) Lee T. R. et al. (2024) Korea [112] | N=352 | 72.48±5.9 (60-90) | 73.01% | Healthy,  Depression only,  Anxiety only,  Both depression & anxiety | Home | Wearable device |
| 144) Rykov Y. et al. (2024) Singapore [113] | N =16 | 57.8±4.6 | 62.50% | Healthy,  Depression (DASS, MBIC) | Home,  Community | Wearable device |
| 145) Song S. et al. (2024) Korea [181] | N=25 | 76.4±4.23 (65–84) | 76% | Healthy, Depression (Self-reported medical history) | Home,  Community | Multimodal data acquisition devices |
| 146) Chung M.K. et al. (2025) Korea [77] | N=230 | ≥55 | NR | Healthy, Depression (PHQ-9) | Community,  Lab | AI-driven health platforms |
| 147) Lin S. et al. (2025) China [50] | N=50 | 62.9 ± 1.3 | 68% | Healthy, Depression (CESD-10) | Home,  Online | Computer vision system |
| 148) Liu Y. et al. (2025) China [185] | N=146 | 18-65 | NR | Healthy, Depression (HAMD-24) | Community,  Lab | Multimodal data acquisition devices |
| 149) Mazur A. et al. (2025) America, Canada [137] | N=14,898 | 37.3±14.3 (18–93) | 69.50% | Healthy, Depression (PHQ-9) | Clinical | Speech analysis system |
| 150) Nejadshamsi S. et al. (2025) Canada [26] | N=4 | 65-70 | 50% | Healthy, Depression (GDS) | Home,  Online | Smart home |
| 151) Zhang Y. et al. (2025) Britain [182] | N=10,129 | 18-93 | 69.30% | Healthy, Depression (PHQ-8, GAD-7) | Lab,  Online | Multimodal data acquisition devices |
| 152) Kim D. et al. (2026) South Korea [82] | N=2896 | 77.93±7.04 | 68% | low risk (GDS-SF < 2)；high risk of depressive symptoms (GDS-SF ≥ 2) | Online | AI-driven health platform |

Table 2 (Continued).

| 7. The domain of IC: multiple IC domains (9) | | | | | | |
| --- | --- | --- | --- | --- | --- | --- |
| **Number) Author (Year) Country [Ref]** | **Number of participants** | **Mean age**  **(Range age)** | **Female %** | **Participants’ IC condition** | **Setting** | **Type of AI-enabled digital devices** |
| 153) Jung D. et al. (2020) Korea [29] | N=54 | ≥65 | NR | Healthy, CI,At locomotor capacity and vitality impaired risk ;  Robust, Non-robust (FRAIL) | Lab | Wearable device |
| 154) Arshad M. Z. et al. (2021) Korea [28] | N=71 | 77.56 (71–86) | 61.98% | Healthy, At locomotor capacity and vitality impaired risk;  Robust, Non-robust (FRAIL) | Clinical | Wearable device |
| 155) Jung D. et al. (2021) Korea [29] | N=74 | Robust: 76 ± 4,  Pre-frail: 78 ± 4,  Frail: 79 ± 3 | 64.89% | Healthy, At locomotor capacity and vitality impaired risk;  Healthy, Pre-frail, Frail (FRAIL) | Lab,  Clinical | Wearable device |
| 156) Park C. et al. (2021) America [30] | N=259 | 76.0 ± 9.8 | 64.90% | Healthy, At locomotor capacity and vitality impaired risk; Healthy, Pre-frail, Frail (FFP) | Community | Wearable device |
| 157) Schütz N. et al. (2022) Korea [31] | N=45 | 87±7 | 67% | Healthy, Fall-risk (TUG, POMA), Frailty (EFS), Depression (GDS), MCI(MoCA) | Lab,  Home | Smart home |
| 158) Liu Y. et al. (2023) China [32] | N=45 | 65-80 | NR | Older adults in community rehabilitation settings | Community | Multimodal data acquisition devices |
| 159) Aznar-Grimón R. et al. (2024) Spain [33] | N=42 | 70.5(66-81) | 57.14% | Healthy, Sarcopenia, Cognitive Decline | Lab,  Clinical | Multimodal data acquisition devices |
| 160) Kolakowski M. et al. (2025) Poland [34] | N=64 | ≥60 | NR | Healthy, IC decline | Community,  Home | Multimodal data acquisition devices |
| 161) Tavassoli et al. (2022) France [35] | N=10,903 | 76 (≥60) | 61% | Healthy, IC decline | Home | Multimodal data acquisition devices |

- N: Number; IC: Intrinsic capacity; NR: Not reported; MCI: Mild cognitive impairment; CI: Cognitive Impairment; AD: Alzheimer’s disease; SCD: Subjective Cognitive Decline; aMCI: amnestic mild cognitive impairment;
- HD: Huntington’s disease; PS: Post stroke; FRAIL: Fatigue, Resistance, Ambulation, Illnesses, and Loss of weight scale; TUG: Timed Up and Go; PNP Assessment: Peripheral Neuropathy Assessment; OA: Osteoarthritis; PD: Parkinson’s Disease; K-FRAIL: Korean-FRAIL;
- NRS 2002: Nutrition Risk Screening 2002; PG-SGA SF: Patient-Generated Subjective Global Assessment Short Form;
- DR: Diabetic Retinopathy; DME: Diabetic Macular Edema; NPDR: non-proliferative diabetic retinopathy; AMD: Age-related macular degeneration;
- MDD: Major Depressive Disorder; Bipolar I/II: Bipolar Disorder I/II; IDS-SR: Inventory of Depressive Symptomatology - Self-Report; BDI-II: Beck Depression Inventory - Second Edition; LLD: late-life depression; PHQ-9: Patient Health Questionnaire-9 item version;DASS-21: Depression Anxiety Stress Scales- 21 item version;MBIC: Mild Behavioral Impairment Checklist;CESD-10: Center for Epidemiologic Studies Depression Scale-10;HAMD-24: Hamilton Depression Rating Scale – 24 items; GDS: GDS: Global Deterioration Scale; PHQ-8: Patient Health Questionnaire-8 items; GAD-7: Generalized Anxiety Disorder 7-item scale;
- FFP: Fried Frailty Phenotype; POMA: Performance-Oriented Mobility Assessment; EFS: Edmonton Frail Scale MoCA: Montreal Cognitive Assessment Scale.

**References**

**(26 -186)**

26. Nejadshamsi S, Karami V, Ghourchian N, et al. Development and feasibility study of HOPE model for prediction of depression among older adults using Wi-Fi-based motion sensor data: machine learning study. JMIR Aging. Mar 3, 2025;8:e67715. [doi: 10.2196/67715] [Medline: 40053734]

27. Jung D, Nguyen MD, Park M, et al. Walking-in-place characteristics-based geriatric assessment using deep convolutional neural networks. Annu Int Conf IEEE Eng Med Biol Soc. Jul 2020;2020:3931-3935. [doi: 10.1109/EMBC44109.2020.9176069] [Medline: 33018860]

28. Arshad MZ, Jung D, Park M, Shin H, Kim J, Mun KR. Gait-based frailty assessment using image representation of IMU signals and deep CNN. Presented at: 2021 43rd Annual International Conference of the IEEE Engineering in Medicine & Biology Society (EMBC); Nov 2021:1874-1879; Mexico. Nov 2021.[doi: 10.1109/EMBC46164.2021.9630976] [Medline: 34891653]

29. Jung D, Kim J, Kim M, Won CW, Mun KR. Frailty assessment using temporal gait characteristics and a long short-term memory network. IEEE J Biomed Health Inform. Sep 2021;25(9):3649-3658. [doi: 10.1109/JBHI.2021.3067931] [Medline: 33755570]

30. Park C, Mishra R, Golledge J, Najafi B. Digital biomarkers of physical frailty and frailty phenotypes using sensor-based physical activity and machine learning. Sensors. Aug 5, 2021;21(16):5289. [doi: 10.3390/s21165289]

31. Schütz N, Knobel SEJ, Botros A, et al. A systems approach towards remote health-monitoring in older adults: introducing a zero-interaction digital exhaust. NPJ Digit Med. Aug 16, 2022;5(1):116. [doi: 10.1038/s41746-022-00657-y] [Medline: 35974156]

32. Liu Y, Liu T, Zhao C, Wang X. Research and application of digital rehabilitation evaluation methods for elderly care communities. China Digit Med. 2023;18(5):110-116. [doi: 10.3969/j.issn.1673-7571.2023.05.022]

33. Aznar-Gimeno R, Perez-Lasierra JL, Pérez-Lázaro P, et al. Gait-based AI models for detecting sarcopenia and cognitive decline using sensor fusion. Diagnostics (Basel). Dec 22, 2024;14(24):2886. [doi: 10.3390/diagnostics14242886] [Medline: 39767247]

34. Kolakowski M, Lupica A, Ben Bader S, et al. CAREUP: an integrated care platform with intrinsic capacity monitoring and prediction capabilities. Sensors (Basel). Feb 3, 2025;25(3):916. [doi: 10.3390/s25030916] [Medline: 39943555]

35. Tavassoli N, de Souto Barreto P, Berbon C, et al. Implementation of the WHO integrated care for older people (ICOPE) programme in clinical practice: a prospective study. Lancet Healthy Longev. Jun 2022;3(6):e394-e404. [doi: 10.1016/S2666-7568(22)00097-6] [Medline: 36098317]

36. Abràmoff MD, Reinhardt JM, Russell SR, et al. Automated early detection of diabetic retinopathy. Ophthalmology. Jun 2010;117(6):1147-1154. [doi: 10.1016/j.ophtha.2010.03.046] [Medline: 20399502]

37. Fleming AD, Goatman KA, Philip S, Prescott GJ, Sharp PF, Olson JA. Automated grading for diabetic retinopathy: a large-scale audit using arbitration by clinical experts. Br J Ophthalmol. Dec 2010;94(12):1606-1610. [doi: 10.1136/bjo.2009.176784] [Medline: 20858722]

38. Ting DSW, Cheung CYL, Lim G, et al. Development and validation of a deep learning system for diabetic retinopathy and related eye diseases using retinal images from multiethnic populations with diabetes. JAMA. Dec 12, 2017;318(22):2211-2223. [doi: 10.1001/jama.2017.18152] [Medline: 29234807]

39. Tufail A, Rudisill C, Egan C, et al. Automated diabetic retinopathy image assessment software: diagnostic accuracy and cost-effectiveness compared with human graders. Ophthalmology. Mar 2017;124(3):343-351. [doi: 10.1016/j.ophtha.2016.11.014] [Medline: 28024825]

40. Abràmoff MD, Lavin PT, Birch M, Shah N, Folk JC. Pivotal trial of an autonomous AI-based diagnostic system for detection of diabetic retinopathy in primary care offices. NPJ Digit Med. 2018;1(39):39. [doi: 10.1038/s41746-018-0040-6] [Medline: 31304320]

41. Kanagasingam Y, Xiao D, Vignarajan J, Preetham A, Tay-Kearney ML, Mehrotra A. Evaluation of artificial intelligence-based grading of diabetic retinopathy in primary care. JAMA Netw Open. Sep 7, 2018;1(5):e182665. [doi: 10.1001/jamanetworkopen.2018.2665] [Medline: 30646178]

42. Keel S, Lee PY, Scheetz J, et al. Feasibility and patient acceptability of a novel artificial intelligence-based screening model for diabetic retinopathy at endocrinology outpatient services: a pilot study. Sci Rep. Mar 12, 2018;8(1):4330. [doi: 10.1038/s41598-018-22612-2] [Medline: 29531299]

43. van der Heijden AA, Abramoff MD, Verbraak F, van Hecke MV, Liem A, Nijpels G. Validation of automated screening for referable diabetic retinopathy with the IDx-DR device in the Hoorn Diabetes Care System. Acta Ophthalmol. Feb 2018;96(1):63-68. [doi: 10.1111/aos.13613] [Medline: 29178249]

44. Verbraak FD, Abramoff MD, Bausch GCF, et al. Diagnostic accuracy of a device for the automated detection of diabetic retinopathy in a primary care setting. Diabetes Care. Apr 2019;42(4):651-656. [doi: 10.2337/dc18-0148] [Medline: 30765436]

45. Du Z, Liu J, Hu H, Yang N, Wen L, Wang F, et al. Evaluation of deep learning network model based on attention mechanism in diabetic retinopathy screening in China. Chin J Diabetes Mellitus. 2021;13(12):1148-1154. [doi: 10.3760/cma.j.cn115791-20210314-00152]

46. Zhang R, Dong L, Fu X, et al. Trends in the prevalence of common retinal and optic nerve diseases in China: an artificial intelligence based national screening. Trans Vis Sci Tech. Apr 2, 2024;13(4):28. [doi: 10.1167/tvst.13.4.28]

47. Zhu A, Tailor P, Verma R, et al. Implementation of deep learning artificial intelligence in vision-threatening disease screenings for an underserved community during COVID-19. J Telemed Telecare. Dec 2024;30(10):1590-1597. [doi: 10.1177/1357633X231158832] [Medline: 36908254]

48. Seifallahi M, Mehraban AH, Galvin JE, Ghoraani B. Alzheimer’s disease detection using comprehensive analysis of timed up and go test via Kinect V.2 Camera and machine learning. IEEE Trans Neural Syst Rehabil Eng. 2022;30(1589-600):1589-1600. [doi: 10.1109/TNSRE.2022.3181252] [Medline: 35675251]

49. Dubois A, Bihl T, Bresciani JP. Automating the timed up and go test using a depth camera. Sensors (Basel). Dec 22, 2017;18(1):14. [doi: 10.3390/s18010014] [Medline: 29271926]

50. Lin S, Li S, Fang Y. Interpretable machine learning for depression recognition with spatiotemporal gait features among older adults: a cross-sectional study in Xiamen, China. BMC Geriatr. Jul 2, 2025;25(1):453. [doi: 10.1186/s12877-025-06101-6] [Medline: 40604502]

51. Kargar BAH, Mollahosseini A, Struemph T, Pace W, Nielsen RD, Mahoor MH. Automatic measurement of physical mobility in Get-Up-and-Go Test using Kinect sensor. Annu Int Conf IEEE Eng Med Biol Soc. 2014;2014(3492-5):3492-3495. [doi: 10.1109/EMBC.2014.6944375] [Medline: 25570743]

52. Farrell B, Horn J, Seifallahi M, Galvin JE, Ghoraani B. Mild cognitive impairment detection through gait analysis and standard cameras. Annu Int Conf IEEE Eng Med Biol Soc. Jul 2024;2024:1-4. [doi: 10.1109/EMBC53108.2024.10782745] [Medline: 40039723]

53. Matsuura T, Sakashita K, Grushnikov A, Okura F, Mitsugami I, Yagi Y. Statistical Analysis of Dual-task Gait Characteristics for Cognitive Score Estimation. Sci Rep. Dec 27, 2019;9(1):31882727. [doi: 10.1038/s41598-019-56485-w]

54. Satake Y, Taomoto D, Wu S, et al. The clinical significance of an AI-based assumption model for neurocognitive diseases using a novel dual-task system. Sci Rep. Summer 2025;15(1):40263361. [doi: 10.1038/s41598-025-98319-y]

55. Altilio R, Paoloni M, Panella M. Selection of clinical features for pattern recognition applied to gait analysis. Med Biol Eng Comput. Apr 2017;55(4):685-695. [doi: 10.1007/s11517-016-1546-1] [Medline: 27435068]

56. Kim JH, Hong H, Lee K, et al. AI in evaluating ambulation of stroke patients: severity classification with video and functional ambulation category scale. Top Stroke Rehabil. Jan 2, 2025;32(1):34-42. [doi: 10.1080/10749357.2024.2359342]

57. Mengoudi K, Ravi D, Yong KXX, et al. Augmenting dementia cognitive assessment with instruction-less eye-tracking tests. IEEE J Biomed Health Inform. Nov 2020;24(11):3066-3075. [doi: 10.1109/JBHI.2020.3004686] [Medline: 32749977]

58. Sun J, Liu Y, Wu H, Jing P, Ji Y. A novel deep learning approach for diagnosing Alzheimer’s disease based on eye-tracking data. Front Hum Neurosci. 2022;16:972773. [doi: 10.3389/fnhum.2022.972773] [Medline: 36158627]

59. Zuo F, Jing P, Sun J, Duan J, Ji Y, Liu Y. Deep learning-based eye-tracking analysis for diagnosis of Alzheimer’s disease using 3D comprehensive visual stimuli. IEEE J Biomed Health Inform. May 2024;28(5):2781-2793. [doi: 10.1109/JBHI.2024.3365172] [Medline: 38349825]

60. Umeda-Kameyama Y, Kameyama M, Tanaka T, et al. Screening of Alzheimer’s disease by facial complexion using artificial intelligence. Aging (Albany NY). Jan 25, 2021;13(2):1765-1772. [doi: 10.18632/aging.202545] [Medline: 33495415]

61. Wang X, Liu Y, Rong Z, et al. Development and evaluation of a deep learning framework for the diagnosis of malnutrition using a 3D facial points cloud: a cross‐sectional study. J Parenter Enteral Nutr. Jul 2024;48(5):554-561. [doi: 10.1002/jpen.2643]

62. Ye J, Yu Y, Fu G, et al. Analysis and recognition of voluntary facial expression mimicry based on depressed patients. IEEE J Biomed Health Inform. Aug 2023;27(8):3698-3709. [doi: 10.1109/JBHI.2023.3260816] [Medline: 37030686]

63. Amini S, Zhang L, Hao B, et al. An artificial intelligence-assisted method for dementia detection using images from the clock drawing test. J Alzheimers Dis. 2021;83(2):581-589. [doi: 10.3233/JAD-210299] [Medline: 34334396]

64. Najafi B, Lee M, Dehghan Rouzi M, Runyon JR, Sternberg EM, LaFleur BJ. A 20-s video-based assessment of cognitive frailty: results from a cohort study within the precision aging network. Gerontology. 2025;71(7):589-600. [doi: 10.1159/000546227] [Medline: 40359928]

65. Dubois A, Mouthon A, Sivagnanaselvam RS, Bresciani JP. Fast and automatic assessment of fall risk by coupling machine learning algorithms with a depth camera to monitor simple balance tasks. J Neuroeng Rehabil. Jun 11, 2019;16(1):71. [doi: 10.1186/s12984-019-0532-x] [Medline: 31186002]

66. Dahmen J, Cook D, Fellows R, Schmitter-Edgecombe M. An analysis of a digital variant of the Trail Making Test using machine learning techniques. Technol Health Care. 2017;25(2):251-264. [doi: 10.3233/THC-161274] [Medline: 27886019]

67. Bergeron MF, Landset S, Tarpin-Bernard F, Ashford CB, Khoshgoftaar TM, Ashford JW. Episodic-memory performance in machine learning modeling for predicting cognitive health status classification. J Alzheimers Dis. 2019;70(1):277-286. [doi: 10.3233/JAD-190165] [Medline: 31177223]

68. Jia Z, Dong M, Shi Z, Jin C, Li G. Study of a screening system for mild cognitive impairment based on machine learning model. J Shanghai Jiao Tong Univ Med Sci. 2019;39(8):908-913. [doi: 10.3969/j.issn.1674-8115.2019.08.017]

69. Rutkowski TM, Abe MS, Koculak M, Otake-Matsuura M. Classifying mild cognitive impairment from behavioral responses in emotional arousal and valence evaluation task - AI approach for early dementia biomarker in aging societies. Annu Int Conf IEEE Eng Med Biol Soc. Jul 2020;2020:5537-5543. [doi: 10.1109/EMBC44109.2020.9175805] [Medline: 33019233]

70. Zhang Y, Chen Y, Yu H, et al. What can “drag & drop” tell? Detecting mild cognitive impairment by hand motor function assessment under dual-task paradigm. Int J Hum Comput Stud. Jan 2021;145:102547. [doi: 10.1016/j.ijhcs.2020.102547]

71. Modarres MH, Kalafatis C, Apostolou P, Tabet N, Khaligh-Razavi SM. The use of the integrated cognitive assessment to improve the efficiency of primary care referrals to memory services in the accelerating dementia pathway technologies study. Front Aging Neurosci. 2023;15:1243316. [doi: 10.3389/fnagi.2023.1243316] [Medline: 37781102]

72. Leng F, Xu G, Long R, Xu W, Wang T, et al. Clinical application of an intelligently modified mini-cog assessment tool for screening cognitive impairment. Chin J Geriatr Cardiovasc Cerebrovasc Dis. 2023;25(8):793-796. [doi: 10.3969/j.issn.1009-0126.2023.08.003]

73. Segev O, Raz I, Gerstein HC, et al. Development and first-stage validation of a digital version of the Digit Symbol Substitution test for use in assessing cognitive function in older people with diabetes. Diabetes Obes Metab. Aug 2024;26(8):3299-3305. [doi: 10.1111/dom.15657] [Medline: 38757537]

74. Huang G, Li R, Roccati E, et al. Feasibility of computerized motor, cognitive and speech tests in the home: analysis of TAS Test in 2,300 older adults. J Prev Alzheimers Dis. Apr 2025;12(4):100081. [doi: 10.1016/j.tjpad.2025.100081] [Medline: 40000322]

75. Liew TM, Foo JYH, Yang H, et al. PENSIEVE-AI a brief cognitive test to detect cognitive impairment across diverse literacy. Nat Commun. Mar 23, 2025;16(1):2847. [doi: 10.1038/s41467-025-58201-x] [Medline: 40122854]

76. Lusi A, Li T, Yu J, Zhang C. Automatic analysis pipeline of squatting ability for elderly people based on an intelligent platform. Annu Int Conf IEEE Eng Med Biol Soc. Jul 2024;2024:1-6. [doi: 10.1109/EMBC53108.2024.10781505] [Medline: 40039293]

77. Chung MK, Lee SY, Shin T, et al. BERT and BERTopic for screening clinical depression on open-ended text messages collected through a mobile application from older adults. BMC Public Health. Jun 10, 2025;25(1):2161. [doi: 10.1186/s12889-025-23337-4] [Medline: 40495126]

78. Tang F, Uchendu I, Wang F, Dodge HH, Zhou J. Scalable diagnostic screening of mild cognitive impairment using AI dialogue agent. Sci Rep. Mar 31, 2020;10(1):5732. [doi: 10.1038/s41598-020-61994-0] [Medline: 32235884]

79. Fristed E, Skirrow C, Meszaros M, et al. A remote speech-based AI system to screen for early Alzheimer’s disease via smartphones. Alzheimers Dement (Amst). 2022;14(1):e12366. [doi: 10.1002/dad2.12366] [Medline: 36348974]

80. Lin D, Nazreen T, Rutowski T, et al. Feasibility of a machine learning-based smartphone application in detecting depression and anxiety in a generally senior population. Front Psychol. 2022;13(811517):811517. [doi: 10.3389/fpsyg.2022.811517] [Medline: 35478769]

81. Kaywan P, Ahmed K, Ibaida A, Miao Y, Gu B. Early detection of depression using a conversational AI bot: a non-clinical trial. PLoS One. 2023;18(2):e0279743. [doi: 10.1371/journal.pone.0279743] [Medline: 36735701]

82. Kim D, Jang R, Jang SN, Park S. When a chatbot asks “How are you?”: A cross-sectional study of AI call conversations and depressive symptom detection among older adults in rural South Korea. BMC Geriatr. Jan 28, 2026;26(1):247. [doi: 10.1186/s12877-026-07038-0] [Medline: 41606503]

83. Haque RU, Pongos AL, Manzanares CM, Lah JJ, Levey AI, Clifford GD. Deep convolutional neural networks and transfer learning for measuring cognitive impairment using eye-tracking in a distributed tablet-based environment. IEEE Trans Biomed Eng. Jan 2021;68(1):11-18. [doi: 10.1109/TBME.2020.2990734] [Medline: 32340935]

84. Li Q, Yan J, Ye J, et al. Construction of a prediction model for Alzheimer’s disease using an AI-driven eye-tracking task on mobile devices. Aging Clin Exp Res. Dec 27, 2024;37(1):39725797. [doi: 10.1007/s40520-024-02882-9]

85. Vasan CS, Gupta S, Shekhar M, et al. Accuracy of an artificial intelligence-based mobile application for detecting cataracts: results from a field study. Indian J Ophthalmol (Poona City). 2023;71(8):2984-2989. [doi: 10.4103/IJO.IJO_3372_22]

86. Senthil S, Rao DP, Savoy FM, et al. Evaluating real-world performance of an automated offline glaucoma AI on a smartphone fundus camera across glaucoma severity stages. PLoS One. 2025;20(6):e0324883. [doi: 10.1371/journal.pone.0324883] [Medline: 40569941]

87. Jaworski D, Suwała K, Kaluzny BJ, Kaluzny JJ. Comparison of an AI-based mobile pupillometry system and NPi-200 for pupillary light reflex and correlation with glaucoma-related markers. Front Neurol. 2024;15:1426205. [doi: 10.3389/fneur.2024.1426205] [Medline: 39850730]

88. Barbour DL, Howard RT, Song XD, et al. Online machine learning audiometry. Ear Hear. 2019;40(4):918-926. [doi: 10.1097/AUD.0000000000000669] [Medline: 30358656]

89. Vercammen C, Strelcyk O. Development and validation of a self-administered online hearing test. Trends Hear. 2025;29:23312165251317923. [doi: 10.1177/23312165251317923] [Medline: 40101250]

90. Alves SA, Temme S, Motamedi S, et al. Evaluating the prognostic and clinical validity of the fall risk score derived from an AI-based mHealth app for fall prevention: retrospective real-world data analysis. JMIR Aging. Dec 4, 2024;7:e55681. [doi: 10.2196/55681] [Medline: 39631046]

91. Opoku Asare K, Terhorst Y, Vega J, Peltonen E, Lagerspetz E, Ferreira D. Predicting depression from smartphone behavioral markers using machine learning methods, hyperparameter optimization, and feature importance analysis: exploratory study. JMIR Mhealth Uhealth. Jul 12, 2021;9(7):e26540. [doi: 10.2196/26540] [Medline: 34255713]

92. Ferreira TJ, Salvador IC, Pessanha CR, et al. Advances in the estimation of body fat percentage using an artificial intelligence 2D-photo method. NPJ Digit Med. Jan 18, 2025;8(1):43. [doi: 10.1038/s41746-024-01380-6] [Medline: 39827323]

93. Lin N, Zhou X, Chen W, et al. Development and validation of a point-of-care nursing mobile tool to guide the diagnosis of malnutrition in hospitalized adult patients: a multicenter, prospective cohort study. MedComm (2020). Apr 2024;5(4):e526. [doi: 10.1002/mco2.526] [Medline: 38606361]

94. Obuchi SP, Kojima M, Suzuki H, et al. Artificial intelligence detection of cognitive impairment in older adults during walking. Alzheimers Dement (Amst). 2024;16(3):e70012. [doi: 10.1002/dad2.70012] [Medline: 39328904]

95. Mannini A, Trojaniello D, Cereatti A, Sabatini AM. A machine learning framework for gait classification using inertial sensors: application to elderly, post-stroke and Huntington’s disease patients. Sensors (Basel). Jan 21, 2016;16(1):26805847. [doi: 10.3390/s16010134] [Medline: 26805847]

96. Moon G, Cho J, Choi H, Kim Y, Kim GD, Jang SH. AI-based severity classification of dementia using gait analysis. Sensors (Basel). Oct 2, 2025;25(19):6083. [doi: 10.3390/s25196083] [Medline: 41094906]

97. Nguyen TQ, Young JH, Rodriguez A, Zupancic S, Lie DYC. Differentiation of patients with balance insufficiency (vestibular hypofunction) versus normal subjects using a low-cost small wireless wearable gait sensor. Biosensors (Basel). Feb 26, 2019;9(1):29. [doi: 10.3390/bios9010029] [Medline: 30813585]

98. Buisseret F, Catinus L, Grenard R, et al. Timed up and go and six-minute walking tests with wearable inertial sensor: one step further for the prediction of the risk of fall in elderly nursing home people. Sensors (Basel). Jun 5, 2020;20(11):3207. [doi: 10.3390/s20113207] [Medline: 32516995]

99. Jung D, Kim J, Mun KR. Identifying depression in the elderly using gait accelerometry. Annu Int Conf IEEE Eng Med Biol Soc. Jul 2022;2022(4946-9):4946-4949. [doi: 10.1109/EMBC48229.2022.9871877] [Medline: 36086152]

100. Seifer AK, Kuderle A, Dorschky E, Moradi H, Hannemann R, Eskofier BM. Step length and gait speed estimation using a hearing aid integrated accelerometer: a comparison of different algorithms. IEEE J Biomed Health Inform. Nov 2024;28(11):6619-6628. [doi: 10.1109/JBHI.2024.3454824] [Medline: 39236137]

101. Wipperman MF, Lin AZ, Gayvert KM, et al. Digital wearable insole-based identification of knee arthropathies and gait signatures using machine learning. Elife. Apr 30, 2024;13:e86132. [doi: 10.7554/eLife.86132] [Medline: 38686919]

102. Yan F, Gong J, Zhang Q, He H. Learning motion primitives for the quantification and diagnosis of mobility deficits. IEEE Trans Biomed Eng. Dec 2024;71(12):3339-3349. [doi: 10.1109/TBME.2024.3404357] [Medline: 38776195]

103. Cai J, Guan Z, Wang J, et al. Impact of gait parameters and their variability on fall risk assessment accuracy using wearable sensor. IEEE Trans Neural Syst Rehabil Eng. 2025;33(1996-2003):1996-2003. [doi: 10.1109/TNSRE.2025.3572109] [Medline: 40397645]

104. Ghosh S, Fleiner T, Giannouli E, et al. Statistical learning of mobility patterns from long-term monitoring of locomotor behaviour with body-worn sensors. Sci Rep. May 4, 2018;8(1):7079. [doi: 10.1038/s41598-018-25523-4] [Medline: 29728658]

105. Gramkow MH, Brink-Kjær A, Clemmensen FK, et al. Diagnostic performance of actigraphy in Alzheimer’s disease using a machine learning classifier – a cross-sectional memory clinic study. Alz Res Therapy. May 21, 2025;17(1):40399918. [doi: 10.1186/s13195-025-01751-5]

106. Jacobson NC, Weingarden H, Wilhelm S. Digital biomarkers of mood disorders and symptom change. NPJ Digit Med. 2019;2(3):3. [doi: 10.1038/s41746-019-0078-0] [Medline: 31304353]

107. Rykov Y, Thach TQ, Bojic I, Christopoulos G, Car J. Digital biomarkers for depression screening with wearable devices: cross-sectional study with machine learning modeling. JMIR Mhealth Uhealth. Oct 25, 2021;9(10):e24872. [doi: 10.2196/24872] [Medline: 34694233]

108. Yang D, Hong KS. Quantitative assessment of resting-state for mild cognitive impairment detection: a functional near-infrared spectroscopy and deep learning approach. J Alzheimers Dis. 2021;80(2):647-663. [doi: 10.3233/JAD-201163] [Medline: 33579839]

109. Ho TKK, Kim M, Jeon Y, et al. Deep learning-based multilevel classification of Alzheimer’s disease using non-invasive functional near-infrared spectroscopy. Front Aging Neurosci. 2022;14:810125. [doi: 10.3389/fnagi.2022.810125] [Medline: 35557842]

110. Pu Z, Huang H, Li M, et al. Screening tools for subjective cognitive decline and mild cognitive impairment based on task-state prefrontal functional connectivity: a functional near-infrared spectroscopy study. Neuroimage. Apr 15, 2025;310:121130. [doi: 10.1016/j.neuroimage.2025.121130] [Medline: 40058532]

111. Choi J, Lee S, Kim S, Kim D, Kim H. Depressed mood prediction of elderly people with a wearable band. Sensors. May 31, 2022;22(11):4174. [doi: 10.3390/s22114174]

112. Lee TR, Kim GH, Choi MT. Geriatric depression and anxiety screening via deep learning using activity tracking and sleep data. Int J Geriatr Psychiatry. Feb 2024;39(2):e6071. [doi: 10.1002/gps.6071] [Medline: 38372966]

113. Rykov YG, Ng KP, Patterson MD, Gangwar BA, Kandiah N. Predicting the severity of mood and neuropsychiatric symptoms from digital biomarkers using wearable physiological data and deep learning. Comput Biol Med. Sep 2024;180:108959. [doi: 10.1016/j.compbiomed.2024.108959] [Medline: 39089109]

114. Han S, Xiao Q, Liang Y, et al. Using flexible-printed piezoelectric sensor arrays to measure plantar pressure during walking for sarcopenia screening. Sensors (Basel). Aug 11, 2024;24(16):5189. [doi: 10.3390/s24165189] [Medline: 39204885]

115. Lai DTH, Begg RK, Taylor S, Palaniswami M. Detection of tripping gait patterns in the elderly using autoregressive features and support vector machines. J Biomech. 2008;41(8):1762-1772. [doi: 10.1016/j.jbiomech.2008.02.037] [Medline: 18433757]

116. Ballesteros J, Urdiales C, Martinez AB, Tirado M. Automatic assessment of a rollator-user’s condition during rehabilitation using the i-Walker platform. IEEE Trans Neural Syst Rehabil Eng. Nov 2017;25(11):2009-2017. [doi: 10.1109/TNSRE.2017.2698005] [Medline: 28459694]

117. Wu LW, OuYoung T, Chiu YC, Hsieh HF, Hsiu H. Discrimination between possible sarcopenia and metabolic syndrome using the arterial pulse spectrum and machine-learning analysis. Sci Rep. Dec 12, 2022;12(1):21452. [doi: 10.1038/s41598-022-26074-5] [Medline: 36509825]

118. Dawadi PN, Cook DJ, Schmitter-Edgecombe M. Automated cognitive health assessment using smart home monitoring of complex tasks. IEEE Trans Syst Man Cybern Syst. Nov 2013;43(6):1302-1313. [doi: 10.1109/TSMC.2013.2252338] [Medline: 25530925]

119. König A, Crispim-Junior CF, Covella AGU, et al. Ecological assessment of autonomy in instrumental activities of daily living in dementia patients by the means of an automatic video monitoring system. Front Aging Neurosci. 2015;7(JUN):98. [doi: 10.3389/fnagi.2015.00098] [Medline: 26082715]

120. Alberdi A, Weakley A, Schmitter-Edgecombe M, et al. Smart home-based prediction of multidomain symptoms related to Alzheimer’s disease. IEEE J Biomed Health Inform. Nov 2018;22(6):1720-1731. [doi: 10.1109/JBHI.2018.2798062] [Medline: 29994359]

121. Zolfaghari S, Khodabandehloo E, Riboni D. TraMiner: vision-based analysis of locomotion traces for cognitive assessment in smart-homes. Cognit Comput. 2022;14(5):1549-1570. [doi: 10.1007/s12559-020-09816-3] [Medline: 33552305]

122. Dawadi PN, Cook DJ, Schmitter-Edgecombe M. Automated cognitive health assessment from smart home-based behavior data. IEEE J Biomed Health Inform. Jul 2016;20(4):1188-1194. [doi: 10.1109/JBHI.2015.2445754] [Medline: 26292348]

123. Alsubai S, Alqahtani A, Sha M, Abbas S, Gregus M, Furda R. Automated cognitive health assessment based on daily life functional activities. Comput Intell Neurosci. 2023;2023:5684914. [doi: 10.1155/2023/5684914] [Medline: 37455767]

124. Méndez JI, Meza-Sánchez AV, Ponce P, et al. Smart homes as enablers for depression pre-diagnosis using PHQ-9 on HMI through fuzzy logic decision system. Sensors (Basel). Nov 26, 2021;21(23):7864. [doi: 10.3390/s21237864] [Medline: 34883868]

125. Faruk T, Shum LC, Iaboni A, Khan SS. Walking path images from real-time location data predict degree of cognitive impairment. Artif Intell Med. Oct 2023;144:102657. [doi: 10.1016/j.artmed.2023.102657] [Medline: 37783548]

126. Khosroazad S, Abedi A, Hayes MJ. Sleep signal analysis for early detection of Alzheimer’s disease and related dementia (ADRD). IEEE J Biomed Health Inform. May 2023;27(5):2264-2275. [doi: 10.1109/JBHI.2023.3235391] [Medline: 37018587]

127. Toth L, Hoffmann I, Gosztolya G, et al. A speech recognition-based solution for the automatic detection of mild cognitive impairment from spontaneous speech. Curr Alzheimer Res. 2018;15(2):130-138. [doi: 10.2174/1567205014666171121114930] [Medline: 29165085]

128. Thomas JA, Burkhardt HA, Chaudhry S, et al. Assessing the utility of language and voice biomarkers to predict cognitive impairment in the Framingham heart study cognitive aging cohort data. J Alzheimers Dis. 2020;76(3):905-922. [doi: 10.3233/JAD-190783] [Medline: 32568190]

129. Badal VD, Nebeker C, Shinkawa K, et al. Do words matter? Detecting social isolation and loneliness in older adults using natural language processing. Front Psychiatry. 2021;12:728732. [doi: 10.3389/fpsyt.2021.728732] [Medline: 34867518]

130. Diaz-Asper C, Chandler C, Turner RS, Reynolds B, Elvevåg B. Increasing access to cognitive screening in the elderly: applying natural language processing methods to speech collected over the telephone. Cortex. Nov 2022;156:26-38. [doi: 10.1016/j.cortex.2022.08.005] [Medline: 36179481]

131. Amini S, Hao B, Zhang L, et al. Automated detection of mild cognitive impairment and dementia from voice recordings: a natural language processing approach. Alzheimers Dement. Mar 2023;19(3):946-955. URL: <https://alz-journals.onlinelibrary.wiley.com/toc/15525279/19/3> [Accessed 2026-08-21] [doi: 10.1002/alz.12721]

132. Pourramezan Fard A, Mahoor MH, Alsuhaibani M, Dodge HH. Linguistic-based mild cognitive impairment detection using informative loss. Comput Biol Med. Jun 2024;176:108606. [doi: 10.1016/j.compbiomed.2024.108606] [Medline: 38763068]

133. Zhang Z, Wang T, Hu Z, Yang LZ, Li H. DEMENTIA: a hybrid attention-based multimodal and multi-task learning framework with expert knowledge for Alzheimer’s disease assessment from speech. IEEE J Biomed Health Inform. ;29(4):2957-2968. [doi: 10.1109/JBHI.2024.3509620]

134. Peng M, Zhang Y, Fan Y, Zhang M, Ishimaru M, Li C, et al. Rapid detection of mild cognitive impairment using natural language processing. Chin J Phys Med Rehabil. 2023;45(7):592-597. [doi: 10.3760/cma.j.issn.0254-1424.2023.07.003]

135. Ambrosini E, Giangregorio C, Lomurno E, et al. Automatic spontaneous speech analysis for the detection of cognitive functional decline in older adults: multilanguage cross-sectional study. JMIR Aging. Apr 29, 2024;7:e50537. [doi: 10.2196/50537] [Medline: 38386279]

136. Smith M, Dietrich BJ, Bai EW, Bockholt HJ. Vocal pattern detection of depression among older adults. Int J Ment Health Nurs. Jun 2020;29(3):440-449. [doi: 10.1111/inm.12678] [Medline: 31811697]

137. Mazur A, Costantino H, Tom P, Wilson MP, Thompson RG. Evaluation of an AI-based voice biomarker tool to detect signals consistent with moderate to severe depression. Ann Fam Med. Jan 27, 2025;23(1):60-65. [doi: 10.1370/afm.240091] [Medline: 39805690]

138. Song XD, Wallace BM, Gardner JR, Ledbetter NM, Weinberger KQ, Barbour DL. Fast, continuous audiogram estimation using machine learning. Ear Hear. 2015;36(6):e326-35. [doi: 10.1097/AUD.0000000000000186] [Medline: 26258575]

139. Heisey KL, Walker AM, Xie K, Abrams JM, Barbour DL. Dynamically masked audiograms with machine learning audiometry. Ear Hear. 2020;41(6):1692-1702. [doi: 10.1097/AUD.0000000000000891] [Medline: 33136643]

140. Zhang M, Bi Z, Fu X, et al. A parsimonious approach for screening moderate-to-profound hearing loss in a community-dwelling geriatric population based on a decision tree analysis. BMC Geriatr. Aug 7, 2019;19(1):214. [doi: 10.1186/s12877-019-1232-x] [Medline: 31390985]

141. Charih F, Bromwich M, Mark AE, Lefrançois R, Green JR. Data-driven audiogram classification for mobile audiometry. Sci Rep. Mar 3, 2020;10(1):3962. [doi: 10.1038/s41598-020-60898-3] [Medline: 32127604]

142. Kassjański M, Kulawiak M, Przewoźny T, et al. Automated hearing loss type classification based on pure tone audiometry data. Sci Rep. Jun 20, 2024;14(1):14203. [doi: 10.1038/s41598-024-64310-2] [Medline: 38902305]

143. Wallaert N, Perry A, Jean H, Creff G, Godey B, Paraouty N. Performance and reliability evaluation of an automated bone-conduction audiometry using machine learning. Trends Hear. 2024;28:23312165241286456. [doi: 10.1177/23312165241286456] [Medline: 39489159]

144. Souillard-Mandar W, Penney D, Schaible B, Pascual-Leone A, Au R, Davis R. DCTclock: clinically-interpretable and automated artificial intelligence analysis of drawing behavior for capturing cognition. Front Digit Health. 2021;3:750661. [doi: 10.3389/fdgth.2021.750661] [Medline: 34723243]

145. Kobayashi M, Yamada Y, Shinkawa K, Nemoto M, Nemoto K, Arai T. Automated early detection of Alzheimer’s disease by capturing impairments in multiple cognitive domains with multiple drawing tasks. J Alzheimers Dis. 2022;88(3):1075-1089. [doi: 10.3233/JAD-215714] [Medline: 35723100]

146. Ruengchaijatuporn N, Chatnuntawech I, Teerapittayanon S, et al. An explainable self-attention deep neural network for detecting mild cognitive impairment using multi-input digital drawing tasks. Alz Res Therapy. Aug 9, 2022;14(1):35945568. [doi: 10.1186/s13195-022-01043-2]

147. Kawa J, Bednorz A, Stępień P, Derejczyk J, Bugdol M. Spatial and dynamical handwriting analysis in mild cognitive impairment. Comput Biol Med. Mar 1, 2017;82(21-8):21-28. [doi: 10.1016/j.compbiomed.2017.01.004] [Medline: 28126631]

148. Kang L, Zhang X, Guan J, Huang K, Wu R. Early Alzheimer’s disease diagnosis via handwriting with self-attention mechanisms. J Alzheimers Dis. Nov 2024;102(1):173-180. [doi: 10.1177/13872877241283920] [Medline: 39497308]

149. Yoshii K, Nishimura M, Kimura D, et al. A study for detecting mild cognitive impairment by analyzing conversations with humanoid robots. Presented at: 2021 IEEE 3rd Global Conference on Life Sciences and Technologies (LIFETECH); 347-350; Nara, Japan. March 9-11, 2021.URL: <https://ieeexplore.ieee.org/xpl/mostRecentIssue.jsp?punumber=9391771> [Accessed 2026-08-21] [doi: 10.1109/LifeTech52111.2021.9391934]

150. Chang YL, Luo DH, Huang TR, Goh JOS, Yeh SL, Fu LC. Identifying mild cognitive impairment by using human-robot interactions. J Alzheimers Dis. 2022;85(3):1129-1142. [doi: 10.3233/JAD-215015] [Medline: 34897086]

151. Nishiura Y, Inoue T, Takaeda K, Kamimura T. Inter- and intra-rater reliability of cognitive assessment conducted by assistive robot for older adults living in the community: a preliminary study. Psychogeriatrics. Jan 2025;25(1):e13203. [doi: 10.1111/psyg.13203] [Medline: 39444248]

152. Tsai CF, Chen CC, Wu EHK, et al. A machine-learning-based assessment method for early-stage neurocognitive impairment by an immersive virtual supermarket. IEEE Trans Neural Syst Rehabil Eng. 2021;29:2124-2132. [doi: 10.1109/TNSRE.2021.3118918] [Medline: 34623270]

153. Kim SY, Park J, Choi H, Loeser M, Ryu H, Seo K. Digital marker for early screening of mild cognitive impairment through hand and eye movement analysis in virtual reality using machine learning: first validation study. J Med Internet Res. Oct 20, 2023;25:e48093. [doi: 10.2196/48093] [Medline: 37862101]

154. Wang Y, Yang P, Yu J, et al. An ensemble learning algorithm for cognitive evaluation by an immersive virtual reality supermarket. IEEE Trans Neural Syst Rehabil Eng. 2024;32:3761-3772. [doi: 10.1109/TNSRE.2024.3470802]

155. Fang L, Fan X, Li A, et al. Detecting Alzheimer’s disease using digital virtual reality cognitive tests. Alzheimers Dement. Dec 2025;21(12):41451798. URL: <https://alz-journals.onlinelibrary.wiley.com/toc/15525279/21/12> [Accessed 2026-08-21] [doi: 10.1002/alz.71039]

156. Fraser KC, Lundholm Fors K, Eckerström M, Öhman F, Kokkinakis D. Predicting MCI status from multimodal language data using cascaded classifiers. Front Aging Neurosci. 2019;11(205):205. [doi: 10.3389/fnagi.2019.00205] [Medline: 31427959]

157. Shah HA, Andberg S, Koivisto AM, Bednarik R. A multimodal approach for early identification of mild cognitive impairment and Alzheimer’s disease with fusion network using eye movements and speech. IEEE Trans Neural Syst Rehabil Eng. 2025;33:1449-1459. [doi: 10.1109/TNSRE.2025.3561043] [Medline: 40232896]

158. Poor FF, Dodge HH, Mahoor MH. A multimodal cross-transformer-based model to predict mild cognitive impairment using speech, language and vision. Comput Biol Med. Nov 2024;182:109199. [doi: 10.1016/j.compbiomed.2024.109199] [Medline: 39332117]

159. Zhang X, Zhang F, Hou S, et al. Effectiveness of digital screening tools in detecting cognitive impairment among community-dwelling elderly in Northern China: a large cohort study. J Prev Alzheimers Dis. Mar 2025;12(3):100080. [doi: 10.1016/j.tjpad.2025.100080] [Medline: 39922758]

160. Lin J, Xu T, Yang X, et al. A detection model of cognitive impairment via the integrated gait and eye movement analysis from a large Chinese community cohort. Alzheimer’s & Dementia. Feb 2024;20(2):1089-1101. [doi: 10.1002/alz.13517]

161. Guimarães V, Sousa I, Cunha R, et al. One-class classification with confound control for cognitive screening in older adults using gait, fingertapping, cognitive, and dual tasks. Comput Methods Programs Biomed. Feb 2025;259:108508. [doi: 10.1016/j.cmpb.2024.108508]

162. Lentzen M, Vairavan S, Muurling M, et al. RADAR-AD: assessment of multiple remote monitoring technologies for early detection of Alzheimer’s disease. Alz Res Therapy. Jan 27, 2025;17(1):39865315. [doi: 10.1186/s13195-025-01675-0]

163. Khosroazad S, Gilbert CF, Aronis JB, et al. Sleep movements and respiratory coupling as a biobehavioral metric for early Alzheimer’s disease in independently dwelling adults. BMC Geriatr. Apr 27, 2023;23(1):252. [doi: 10.1186/s12877-023-03983-2] [Medline: 37106470]

164. Butler PM, Yang J, Brown R, et al. Smartwatch- and smartphone-based remote assessment of brain health and detection of mild cognitive impairment. Nat Med. Mar 2025;31(3):829-839. [doi: 10.1038/s41591-024-03475-9] [Medline: 40038507]

165. Park I, Lee SK, Choi HC, et al. Machine learning-based estimation of the mild cognitive impairment stage using multimodal physical and behavioral measures. Sci Rep. Oct 9, 2025;15(1):35369. [doi: 10.1038/s41598-025-19364-1] [Medline: 41068341]

166. Ming A, Clemens V, Lorek E, et al. Game-based assessment of peripheral neuropathy combining sensor-equipped insoles, video games, and AI: proof-of-concept study. J Med Internet Res. Oct 1, 2024;26:e52323. [doi: 10.2196/52323] [Medline: 39353184]

167. Ming A, Schubert T, Marr V, Hötzsch J, Stober S, Mertens PR. Video game-based application for fall risk assessment: a proof-of-concept cohort study. EClinicalMedicine. Dec 2024;78:102947. [doi: 10.1016/j.eclinm.2024.102947] [Medline: 39677357]

168. Howcroft J, Kofman J, Lemaire ED. Prospective fall-risk prediction models for older adults based on wearable sensors. IEEE Trans Neural Syst Rehabil Eng. Oct 2017;25(10):1812-1820. [doi: 10.1109/TNSRE.2017.2687100] [Medline: 28358689]

169. Lincke A, Fagerström C, Ekstedt M, Löwe W, Backåberg S. A comparative study of the 2D- and 3D-based skeleton avatar technology for assessing physical activity and functioning among healthy older adults. Health Informatics J. 2023;29(4):14604582231214589. [doi: 10.1177/14604582231214589] [Medline: 37924210]

170. Kong HH, Shin K, Yang DS, Gu HY, Joo HS, Shon HC. Digital assessment of walking ability: Validity and reliability of the automated figure-of-eight walk test in older adults. PLoS One. 2025;20(2):e0316612. [doi: 10.1371/journal.pone.0316612] [Medline: 39928640]

171. Kim S, Kim HS, Yoo JI. Sarcopenia classification model for musculoskeletal patients using smart insole and artificial intelligence gait analysis. J Cachexia Sarcopenia Muscle. Dec 2023;14(6):2793-2803. [doi: 10.1002/jcsm.13356] [Medline: 37884824]

172. Naseem MT, Kim NH, Seo H, et al. Sarcopenia diagnosis using skeleton-based gait sequence and foot-pressure image datasets. Front Public Health. 2024;12(1443188):1443188. [doi: 10.3389/fpubh.2024.1443188] [Medline: 39664552]

173. Wang X, Wang W, Chen M, et al. Using 3D facial information to predict malnutrition and consequent complications. Nutr Clin Pract. Dec 2024;39(6):1354-1363. [doi: 10.1002/ncp.11215] [Medline: 39319394]

174. Di Martino F, Delmastro F, Dolciotti C. Explainable AI for malnutrition risk prediction from m-Health and clinical data. Smart Health. Dec 2023;30:100429. [doi: 10.1016/j.smhl.2023.100429]

175. Armadá-Maresca F, Capote-Díaz M, Cidad-Betegón MDP, et al. Clinical performance of an interactive platform based on artificial intelligence in ophthalmology: experience in a third-level reference center. Front Med (Lausanne). 2025;12:1593556. [doi: 10.3389/fmed.2025.1593556] [Medline: 40837569]

176. Nickels S, Edwards MD, Poole SF, et al. Toward a mobile platform for real-world digital measurement of depression: user-centered design, data quality, and behavioral and clinical modeling. JMIR Ment Health. Aug 10, 2021;8(8):e27589. [doi: 10.2196/27589] [Medline: 34383685]

177. Kim H, Lee S, Lee S, Hong S, Kang H, Kim N. Depression prediction by using ecological momentary assessment, actiwatch data, and machine learning: observational study on older adults living alone. JMIR Mhealth Uhealth. Oct 16, 2019;7(10):e14149. [doi: 10.2196/14149] [Medline: 31621642]

178. Minaeva O, Riese H, Lamers F, Antypa N, Wichers M, Booij SH. Screening for depression in daily life: development and external validation of a prediction model based on actigraphy and experience sampling method. J Med Internet Res. Dec 1, 2020;22(12):e22634. [doi: 10.2196/22634] [Medline: 33258783]

179. Pedrelli P, Fedor S, Ghandeharioun A, et al. Monitoring changes in depression severity using wearable and mobile sensors. Front Psychiatry. 2020;11:584711. [doi: 10.3389/fpsyt.2020.584711] [Medline: 33391050]

180. Opoku Asare K, Moshe I, Terhorst Y, et al. Mood ratings and digital biomarkers from smartphone and wearable data differentiates and predicts depression status: a longitudinal data analysis. Pervasive Mob Comput. Jul 2022;83:101621. [doi: 10.1016/j.pmcj.2022.101621]

181. Song S, Seo Y, Hwang S, Kim HY, Kim J. Digital phenotyping of geriatric depression using a community-based digital mental health monitoring platform for socially vulnerable older adults and their community caregivers: 6-week living lab single-arm pilot study. JMIR Mhealth Uhealth. Jun 17, 2024;12:e55842. [doi: 10.2196/55842] [Medline: 38885033]

182. Zhang Y, Stewart C, Ranjan Y, et al. Large-scale digital phenotyping: Identifying depression and anxiety indicators in a general UK population with over 10,000 participants. J Affect Disord. Apr 2025;375:412-422. [doi: 10.1016/j.jad.2025.01.124]

183. Little B, Alshabrawy O, Stow D, et al. Deep learning-based automated speech detection as a marker of social functioning in late-life depression. Psychol Med. Jul 2021;51(9):1441-1450. [doi: 10.1017/S0033291719003994] [Medline: 31944174]

184. Zhou Y, Han W, Yao X, Xue J, Li Z, Li Y. Developing a machine learning model for detecting depression, anxiety, and apathy in older adults with mild cognitive impairment using speech and facial expressions: a cross-sectional observational study. Int J Nurs Stud. Oct 2023;146:104562. [doi: 10.1016/j.ijnurstu.2023.104562] [Medline: 37531702]

185. Liu Y, Li X, Wang M, et al. Multimodal depression recognition and analysis: facial expression and body posture changes via emotional stimuli. J Affect Disord. Jul 2025;381(44-54):44-54. [doi: 10.1016/j.jad.2025.03.155]

186. Fan LJ, Wang FY, Zhao JH, et al. From physical activity patterns to cognitive status: development and validation of novel digital biomarkers for cognitive assessment in older adults. Int J Behav Nutr Phys Act. Jan 20, 2025;22(1):11. [doi: 10.1186/s12966-025-01706-x] [Medline: 39833903]
