# Supplementary material for: AI-Based Measurement Tools for Intrinsic Capacity in Older Adults: Scoping Review
Source: J Med Internet Res. 2026 Oct 5;28:e90411. doi: 10.2196/90411 (PMC13639135; doi:10.2196/90411)
Supplement: Supplementary file 2 [file jmir-v28-e90411-s004.docx]

Multimedia Appendix 4

Characteristics of AI-based IC measurement tools (expanded version).

Contents

[1.	Type of the AI-enabled digital devices and systems: Computer vision system (30)	1](#_Toc233381092)

[2.	Type of the AI-enabled digital devices and systems: AI-driven health platform (28)	4](#_Toc233381093)

[3.	Type of the AI-enabled digital devices and systems: Wearable device (26)	7](#_Toc233381094)

[4.	Type of the AI-enabled digital devices and systems: Fixed sensor device (3)	10](#_Toc233381095)

[5.	Type of the AI-enabled digital devices and systems: Smart home (11)	11](#_Toc233381096)

[6.	Type of the AI-enabled digital devices and systems: Speech analysis system (11)	13](#_Toc233381097)

[7.	Type of the AI-enabled digital devices and systems: AI audiometer (6)	15](#_Toc233381098)

[8.	Type of the AI-enabled digital devices and systems: Digital pen (5)	17](#_Toc233381099)

[9.	Type of AI-enabled digital devices and systems: Robot (3)	18](#_Toc233381100)

[10.	Type of the AI-enabled digital devices and systems: Virtual reality system (4)	19](#_Toc233381101)

[11.	Type of the AI-enabled digital devices and systems: Multimodal data acquisition devices (34)	20](#_Toc233381102)

**Description of the file:**

Multimedia Appendix 4 presents a table titled “Characteristics of AI-based IC measurement tools (expanded version)”. The 161 included studies are listed according to the type of AI-enabled digital devices and systems in the following order: computer vision systems, AI-driven health platforms, wearable devices, fixed sensor devices, smart homes, speech analysis systems, AI audiometers, digital pens, robots, virtual reality systems, and multimodal data acquisition devices. For each study, the table reports the following information: author, year, IC domain, details of the device, type of digital biomarkers, measurement methods of digital biomarkers, and AI technology and best score.

Table 3 Characteristics of AI-based IC measurement tools (expanded version).

| 1. Type of the AI-enabled digital devices and systems: **Computer vision system (30)** | | | | | |
| --- | --- | --- | --- | --- | --- |
| **Author, Year** | **Domain of**  **IC** | **Details of the device** | **Type of digital**  **biomarkers** | **Measurement methods of digital biomarkers** | **AI technology**  **Best score** |
| Abràmoff M. D. et al., 2010 [36] | Vision | Digital non‑mydriatic fundus  camera | Ocular structural  features | Fundus images (DR) | ML, CV;  AUC- 0.86 |
| Fleming A. D. et al., 2010 [37] | Vision | Digital non‑mydriatic fundus  camera | Ocular structural  features | Fundus images (DR) | ML, CV;  NR |
| Ting D. S. W. et al., 2017 [38] | Vision | Digital non‑mydriatic fundus  camera | Ocular structural  features | Fundus images (DR) | DL, CV;  AUC-0.958 |
| Tufail A. et al., 2017 [39] | Vision | Digital fundus camera | Ocular structural  features | Fundus images (DR) | ML, CV;  NR |
| Abràmoff M. D. et al., 2018 [40] | Vision | Digital non‑mydriatic fundus  camera, IDx‑DR | Ocular structural  features | Fundus images (DR) | ML, DL, CV;  NR |
| Kanagasin-  gam Y. et al., 2018 [41] | Vision | Digital non‑mydriatic fundus  camera | Ocular structural  features | Fundus images (DR) | ML, DL, CV;  NR |
| Keel S. et al., 2018 [42] | Vision | Digital Retinography System,  EyeGrader™ | Ocular structural  features | Fundus images (DR) | DL, CV;  NR |
| van der Heijden A. et al., 2018 [43] | Vision | Digital non‑mydriatic fundus  Camera, IDx-DR 2.0 | Ocular structural  features | Fundus images (DR) | ML, CV;  AUC-0.94 |
| Verbraak F. D. et al., 2019 [44] | Vision | Digital non‑mydriatic fundus  camera, IDx-DR-EU-2.1 | Ocular structural  features | Fundus images (DR) | DL, CV;  NR |
| Du. Z. et al., 2021 [45] | Vision | Digital non‑mydriatic fundus  camera | Ocular structural  features | Fundus images (DR) | DL, CV;  AUC-0.941 |
| Zhang R. et al., 2024 [46] | Vision | Digital non‑mydriatic fundus  camera | Ocular structural  features | Fundus images (retinal and  optic nerve diseases) | DL, CV;  Acc-1.0 |
| Zhu A. et al., 2024 [47] | Vision | Digital non‑mydriatic fundus  camera | Ocular structural  features | Fundus images  (AMD, DR, Glaucoma) | DL, CV;  NR |
| Matsuura T. et al.,  2019 [53] | Cognition | Automatic dual-task system (Kinect) | Gait parameters | Gait in dual-task test | ML, CV;  Acc-0.754 |
| Seifallahi M. et al., 2022 [48] | Cognition | Kinect V.2 camera | Gait parameters | TUG test | DL, CV; AUC-0.972/  Acc-0.95/F1 -0.975 |
| Farrell B. et al., 2024 [52] | Cognition | Regular RGB camera | Gait parameters | Gait by 3 laps on oval path | ML, CV;  Acc-0.868/ F1 -0.814 |
| Satake Y. et al., 2025 [54] | Cognition | Automatic dual-task system  (Microsoft Kinect V2) | Gait parameters | Gait in dual-task test | ML, DL, CV;  AUC-0.981 |
| Kargar A. H. et al., 2014 [51] | Locomotor  capacity | Microsoft Kinect Sensor | Gait parameters | Get-Up-and-Go Test | ML, CV;  Acc-0.674 |
| Altilio R. et al., 2017 [55] | Locomotor  capacity | 8 camera stereophotogrammetric system (ELITE, BTS, Milan, Italy) | Gait parameters | 6 gait cycles on 11m walkway | ML;  Acc-0.968 |
| Dubois A. et al., 2017 [49] | Locomotor  capacity | Microsoft Kinect v2 | Gait parameters | TUG test | ML, CV;  Acc-1.0 |
| Kim J. H. et al., 2024 [56] | Locomotor  capacity | webcams and action cameras | Gait Parameters | walked 5 steps | DL, CV; AUC-0.99/  Acc-0.778/ F1-0.778 |
| Lin S. et al., 2025 [50] | Psychological  capacity | Microsoft Kinect V2 | Gait parameters | Gait on 6m pathway for 2 min | ML, CV;  AUC-0.911 |
| Mengoudi K. et al., 2020 [57] | Cognition | Eye-tracking system (EyeLink 1000 Plus eye tracker + System Setup) | Eye movement features | Eye tracking test | DL;  F1 -0.74 |
| Sun J. et al., 2022 [58] | Cognition | 3D eye-tracking system | Eye movement features | 3D visual paired comparisons task | ML, DL, CV; AUC-0.97/  Acc-0.95/ F1 -0.95 |
| Zuo F. et al., 2024 [59] | Cognition | 3D eye-tracking system | Eye movement features | 3D comprehensive visual task | DL, CV; AUC-0.90/  Acc-0.84/ F1 -0.83 |
| Umeda- Kameyama Y. et al.,2021 [60] | Cognition | NR | Facial features | Photos of neutral expression | DL, CV;  AUC-0.923/Acc-0.926 |
| Wang X, Liu Y. et al., 2024 [61] | Vitality | 3D Camera (VECTRA H1) | Facial features | 3D facial geometry | ML, DL, CV; |
| Ye J. et al., 2023 [62] | Psychological  capacity | Canon camera | Facial features | imitated expressions on screen | ML, DL, CV;  Acc-0.895/ F1-0.895 |
| Amini S. et al., 2021 [63] | Cognition | NR  (Image of CDT from FHS Cohort) | Digital task performance | Images of CDT | DL, CV; AUC-0.923/  Acc-0.919/ F1 - 0.909 |
| Najafi B. et al., 2025 [64] | Cognition | Standard webcam | Body posture features | Elbow flexion-extension test | ML, CV;  AUC- 0.91 |
| Dubois A. et al., 2019 [65] | Locomotor  capacity | Microsoft Kinect v2 | Balance parameters | 8 balance tasks | ML, CV;  NR |

Table 3 (Continued).

| 2. Type of the AI-enabled digital devices and systems: **AI-driven health platform (28)** | | | | | |
| --- | --- | --- | --- | --- | --- |
| **Author, Year** | **Domain of**  **IC** | **Details of the device** | **Type of digital**  **biomarkers** | **Measurement methods of digital biomarkers** | **AI technology**  **Best score** |
| Dahmen J. et al., 2017 [66] | Cognition | dTMT  (Tablet, Samsung Galaxy Tab 2) | Digital task performance | TMT | ML;  R²-0.42 |
| Bergeron M. F. et al.,2019 [67] | Cognition | Online M-CRT test  (HAPPY neuron platform) | Digital task performance | M-CRT test | ML;  AUC-0.77 |
| Jia Z. et al., 2019 [68] | Cognition | Computerized cognitive  assessment system  (Memory Clinic Guard v1.01.01) | Digital task performance | MoCA | ML;  AUC-0.941/ Acc-0.881 |
| Rutkowski T.M. et al., 2020 [69] | Cognition | Emotional arousal and valence  evaluation task system  (Tablet, Touchscreen) | Digital task performance | Emotional arousal and valence evaluation task | ML, DL;  Acc-0.943/ F1-0.944 |
| Zhang Y. et al., 2021 [70] | Cognition | Custom-developed drag & drop software (Laptop with mouse) | Digital task performance | "Drag-and-drop" hand movement task under dual-task paradigm | ML;  Acc-0.895/ F1-0.800 |
| Modarres M. et al., 2023 [71] | Cognition | CognICA app (iPad) | Digital task performance | ICA (integrated cognitive assessment) | ML;  Acc-0.873 |
| Leng F. et al., 2023 [72] | Cognition | The AI Mini-Cog assessment app | Digital task performance | Mini-Cog | ML;  AUC-0.87 |
| Segev O. et al., 2024 [73] | Cognition | Digital DSST APP  (iPad Pro + Apple Pencil 2nd Pen) | Digital task performance | DSST | ML;  AUC-0.891 |
| Huang G. et al.,  2025 [74] | Cognition | TAS Test system | Digital task performance | TAS Test | ML, DL, CV, NLP;  NR |
| Liew T. M. et al.,  2025 [75] | Cognition | PENSIEVE-AI (12.4-inch touch-  screen tablet and a stylus) | Digital task performance | CDT; Cube Copying Test | ML, DL, CV;  AUC-0.932 |
| Lusi A. et al., 2024 [76] | Locomotor  capacity | Movement assessment app  (Smartphone camera,  Vicon Vero 2.2) | Digital task performance | Squat task | ML, CV;  r-0.89 |
| Chung M.K. et al., 2025 [77] | Psychological  capacity | Mobile Study app,  with support from the collaboration  with Samsung Health) | Digital task performance | Reported stressors via open-ended text messages in app | ML, NLP;  F1-0.92 |
| Tang F. et al., 2020 [78] | Cognition | AI Dialogue Agent(chatbot) | Speech and language  features | Via simulated dialogue with AI | Chatbot (RL, DL, NLP);  AUC-0.859/ F1-0.808 |
| Fristed E. et al.,  2022 [79] | Cognition | Novoic App  (Automatic Story Recall Task) | Speech and language  features | Story recall tasks | ML, NLP;  AUC-0.85 |
| Lin D. et al., 2022 [80] | Psychological  capacity | Ellipsis Health App | Speech and language  features | Open-ended speech (talking about concerns, living situation) | ML, DL, NLP;  AUC-0.84 |
| Kaywan P. et al., 2023 [81] | Psychological  capacity | DEPR-A chatbot | Speech and language  features | Interaction with a chatbot | chatbot (ML, NLP);  NR |
| Kim D. et al. ,2026 [82] | Psychological  capacity | Naver Clova AI CareCall  (telephone-based conversational AI call system) | Speech and language  features | Weekly AI voice calls asked about health, sleep, diet, exercise, and outdoor activity; | chatbot (ML, NLP);  Acc-0.852 |
| Haque R. U. et al., 2021 [83] | Cognition | Mobile eye-tracking system  (VisMET app; Tablet, iPad Air 9.7) | Eye movement features | Visuospatial memory eye-tracking task | ML, DL, CV;  AUC-0.76 |
| Li Q. et al., 2024 [84] | Cognition | Mobile eye-tracking system  (Xiaomi Mi 5 Android tablet+  AI-based eye-tracking software) | Eye movement features | Eye tracking test | ML, DL, CV;  AUC-0.91/ Acc-0.88 |
| Vasan C. S. et al., 2023 [85] | Vision | e-Paarvai (Android smartphones  (48MP & 32MP cameras)) | Ocular structural  features | Photos of Cataract-related features via smartphones | DL, CV;  AUC-0.726/ Acc-0.800 |
| Senthil S. et al., 2025 [86] | Vision | Smartphone-based non-mydriatic  fundus camera (Remidio Fundus on  phone, FOP NM 10) | Ocular structural  features | Retinal fundus image by Smartphone | DL, CV;  Acc-0.920 |
| Jaworski D. et al., 2024 [87] | Vision | AI Pupillometer | Ocular functional  features | smartphone-based measurement of PLR | DL, CV;  NR |
| Barbour D. L. et al., 2019 [88] | Hearing | Online ML audiometer (Bonura hearing measurement Website) | Hearing threshold level | Machine learning audiogram curve | ML;  NR |
| Vercammen C. et al., 2025 [89] | Hearing | Self-administered online hearing test platform | Hearing threshold level | Via self-administered test  (questionnaire);  Gaining estimating Hearing report: predicted hearing threshold, audiogram | ML;  AUC-0.963/ Acc-0.910 |
| Alves S.A. et al., 2024 [90] | Locomotor  capacity | The LINDERA Mobility Analysis  mHealth app (10.0.6-10.17.0) | Gait Parameters | Smartphone-based video and questionnaire | ML, DL, CV;  NR |
| Opoku Asare K. et al., 2021 [91] | Psychological  capacity | The Carat app | Passive mobile sensing  data | e.g., screen status, app usage, internet connectivity | ML;  AUC-0.929/ F1-0.898 |
| Ferreira T. J. et al., 2025 [92] | Vitality | Shaped AI 2-D-photo app  (iPhone 11) | Anthropometric  parameters | (BFP, SFT, Height, Weight, BMI) via Frontal and right-side photos to estimate | ML, DL, CV；  NR |
| Lin N. et al.,  2024 [160] | Vitality | Clinical nutrition assessment  software platform (R+ Dietitia),  (Personal Smartphone camera) | Facial features,  Anthropometric  parameters | Photos of participants' faces; combined with clinical input  (Gender, height, weight) | ML, DL, CV;  AUC-0.933/ Acc> 0.9 |

Table 3 (Continued).

| 3. Type of the AI-enabled digital devices and systems: **Wearable device (26)** | | | | | |
| --- | --- | --- | --- | --- | --- |
| **Author, Year** | **Domain of**  **IC** | **Details of the device** | **Type of digital biomarkers** | **Measurement methods of digital biomarkers** | **AI technology**  **Best score** |
| Obuchi S. P. et al., 2024 [94] | Cognition | Multi-site sensors: Wearable sensors for hip and ankle | Gait parameters | Walking trials with sensors | ML, DL; AUC-0.833/  Acc-0.913/ F1-0.880 |
| Moon G. et al. ,2025 [96] | Cognition | Multi-site sensors: Wearable sensors for Both lower limbs and the waist | Gait parameters | Participants walked eight trials along an 8 m walkway at a comfortable pace | ML;  AUC-0.924 |
| Mannini A. et al., 2016 [95] | Locomotor  capacity | Multi-site sensors:  Wearable sensors for feet, shins,  and lumbar spine (IMUs) | Gait parameters | 10 m walks | ML;  Acc-0.952 |
| Nguyen T. O. et al., 2019 [97] | Locomotor  capacity | Trunk sensors:  Wearable sensors for lower back,  Wireless Gait Analysis Sensor | Gait parameters | 6 gait tasks (DGI tests) | ML; AUC-0.995/  Acc-0.967/ F1-0.965 |
| Buissière F. et al. ,2020 [98] | Locomotor  capacity | Trunk sensors:  Wearable sensors for lower back | Gait parameters | TUG and 6-minute walk test | DL;  AUC-0.821/ Acc-0.830 |
| Seifer A. K. et al., 2024 [100] | Locomotor  capacity | Head sensors:  Hearing aid with IMU | Gait parameters | via 8m walking paths at various speeds | ML, DL;  R²-0.84 |
| Wipperman M. T. et al., 2024 [101] | Locomotor  capacity | Limb sensors:  Digital wearable insole (Rego, AD, Munich, Germany) | Gait parameters | walked on hallway | ML, DL;  AUC-0.99 |
| Yan F. et al., 2024 [102] | Locomotor  capacity | Joints sensors:  Wearable sensors for major motion joints | Gait parameters  (motion primitives) | Gait tasks, Sit-to-Stand, Upper-limb tasks | ML;  AUC-0.95/ Acc-0.83 |
| Cai J. et al., 2025 [103] | Locomotor  capacity | Limb sensors:  Wearable sensors for foot | Gait parameters | walk; stand still 30 s, walk at natural pace | ML, DL;  Acc-0.96 |
| Jung D. et al., 2022 [99] | Psychological  capacity | Trunk sensors:  Wearable sensors for lower back:  Trigno Avanti Sensor | Gait parameters | walked on a 7-meter path | ML, DL;  AUC-0.956/ Acc-0.955 |
| Jung D. et al., 2020 [27] | Cognition,  Locomotor Capacity,  Vitality | Multi-site sensors:  Wearable sensors for foot, shank,  thigh, pelvis (IMUs) | Gait parameters | Gait in 20 seconds at different speeds | ML, DL, CV;  Acc-0.915 |
| Arshad M. Z. et al., 2021 [28] | Locomotor  Capacity,  Vitality | Multi-site sensors:  Wearable sensors for foot and  Pelvis (IMUs) | Gait parameters | 20m walk, repeated  6 times | DL;  Acc-0.851/ F1-0.878 |
| Jung D. et al., 2021 [29] | Locomotor  Capacity,  Vitality | Limb sensors:  Wearable sensors for foot  (IMUs) | Gait parameters | 7-meter walk test at self-selected pace | ML, DL;  Acc-0.950/ F1-0.955 |
| Ghosh S. et al., 2018 104 [104] | Cognition | Limb sensors:  Wearable sensors for wrist,  (uSense and Samsung SIII) | Physical activity  features | Continuous data collection in daily life | ML;  AUC-0.91/ Acc-0.99 |
| Fan L. et al., 2025 [186] | Cognition | Limb sensors:  Wearable sensors for wrist  (from NHATS，NHANES database) | Physical activity  features | Continuous data collection in daily life | ML;  AUC-0.901/Acc-0.842 |
| Gramkow M. H. et al., 2025 105 [105] | Cognition | Trunk sensors: Wearable sensors  for sternum and thigh | Physical activity  features | Continuous data collection in daily life | ML;  Acc-0.89 |
| Jacobson N.C. et al., 2019 106 [106] | Psychological  capacity | Limb sensors:  Wearable sensors for Wrist (Actiwatch) | Physical activity  features | Continuous data collection in daily life | ML;  Acc-0.891 |
| Rykov Y. et al.,2021 107 [107] | Psychological  capacity | Limb sensors:  Wearable sensors for Wrist  (Fitbit Charge 2) | Physical activity  features | Continuous data collection in daily life | ML;  NR |
| Yang D. et al., 2021 [108] | Cognition | fNIRS system  (NIRSIT continuous wave system,  OBELAB Inc.) | fNIRS signals | fNIRS signals recorded during resting-state | DL;  Acc-0.933/ F1-0.933 |
| Ho T.K. et al., 2022 [109] | Cognition | fNIRS system (NIRScout) | fNIRS signals | fNIRS signals recorded during cognitive tasks | DL;  Acc-0.952/ F1-0.952 |
| Pu Z. et al.,  2025 [110] | Cognition | fNIRS system  (NIRST, OBELAB, Seoul, South  Korea) | fNIRS signals | fNIRS signals recorded during cognitive tasks | ML;  AUC-0.97 |
| Choi J. et al., 2022 [111] | Psychological  capacity | Limb sensors:  Wearable sensors for Wrist  (Empatica E4 wristband) | Physiological signals  (PPG, EDA, TEMP);  Physical activity features | Continuous data collection in daily life | ML;  Acc-0.800/ F1-0.779 |
| Lee T. R. et al., 2024 [112] | Psychological  capacity | Limb sensors:  Wearable sensors for wrist  (Fitbit Alta HR2) | Physiological signals (HR)  Physical activity features | Continuous data collection in daily life | DL;  Acc-0.951/ F1-0.923 |
| Rykov Y. et al., 2024 [113] | Psychological  capacity | Limb sensors:  Wearable sensors for wrist  (Empatica E4) | Physiological signals  (HRV, EDA, PPG, T);  Physical activity features | Continuous data collection in daily life | ML, DL;  NR |
| Han S. et al., 2024 [114] | Vitality | Flexible-Printed Piezoelectric  Sensor Array (like smart insoles) | Plantar pressure  parameters | walked 6 m at a normal speed | ML;  Acc-0.968/ F1-0.968 |
| Park C. et al., 2021 [30] | Locomotor capacity,  Cognition,  Vitality | Trunk sensors:  Wearable sensors for sternum,  (PAMSys™ pendant sensor) | Gait parameters  Physical activity features | Continuous data collection in daily life | ML;  AUC-0.80/ Acc-0.81 |

Table 3 (Continued).

| 4. Type of the AI-enabled digital devices and systems: **Fixed sensor device (3)** | | | | | |
| --- | --- | --- | --- | --- | --- |
| **Author, Year** | **Domain of**  **IC** | **Details of the device** | **Type of digital**  **biomarkers** | **Measurement methods of digital biomarkers** | **AI technology**  **Best score** |
| Lai D. T. H. et al., 2008 [115] | Locomotor  capacity | 2D-Motion capture apparatus  (treadmill+ 2D-motion analysis system (PEAK MOTUS 2D)) | Gait parameters | Key gait parameters (MTC)  during treadmill walking | ML; AUC-1.0/  Acc-1.0/F1-1.0 |
| Ballesteros J. et al., 2017 [116] | Locomotor  capacity | i-Walker (smart rollator) | Gait parameters | Walking with i-walker | ML;  R²-0.93 |
| Wu L.-W. et al., 2022 [117] | Vitality | non-invasive arterial applanation  tonometry system (Colin VP-1000) | Physiological signals | BPW (blood pressure waveform), HR | ML;  AUC-0.77 |

Table 3 (Continued).

| 5. Type of the AI-enabled digital devices and systems: **Smart home (11)** | | | | | |
| --- | --- | --- | --- | --- | --- |
| **Author, Year** | **Domain of**  **IC** | **Details of the device** | **Type of digital**  **biomarkers** | **Measurement methods of digital biomarkers** | **AI technology**  **Best score** |
| Dawadi P. N. et al., 2013 [118] | Cognition | CASAS Smart Home Test Bed | Digital task performance | Day Out Task (DOT) | ML; AUC-0.940/  Acc-0.821/F1-0.850 |
| König A. et al., 2015 [119] | Cognition | Event Monitoring System (EMS) | Digital task performance | IADL task | ML, CV;  Acc-0.775 |
| Alberdi A. et al., 2018 [120] | Cognition | NR (installed smart home sensor  system) | Digital task performance | ADLs task | ML;  AUC-0.77 |
| Zolfaghari S. et al., 2022 [121] | Cognition | TraMiner system  (Sensor -based platform) | Digital task performance | Day Out Task (DOT) | ML, DL, CV;  F1-0.85 |
| Dawadi P. N. et al., 2016 [122] | Cognition | CASAS Smart Home system | Physical activity features,  Digital task performance | Mobility, Sleep patterns; ADLs task | ML;  AUC-0.830/ Acc-0.820 |
| Alsubai S. et al., 2023 [123] | Cognition | CASAS dataset-based sensor  platform | Physical activity features,  Digital task performance | Mobility, Sleep patterns; ADLs task | ML, DL; AUC-0.970/  Acc-0.940/ F1-0.943 |
| Mendez J. I. et al.,  2021 [124] | Psychological  capacity | NR; general smart appliances and  systems with sensors | Physical activity features,  Digital task performance | Sensor-based tracking of user behaviors, speech, and smart appliance interactions;  PHQ-9 by smart home | KRR;  NR |
| Faruk T. et al., 2023 [125] | Cognition | A Real-Time Location Sensor  System | Gait parameters | walked in a monitored area equipped with RTLS sensors | DL;  Acc-0.978/ F1-0.978 |
| Khosroazad S., Abedi A. et al., 2023 [126] | Cognition | SleepMove™ (Sensor-based smart  mattress system) | Sleep parameters | Sleep signals captured and processed offline | ML;  Acc-0.975 |
| Nejadshamsi S. et al., 2025 [26] | Psychological  capacity | Wi-Fi–based motion sensor system | Physical activity  features | Passive monitoring; Sleep, daily activity levels | ML;  Acc-0.875/F1-0.860 |
| Schütz N. et al., 2022 [31] | Cognition,  Locomotor,  Vitality,  Psychological | DomoCare (environmental sensor  system), EMFIT QS (bed Sensor) | Physical activity Features;  sleep parameters;  physiological signals | Passive monitoring; Walk, sleep, heart rate | ML;  AUC-0.78 |

Table 3 (Continued).

| 6. Type of the AI-enabled digital devices and systems: **Speech** **analysis system (11)** | | | | | |
| --- | --- | --- | --- | --- | --- |
| **Author, Year** | **Domain of**  **IC** | **Details of the device** | **Type of digital**  **biomarkers** | **Measurement methods of digital biomarkers** | **AI technology**  **Best score** |
| Toth L. et al., 2018 [127] | Cognition | Recording system +  PRAAT software, ASR based tool | Speech and language  features | Narrative and recall tasks:  Watch the animation, Retell and describe yesterday's experiences | ML, DL;  AUC-0.676/Acc-0.750/  F1-0.788 |
| Thomas J.A. et al., 2020 [128] | Cognition | Recording system +  Python (NLTK, SpaCy) | Speech and language  features | Voice recordings during NPT | ML, NLP;  AUC-0.88 |
| Diaz-Asper C. et al., 2022 [130] | Cognition | Cisco Jabber (Phone-call system) +  Python (NLTK) | Speech and language  features | Short story recall and semantic fluency tasks: animal naming and free speech | ML, NLP; AUC-0.93/  Acc-0.84/F1-0.83 |
| Amini S. et al., 2023 [131] | Cognition | Recording system (NR, FHS dataset  with recorded NPT sessions) +  Google Speech Transcription,  NLP model(ALBERT、BERT) | Speech and language  features | Voice recordings during NPT | ML, DL, NLP;  AUC-0.89/Acc-0.87/  F1-0.87 |
| Peng M. et al., 2023 [134] | Cognition | Computer with microphone+  Panasonic proprietary software,  A speech analysis program for  Python (version V3.7.9) | Speech and language  features | Standardized pronunciation  task: syllable repetition  (pa/ta/ka), long vowels. | NLP;  Acc-0.837 |
| Ambrosini E. et al., 2024 [135] | Cognition | Computer with microphone+  PRAAT software, R programming  language, Python | Speech and language  features | Spontaneous speech samples via storytelling and reading tasks. | ML, NLP;  AUC-0.91 |
| Pourramezan Fard A. et al., 2024 [132] | Cognition | Recording system (NR, from  I-CONCET datasets) +  NLP model (BERT),  Python (TensorFlow) | Speech and language  features | Semi-structured interviews transcribed and tokenized:  topics such as daily life, health, and social interaction | DL, NLP; AUC-0.923/  Acc-0.843/F1-0.841 |
| Zhang Z. et al.o, 2025 [133] | Cognition | Recording system (NR, from  ADReSS dataset, Pitt dataset) +  DEMENTIA Model | Speech and language  features | Narrative task describing the 'Cookie Theft' picture | ML, DL, NLP,  KRR;  Acc-0.901 |
| Smith M. et al., 2020 [136] | Psychological  capacity | Sony digital recorder+  Audacity (V 2.3.2), openSMILE,  R programming language | Speech and language  features | Semi-structured interview:  read passages and answered questions | ML;  AUC-0.96 |
| Badal V. D. et al., 2021 [129] | Psychological  capacity | Recording system (NR) + Python | Speech and language  features | Semi-structured interviews: topics such social relationships, emotional support and daily communication situations,  Interview audio transcription | ML, NLP;  AUC-0.79 |
| Mazur A. et al., 2025 [137] | Psychological  capacity | Personal computer + Kintsugi Voice AI tool | Speech and language  features | ≥25s of free-form English speech (How was your day?) | ML, NLP;  NR |

Table 3 (Continued).

| 7. Type of the AI-enabled digital devices and systems: **AI audiometer (6)** | | | | | |
| --- | --- | --- | --- | --- | --- |
| Author, Year | Domain of  IC | Details of the device | Type of digital  biomarkers | Measurement methods of digital biomarkers | AI technology  Best score |
| Song X. D. et al., 2015 [138] | Hearing | Automated ML-based audiometer  (Toshiba Portege R700 laptop computer +  running custom MatLab code +  Sennheiser HD280 circumaural headphones.) | Hearing threshold level | ML method can output reliable continuous threshold estimates at all frequencies, only fewer tests are needed | ML;  NR |
| Zhang M. et al., 2019 [140] | Hearing | Portable automated audiometer Automated hearing threshold audiometry system (Interacoustics MA52 audiometer) +supra-aural headphones (TDH-39) +Decision Tree Analysis | Hearing threshold level | Via PTA, ML output is the classification result  (whether there is hearing loss ≥40 dB HL) | ML;  Acc-0.89 |
| Charih F. et al., 2020  [141] | Hearing | Calibrated audiometer  (The NHANES dataset, conventional audiometer+  supra-aural or insert headphones) | Hearing threshold level | Audiogram from NHANES and clinical sources, ML output is  configuration, severity, symmetry | ML;  Acc-0.93/ F1-0.93 |
| Heisey K. L. et al., 2020 [139] | Hearing | ML audiometer  (Dell XPS laptop+ TDH-50P Telephonic supra-aural  headphones +AMALG) | Hearing threshold level | Audiogram, can be directly converted to PTA | ML;  NR |
| Kaszyński M. et al., 2024 [142] | Hearing | Clinical audiometer  Itera II and Midimate 622 clinical audiometers  (Madsen Electronics, Otometrics)  (The tonal audiometry dataset) | Hearing threshold level | Via PTA, Classification (such as sensorineural/  conductive/mixed loss) | DL;  Acc-0.956 |
| Wallaert N. et al., 2024 [143] | Hearing | Automated ML-Based BC  audiometer (computer+ headphones+  Radioear B81 Bone Conduction Transducers+ ML) | Hearing threshold level | Via AC PTA, BC PTA | ML;  NR |

Table 3 (Continued).

| 8. Type of the AI-enabled digital devices and systems: **Digital pen (5)** | | | | | |
| --- | --- | --- | --- | --- | --- |
| **Author, Year** | **Domain of**  **IC** | **Details of the device** | **Type of digital**  **biomarkers** | **Measurement methods of digital biomarkers** | **AI technology**  **Best score** |
| Souillard-Mandar W. et al., 2021 [144] | Cognition | DCT clock platform,  Digital Pen (NR) | Digital task performance | CDT | ML, DL, CV;  AUC-0.89 |
| Kobayashi M. et al., 2022 [145] | Cognition | Wacom Cintiq Pro 16, digital pen | Digital task performance | Sentence copying, polygon copying, CDT, TMT | ML; AUC-0.94/  Acc-0.88/ F1-0.88 |
| Ruengchai-jatuporn N. et al., 2022 [146] | Cognition | Tablet with digital pen | Digital task performance | CDT, Cube Copying Test, TMT | ML, DL, CV; AUC-0.838  Acc- 0.812/ F1- 0.654 |
| Kawa J. et al., 2017 [147] | Cognition | Livescribe Echo Pen | Handwriting features | writing tasks | ML;  Acc-0.70 |
| Kang L. et al., 2024 [148] | Cognition | NR (handwriting data collected from DARWIN dataset) | Handwriting features | 25 handwriting tasks | DL;  Acc-0.931/F1-0.928 |

Table 3 (Continued).

| 9. Type of AI-enabled digital devices and systems: Robot (3) | | | | | |
| --- | --- | --- | --- | --- | --- |
| **Author, Year** | **Domain of**  **IC** | **Details of the device** | **Type of digital**  **biomarkers** | **Measurement methods of digital biomarkers** | **AI technology**  **Best score** |
| Yoshii K. et al., 2021 [149] | Cognition | Softbank Pepper,  lavalier microphone | Speech and language  features | MMSE, daily conversational  speech with robot | NLP, Robotics;  NR |
| Chang Y. L. et al., 2022 [150] | Cognition | RobiRobot® | Digital task performance | PM, RM, CWFT, CVLT-II by robot | KRR, Robotics;  NR |
| Nishiura Y. et al., 2025 [151] | Cognition | PaPeRo | Digital task performance | COGNISTAT by robot | KRR, Robotics;  NR |

Table 3 (Continued).

| 10. Type of the AI-enabled digital devices and systems: **Virtual reality system (4)** | | | | | |
| --- | --- | --- | --- | --- | --- |
| **Author, Year** | **Domain of**  **IC** | **Details of the device** | **Type of digital**  **biomarkers** | **Measurement methods of digital biomarkers** | **AI technology**  **Best score** |
| Tsai C. F. et al., 2021 [152] | Cognition | VR supermarket system | VR task performance | virtual shopping tasks | ML; AUC-1.0/  Acc-1.0/F1-1.0 |
| Kim S.Y. et al., 2023 [153] | Cognition | The virtual kiosk test | VR task performance | Virtual Kiosk Test | ML;  AUC-0.93 |
| Wang Y. et al., 2024 [154] | Cognition | IVRSCAP  (Immersive VR Supermarket  Cognitive Assessment Platform) | VR task performance | virtual shopping tasks | ML;  AUC-0.985/Acc-0.965/  F1-0.965 |
| Fang L. et al. ,2025 [155] | Cognition | AI-VR digital cognitive measurement tool | VR task performance,  Eye movement features | Participants wore the VR device, completed cognitive tasks in VR scenes, and answered by fixating on the correct option; eye trajectories and gaze points were recorded. | ML;  AUC-0.969 |

Table 3 (Continued).

| 11. Type of the AI-enabled digital devices and systems: **Multimodal data acquisition devices (34)** | | | | | |
| --- | --- | --- | --- | --- | --- |
| **Author, Year** | **Domain of**  **IC** | **Details of the device** | **Type of digital**  **biomarkers** | **Measurement methods of digital biomarkers** | **AI technology** |
| Fraser K. C. et al., 2019 [156] | Cognition | - Computer vision system - Eye- tracking system, EyeLink 1000 - Speech analysis system - Voice recording device, H2n Handy Recorder | Eye movement features;  Speech and language  features | Record eye movements while performing the speech (picture description) task. | ML, NLP;  AUC-0.90/  Acc-0.83 |
| Shah H. A. et al., 2025 [157] | Cognition | - Computer vision system - Eye-Tracking device, integrated Tobii TX300 unit - Speech analysis system - recording device | Eye movement features;  Speech and language  Features | Eye-tracking during King-Devick test and reading task, speech analysis via microphone | ML, DL,  NLP, CV;  AUC-0.823/ Acc-0.86/  F1-0.883 |
| Poor F. et al., 2024 [158] | Cognition | Digital Video Recording System  (NR, from I-CONCET datasets)   - Computer vision system - Speech analysis system | Speech and language  features; Facial features;  Body posture features | Dataset collected from pre-recorded interview videos including audio, transcriptions, and facial features | DL, CV,  NLP;  AUC-0.949/  Acc-0.887 |
| Zhang X. et al., 2025 [159] | Cognition | - Digital Pen - Computer vision system - Eye-tracking system (EyeKnow,   tablet computer+ VR headset)   - quantitative motor function evaluation system, ReadyGo | Digital task performance;  Eye movement features;  Gait parameters | CDT;  eye-tracking task;  single and dual task gait tests. | ML, DL, CV;  AUC-0.958/  Acc-0.902 |
| Lin J. et al.,  2024 [160] | Cognition | - Computer vision system - Eye-tracking system (EyeKnow,   tablet computer+ VR headset)   - quantitative motor function assessment system, ReadyGo | Eye movement features;  Gait parameters | Eye movement single-task, eye movement dual-task;  single and dual task gait tests. | ML, DL, CV;  AUC- 0.987/  Acc-0.929 |
| Guimarães V. et al., 2025 [161] | Cognition | - AI-driven health platform - Customized or digital platforms in the laboratory on Smartphone - Wearable device - Wearable sensors for wrist, waist, foot (IMU) | Digital task performance;  Gait parameters | Finger tapping task; dual-task gait | ML;  AUC-0.863/ ACC-0.849 |
| Lentzen M. et al., 2025 [162] | Cognition | - AI-driven health platform - Altoida App, Mezurio App, Banking App (RADAR-AD study platform) - Wearable device - Wearable sensors for wrist, foot (Fitbit Charge 3, Axivity AX3, Physilog sensors) | Digital task performance;  Gait parameters;  Physical activity features;  Speech and language  features | Devices used daily; passive and active tasks; e.g., walking with gait sensor, phone games, verbal recall, banking behavior tracking | ML;  AUC-0.87/ Acc-0.80 |
| Khosroazad S., Gilbert C.  F. et al.,  2023 [163] | Cognition | - Smart Home - Smart Mattress, Move™ - Wearable device - Wearable sensors for wrist, Philips Actiwatch 2 | Sleep parameters;  Physical activity features | Participants monitored for 7 days; underwent 2-night home sleep study. | ML;  AUC-0.88 |
| Butler, P. M. et al., 2025 [164] | Cognition | - Wearable Device - Wearable sensors for Wrist (Apple Watch) - AI-driven health platform - Apple Research app | Physical activity features, Physiological signals;  Digital task performance, Speech and language  Features, Passive mobile  sensing data | Physical activity features, Physiological signals (HR) via Apple Watch; Digital task performance, Speech and language features,  Passive mobile sensing data via app | ML;  AUC-0.85 |
| Park I. et al.,2025 [165] | Cognition | - Wearable Device - Portable polysomnography device - Fixed sensor device - Electronic walkway - DXA scanner | Sleep parameters  Gait parameters;  Anthropometric parameters; | Participants completed gait walking test using GAITRite, DXA body composition assessment, overnight polysomnography, | ML, DL;  AUC-0.94 |
| Ming A.,  Clemens, V.  et al., 2024 [166] | Locomotor capacity | - Wearable device - Sensor-equipped insoles (ActiSense System, IEE S.A.) - AI-driven health platform - Video game-based app | Plantar pressure  Parameters;  Balance parameters. | Wore pressure sensor insoles while playing custom video games; game interaction metrics recorded | ML;  AUC-0.760/  Acc-0.755 |
| Ming A.,  Schubert, T.  et al., 2024 [167] | Locomotor capacity | - Wearable device - smart shoes with pressure sensors, ActiSense System® - AI-driven health platform - Video game-based app | Plantar pressure  parameters;  Balance parameters. | Using smart shoes and tablet-based gaming tasks to assess movement, coordination and gait in seated and standing positions. | ML;  AUC-0.91/  Acc-0.87 |
| Howcroft J.  et al., 2017 [168] | Locomotor capacity | - Wearable device - Pressure-sensing insoles - Wearable sensors for pelvis, head, shanks | Plantar pressure  parameters;  Gait parameters. | ST and DT gait conditions tested | ML;  Acc-0.907/  F1-0.900 |
| Lincke A. et  al., 2023 [169] | Locomotor capacity | - Computer vision system - RGB camera - Kinect v2 sensor - Wearable device - Activity monitoring equipment, ActiPAL | Gait parameters;  Physical activity features | Video of Functional Tests (4SBT, 30sCST, TUG, SCT) put in 2D/3D models analyzed with AI;  the ActivPAL device for 7 consecutive days to record their daily physical activities. | ML, DL, CV;  NR |
| Kong H. H. et al., 2025 [170] | Locomotor capacity | Digital Senior Fitness Test system  (DSFT system)   - Computer vision system - Care-Vision (depth camera) - Fixed sensor device - Care-Pad (force plate) - Care-Grip (electronic hand dynamometer) | Gait parameters,  Balance parameters. | Walking figure-of-eight route(aF8WT); automatic recording via camera and sensors; compared manual vs automated results. | ML, DL, CV;  aF8WT vs  mF8WT, aF8WT test-  retest, ICC-  0.95-0.94. |
| Kim S. et al.,  2023 [171] | Vitality | - Computer vision system - Smartphone, Galaxy A20 - Wearable device - Smart insole system, SALTED | Gait parameters;  Plantar pressure  parameters | Smartphone video for pose; smart insole with IMU and pressure sensors for gait data while walking naturally. | ML, DL, CV;  Acc-0.96/  F1-0.92 |
| Naseem M.  T. et al., 2024 [172] | Vitality | - Computer vision system - The Azure Kinect - Fixed sensor device - Floor pressure plate - digital dynamometer | Gait parameters;  plantar pressure  parameters;  grip strength. | Walked in front of the Azure Kinect on Floor pressure plate;  Handgrip strength via digital dynamometer. | ML, DL, CV;  Acc-0.80 |
| Wang X,  Wang W.  et al., 2024 [173] | Vitality | - Computer vision system - 3D Camera, Vectra H1 - Fixed sensor device - BIA scale (Inbody S10) | Facial features;  Anthropometric  Parameters. | 3D facial structure point cloud via 3D Camera;  (PhA, FFM, ASMI, ECW/ICW, Girth index) via BIA scale. | ML, DL, CV;  AUC-0.791/  Acc-0.742 |
| Di Martino F. et al., 2023 [174] | Vitality | - AI-driven health platform - DIEW app (m-Health platform) - Fixed sensor device - Bluetooth weight scale - BIA scale | Physical activity features;  Anthropometric  Parameters. | Nutrition intake, activity via DIEW app;  Weight, body composition via bluetooth weight scale, BIA scale. | ML;  AUC-0.978/ Acc-0.952/ F1-0.942 |
| Armadá-Maresca F.  et al., 2025 [175] | Vision | - AI-driven health platform - DORIA platform, AI-based interactive platform that integrates data from EyetLib™ - Robot - EyetLib™ Robotized scan | Digital task performance;  Ocular functional  features;  Ocular structural  features; | Autonomous robot performed ocular evaluations; AI-guided questionnaire for symptoms/history; operated by a trained technician; ophthalmologists remotely reviewed data. | ML, DL, CV, NLP;  NR |
| Nickels S. et al., 2021 [176] | Psychological capacity | Custom Verily app   - AI-driven health platform | Passive mobile sensing  data; Physical activity  features; Speech and  language features | Passive sensor collection (accelerometer, GPS, audio, activity recognition) and active tasks (surveys, voice diaries). | ML, NLP;  AUC-0.656 |
| Kim H. et al.,  2019 [177] | Psychological capacity | - Wearable device - Wearable sensors for wrist,   Actiwatch, Philips Respironics   - AI-driven health platform - EMA mobile application | Physical activity features;  EMA features | (Activity level, light exposure, sleep  patterns) via Continuous wrist actigraphy;  self-reported mood and fatigue via EMA app | ML;  AUC-0.95/  Acc-0.92/  F1-0.91 |
| Minaeva O.  et al., 2020 [178] | Psychological capacity | - Wearable device - Wearable sensors for wrist,   Actiwatch   - AI-driven health platform - EMA, ESM APP | Physical activity features;  EMA features. | Activity, sleep via Continuous Actiwatch;  self-reported affect/behaviors via ESM app. | ML;  AUC-0.993 |
| Pedrelli P. et al., 2020 [179] | Psychological capacity | - Wearable device - Wearable sensors for wrist,   Empatica E4   - AI-driven health platform - AWARE App | Physiological signals;  Physical activity features;  Passive mobile sensing  Data. | (EDA, Skin temperature, HRV, HR), (Activity, sleep) via Empatica E4;  (Telephone interaction behavior, Smartphone usage patterns, Location and mobility data (GPS)) via  AWARE App | ML;  R²-0.66 |
| Asare, K. O. et al., 2022 [180] | Psychological capacity | - Wearable device - Oura Ring - AI-driven health platform - AWARE App | Physical activity features;  Passive mobile sensing  data;  EMA features. | wearable sensing (Oura ring for sleep and activity);  passive smartphone sensing (GPS, screen unlocks);  Self-reported daily mood via app | ML;  AUC-0.823/  Acc-0.814 |
| Song S. et al.,  2024 [181] | Psychological capacity | - Wearable device - Wearable sensors for wrist, Galaxy Watch - AI-driven health platform - Custom community mental health platform | Physiological signal;  Physical activity features;  EMA features. | Collected via smartwatch sensors and smartphone app daily; self-reported mood data and automatic physiological metrics (HRV). | KRR;  NR |
| Zhang Y. et  al., 2025 [182] | Psychological capacity | - Wearable device - Wearable sensors for wrist, Fitbit - AI-driven health platform - RADAR-based app | Physical activity features;  Physiological signal;  Digital task performance. | (Sleep, activity), (HR, circadian rhythms) via Fitbit;  PHQ-8, GAD-7 by app. | ML;  R²-0.37 |
| Little B. et al., 2021 [183] | Psychological capacity | Custom wrist-worn acoustic sensor  (With microphone and accelerometer)   - Wearable device - Speech analysis system | Physical activity features  Speech and language  Features. | Wearable device captured continuous speech data and physical activity. | DL;  Acc-0.935 |
| Zhou Y. et al., 2023 [184] | Psychological capacity | - Computer vision system - Video camera (USB webcam) - Speech analysis system - Computer (Microphone, recording software (IFLYTEK S702)) | Facial features;  Speech and language  features. | Speech recorded via microphone during CFAPS task; facial expressions recorded and analyzed using Open Face. | ML, NLP,  CV;  Acc-0.874/  F1-0.966 |
| Liu Y. et al.,  2025 [185] | Psychological capacity | Canon camera (for video recording)   - Computer vision system | Facial features;  Body posture features. | Videos (Facial expressions and body posture features (head, shoulder)) recorded during emotion-stimulus experiment using IAPS images | DL, CV;  Acc-0.904/  F1-0.901 |
| Yan L. et al.,  2023 [32] | Cognition,  Locomotor capacity,  Vitality | Digital rehabilitation evaluation system   - Computer vision system - Wearable device - Fixed sensor device - AI-driven health platform | Digital task performance;  Balance parameters;  Gait parameters;  Grip strength;  Physiological signal;  Anthropometric  Parameters. | Digital task performance (ADL task, MMSE, MoCA, NPI); Stand on the balance board for 30 seconds;  TUG (gait test) in front of 3D camera; Joint range of motion via 3D camera and wearable device; Physiological signal (BP, HR), Anthropometric parameters (BMI, diet, symptoms)  via integrated medical testing equipment | ML, DL, CV,  NLP;  NR |
| Aznar-Grimón R.  et al., 2024 [33] | Cognition,  Vitality | - Wearable device - Wearable sensors for waist, foot   (IMU)   - Fixed sensor device - smartphone (iPhone 15 Pro) - GoPro HERO11 Black | Gait parameters | Walking along an 8-meter corridor with IMU sensors and video recording; turning and walking back | ML, DL, CV;  Acc-0.875/  F1-0.875 |
| Kolak M.  et al., 2025 [34] | Cognition,  Locomotor,  Vitality, Vision, Hearing,  Psychological capacity | - Wearable Device - Withings Scanwatch (Wrist sensors) - AI-driven health platform - CAREUP platforms, Lenovo Tab M10 - Smart Home - iHealth HS2S FIT, GRYP Digital Hand Dynamometer, Withings Sleep Analyzer | Digital task performance;  Grip strength;  Physiological signals;  Physical activity features. | Digital test performance (MoCA, Cognitive game, SPPB, self-report Energy/Fatigue level, self-report Vision and hearing conditions, PHQ-9) via CAREUP platforms;  TUG via Wearable Device;  Physiological signals (BP, SpO₂ , HR, weight), Physical activity features (activity and sleep) via Smart Home. | ML, DL;  R²-0.66 |
| Tavassoli et al. (2022) [35] | Cognition,  Locomotor,  Vitality, Vision, Hearing,  Psychological capacity | - AI-driven health platform - ICOPE MONITOR App - ICOPEBOT Conversational Robot (chatbot) | Digital task performance, Speech and language  features | A series of IC questionnaires via app; chatbot interact with users through conversations and ask them questions related to health | Chatbot (ML, NLP, KRR);  NR |

**References**

**(26 - 186)**

26. Nejadshamsi S, Karami V, Ghourchian N, et al. Development and feasibility study of HOPE model for prediction of depression among older adults using Wi-Fi-based motion sensor data: machine learning study. JMIR Aging. Mar 3, 2025;8:e67715. [doi: 10.2196/67715] [Medline: 40053734]

27. Jung D, Nguyen MD, Park M, et al. Walking-in-place characteristics-based geriatric assessment using deep convolutional neural networks. Annu Int Conf IEEE Eng Med Biol Soc. Jul 2020;2020:3931-3935. [doi: 10.1109/ EMBC44109.2020.9176069] [Medline: 33018860]

28. Arshad MZ, Jung D, Park M, Shin H, Kim J, Mun KR. Gait-based frailty assessment using image representation of IMU signals and deep CNN. Presented at: 2021 43rd Annual International Conference of the IEEE Engineering in Medicine & Biology Society (EMBC); Nov 2021:1874-1879; Mexico. Nov 2021.[doi: 10.1109/EMBC46164.2021.9630976] [Medline: 34891653]

29. Jung D, Kim J, Kim M, Won CW, Mun KR. Frailty assessment using temporal gait characteristics and a long short-term memory network. IEEE J Biomed Health Inform. Sep 2021;25(9):3649-3658. [doi: 10.1109/JBHI.2021.3067931] [Medline: 33755570]

30. Park C, Mishra R, Golledge J, Najafi B. Digital biomarkers of physical frailty and frailty phenotypes using sensor-based physical activity and machine learning. Sensors. Aug 5, 2021;21(16):5289. [doi: 10.3390/s21165289]

31. Schütz N, Knobel SEJ, Botros A, et al. A systems approach towards remote health-monitoring in older adults: introducing a zero-interaction digital exhaust. NPJ Digit Med. Aug 16, 2022;5(1):116. [doi: 10.1038/s41746-022-00657- y] [Medline: 35974156]

32. LiuY, Liu T, Zhao C, Wang X. Research and application of digital rehabilitation evaluation methods for elderly care communities. China Digital Medicine. 2023;18(5):110-116. [doi: 10.3969/j.issn.1673-7571.2023.05.022]

33. Aznar-Gimeno R, Perez-Lasierra JL, Pérez-Lázaro P, et al. Gait-based AI models for detecting sarcopenia and cognitive decline using sensor fusion. Diagnostics (Basel). Dec 22, 2024;14(24):2886. [doi: 10.3390/diagnostics14242886] [Medline: 39767247]

34. Kolakowski M, Lupica A, Ben Bader S, et al. CAREUP: an integrated care platform with intrinsic capacity monitoring and prediction capabilities. Sensors (Basel). Feb 3, 2025;25(3):916. [doi: 10.3390/s25030916] [Medline: 39943555]

35. Tavassoli N, de Souto Barreto P, Berbon C, et al. Implementation of the WHO integrated care for older people (ICOPE) programme in clinical practice: a prospective study. Lancet Healthy Longev. Jun 2022;3(6):e394-e404. [doi: 10.1016/ S2666-7568(22)00097-6] [Medline: 36098317]

36. Abràmoff MD, Reinhardt JM, Russell SR, et al. Automated early detection of diabetic retinopathy. Ophthalmology. Jun 2010;117(6):1147-1154. [doi: 10.1016/j.ophtha.2010.03.046] [Medline: 20399502]

37. Fleming AD, Goatman KA, Philip S, Prescott GJ, Sharp PF, Olson JA. Automated grading for diabetic retinopathy: a large-scale audit using arbitration by clinical experts. Br J Ophthalmol. Dec 2010;94(12):1606-1610. [doi: 10.1136/bjo. 2009.176784] [Medline: 20858722]

38. Ting DSW, Cheung CYL, Lim G, et al. Development and validation of a deep learning system for diabetic retinopathy and related eye diseases using retinal images from multiethnic populations with diabetes. JAMA. Dec 12, 2017;318(22):2211-2223. [doi: 10.1001/jama.2017.18152] [Medline: 29234807]

39. Tufail A, Rudisill C, Egan C, et al. Automated diabetic retinopathy image assessment software: diagnostic accuracy and cost-effectiveness compared with human graders. Ophthalmology. Mar 2017;124(3):343-351. [doi: 10.1016/j.ophtha. 2016.11.014] [Medline: 28024825]

40. Abràmoff MD, Lavin PT, Birch M, Shah N, Folk JC. Pivotal trial of an autonomous AI-based diagnostic system for detection of diabetic retinopathy in primary care offices. NPJ Digit Med. 2018;1(39):39. [doi: 10.1038/s41746-018- 0040-6] [Medline: 31304320]

41. Kanagasingam Y, Xiao D, Vignarajan J, Preetham A, Tay-Kearney ML, Mehrotra A. Evaluation of artificial intelligence-based grading of diabetic retinopathy in primary care. JAMA Netw Open. Sep 7, 2018;1(5):e182665. [doi: 10.1001/jamanetworkopen.2018.2665] [Medline: 30646178]

42. Keel S, Lee PY, Scheetz J, et al. Feasibility and patient acceptability of a novel artificial intelligence-based screening model for diabetic retinopathy at endocrinology outpatient services: a pilot study. Sci Rep. Mar 12, 2018;8(1):4330. [doi: 10.1038/s41598-018-22612-2] [Medline: 29531299]

43. van der Heijden AA, Abramoff MD, Verbraak F, van Hecke MV, Liem A, Nijpels G. Validation of automated screening for referable diabetic retinopathy with the IDx-DR device in the Hoorn Diabetes Care System. Acta Ophthalmol. Feb 2018;96(1):63-68. [doi: 10.1111/aos.13613] [Medline: 29178249]

44. Verbraak FD, Abramoff MD, Bausch GCF, et al. Diagnostic accuracy of a device for the automated detection of diabetic retinopathy in a primary care setting. Diabetes Care. Apr 2019;42(4):651-656. [doi: 10.2337/dc18-0148] [Medline: 30765436]

45. Du Z, Liu J, Hu H, Yang N, Wen L, Wang F, et al. Evaluation of deep learning network model based on attention mechanism in diabetic retinopathy screening in China. Chin J Diabetes Mellitus. 2021;13(12):1148-1154. [doi: 10.3760/ cma.j.cn115791-20210314-00152]

46. Zhang R, Dong L, Fu X, et al. Trends in the prevalence of common retinal and optic nerve diseases in China: an artificial intelligence based national screening. Trans Vis Sci Tech. Apr 2, 2024;13(4):28. [doi: 10.1167/tvst.13.4.28]

47. Zhu A, Tailor P, Verma R, et al. Implementation of deep learning artificial intelligence in vision-threatening disease screenings for an underserved community during COVID-19. J Telemed Telecare. Dec 2024;30(10):1590-1597. [doi: 10.1177/1357633X231158832] [Medline: 36908254]

48. Seifallahi M, Mehraban AH, Galvin JE, Ghoraani B. Alzheimer’s disease detection using comprehensive analysis of timed up and go test via Kinect V.2 Camera and machine learning. IEEE Trans Neural Syst Rehabil Eng. 2022;30(1589-600):1589-1600. [doi: 10.1109/TNSRE.2022.3181252] [Medline: 35675251]

49. Dubois A, Bihl T, Bresciani JP. Automating the timed up and go test using a depth camera. Sensors (Basel). Dec 22, 2017;18(1):14. [doi: 10.3390/s18010014] [Medline: 29271926]

50. Lin S, Li S, Fang Y. Interpretable machine learning for depression recognition with spatiotemporal gait features among older adults: a cross-sectional study in Xiamen, China. BMC Geriatr. Jul 2, 2025;25(1):453. [doi: 10.1186/s12877-025- 06101-6] [Medline: 40604502]

51. Kargar BAH, Mollahosseini A, Struemph T, Pace W, Nielsen RD, Mahoor MH. Automatic measurement of physical mobility in Get-Up-and-Go Test using Kinect sensor. Annu Int Conf IEEE Eng Med Biol Soc. 2014;2014(3492-5):3492-3495. [doi: 10.1109/EMBC.2014.6944375] [Medline: 25570743]

52. Farrell B, Horn J, Seifallahi M, Galvin JE, Ghoraani B. Mild cognitive impairment detection through gait analysis and standard cameras. Annu Int Conf IEEE Eng Med Biol Soc. Jul 2024;2024:1-4. [doi: 10.1109/EMBC53108.2024. 10782745] [Medline: 40039723]

53. Matsuura T, Sakashita K, Grushnikov A, Okura F, Mitsugami I, Yagi Y. Statistical Analysis of Dual-task Gait Characteristics for Cognitive Score Estimation. Sci Rep. Dec 27, 2019;9(1):31882727. [doi: 10.1038/s41598-019-56485- w]

54. Satake Y, Taomoto D, Wu S, et al. The clinical significance of an AI-based assumption model for neurocognitive diseases using a novel dual-task system. Sci Rep. Summer 2025;15(1):40263361. [doi: 10.1038/s41598-025-98319-y]

55. Altilio R, Paoloni M, Panella M. Selection of clinical features for pattern recognition applied to gait analysis. Med Biol Eng Comput. Apr 2017;55(4):685-695. [doi: 10.1007/s11517-016-1546-1] [Medline: 27435068]

56. Kim JH, Hong H, Lee K, et al. AI in evaluating ambulation of stroke patients: severity classification with video and functional ambulation category scale. Top Stroke Rehabil. Jan 2, 2025;32(1):34-42. [doi: 10.1080/10749357.2024. 2359342]

57. Mengoudi K, Ravi D, Yong KXX, et al. Augmenting dementia cognitive assessment with instruction-less eye-tracking tests. IEEE J Biomed Health Inform. Nov 2020;24(11):3066-3075. [doi: 10.1109/JBHI.2020.3004686] [Medline: 32749977]

58. Sun J, Liu Y, Wu H, Jing P, Ji Y. A novel deep learning approach for diagnosing Alzheimer’s disease based on eye- tracking data. Front Hum Neurosci. 2022;16:972773. [doi: 10.3389/fnhum.2022.972773] [Medline: 36158627]

59. Zuo F, Jing P, Sun J, Duan J, Ji Y, Liu Y. Deep learning-based eye-tracking analysis for diagnosis of Alzheimer’s disease using 3D comprehensive visual stimuli. IEEE J Biomed Health Inform. May 2024;28(5):2781-2793. [doi: 10. 1109/JBHI.2024.3365172] [Medline: 38349825]

60. Umeda-Kameyama Y, Kameyama M, Tanaka T, et al. Screening of Alzheimer’s disease by facial complexion using artificial intelligence. Aging (Albany NY). Jan 25, 2021;13(2):1765-1772. [doi: 10.18632/aging.202545] [Medline: 33495415]

61. Wang X, Liu Y, Rong Z, et al. Development and evaluation of a deep learning framework for the diagnosis of malnutrition using a 3D facial points cloud: a cross‐sectional study. J Parenter Enteral Nutr. Jul 2024;48(5):554-561. [doi: 10.1002/jpen.2643]

62. Ye J, Yu Y, Fu G, et al. Analysis and recognition of voluntary facial expression mimicry based on depressed patients. IEEE J Biomed Health Inform. Aug 2023;27(8):3698-3709. [doi: 10.1109/JBHI.2023.3260816] [Medline: 37030686]

63. Amini S, Zhang L, Hao B, et al. An artificial intelligence-assisted method for dementia detection using images from the clock drawing test. J Alzheimers Dis. 2021;83(2):581-589. [doi: 10.3233/JAD-210299] [Medline: 34334396]

64. Najafi B, Lee M, Dehghan Rouzi M, Runyon JR, Sternberg EM, LaFleur BJ. A 20-s video-based assessment of cognitive frailty: results from a cohort study within the precision aging network. Gerontology. 2025;71(7):589-600. [doi: 10.1159/000546227] [Medline: 40359928]

65. Dubois A, Mouthon A, Sivagnanaselvam RS, Bresciani JP. Fast and automatic assessment of fall risk by coupling machine learning algorithms with a depth camera to monitor simple balance tasks. J Neuroeng Rehabil. Jun 11, 2019;16(1):71. [doi: 10.1186/s12984-019-0532-x] [Medline: 31186002]

66. Dahmen J, Cook D, Fellows R, Schmitter-Edgecombe M. An analysis of a digital variant of the Trail Making Test using machine learning techniques. Technol Health Care. 2017;25(2):251-264. [doi: 10.3233/THC-161274] [Medline: 27886019]

67. Bergeron MF, Landset S, Tarpin-Bernard F, Ashford CB, Khoshgoftaar TM, Ashford JW. Episodic-memory performance in machine learning modeling for predicting cognitive health status classification. J Alzheimers Dis. 2019;70(1):277-286. [doi: 10.3233/JAD-190165] [Medline: 31177223]

68. Jia Z, Dong M, Shi Z, Jin C, Li G. Study of a screening system for mild cognitive impairment based on machine learning model. Journal of Shanghai Jiao Tong University (Medical Science). 2019;39(8):908-913. [doi: 10.3969/j.issn.1674- 8115.2019.08.017]

69. Rutkowski TM, Abe MS, Koculak M, Otake-Matsuura M. Classifying mild cognitive impairment from behavioral responses in emotional arousal and valence evaluation task - AI approach for early dementia biomarker in aging societies. Annu Int Conf IEEE Eng Med Biol Soc. Jul 2020;2020:5537-5543. [doi: 10.1109/EMBC44109.2020. 9175805] [Medline: 33019233]

70. Zhang Y, Chen Y, Yu H, et al. What can “drag & drop” tell? Detecting mild cognitive impairment by hand motor function assessment under dual-task paradigm. Int J Hum Comput Stud. Jan 2021;145:102547. [doi: 10.1016/j.ijhcs. 2020.102547]

71. Modarres MH, Kalafatis C, Apostolou P, Tabet N, Khaligh-Razavi SM. The use of the integrated cognitive assessment to improve the efficiency of primary care referrals to memory services in the accelerating dementia pathway technologies study. Front Aging Neurosci. 2023;15:1243316. [doi: 10.3389/fnagi.2023.1243316] [Medline: 37781102]

72. Leng F, Xu G, Long R, Xu W, Wang T, et al. Clinical application of an intelligently modified Mini-Cog assessment tool for screening cognitive impairment. Chinese Journal of Geriatric Heart Brain and Vessel Diseases. 2023;25(8):793-796. [doi: 10.3969/j.issn.1009-0126.2023.08.003]

73. Segev O, Raz I, Gerstein HC, et al. Development and first-stage validation of a digital version of the Digit Symbol Substitution test for use in assessing cognitive function in older people with diabetes. Diabetes Obes Metab. Aug 2024;26(8):3299-3305. [doi: 10.1111/dom.15657] [Medline: 38757537]

74. Huang G, Li R, Roccati E, et al. Feasibility of computerized motor, cognitive and speech tests in the home: analysis of TAS Test in 2,300 older adults. J Prev Alzheimers Dis. Apr 2025;12(4):100081. [doi: 10.1016/j.tjpad.2025.100081] [Medline: 40000322]

75. Liew TM, Foo JYH, Yang H, et al. PENSIEVE-AI a brief cognitive test to detect cognitive impairment across diverse literacy. Nat Commun. Mar 23, 2025;16(1):2847. [doi: 10.1038/s41467-025-58201-x] [Medline: 40122854]

76. Lusi A, Li T, Yu J, Zhang C. Automatic analysis pipeline of squatting ability for elderly people based on an intelligent platform. Annu Int Conf IEEE Eng Med Biol Soc. Jul 2024;2024:1-6. [doi: 10.1109/EMBC53108.2024.10781505] [Medline: 40039293]

77. Chung MK, Lee SY, Shin T, et al. BERT and BERTopic for screening clinical depression on open-ended text messages collected through a mobile application from older adults. BMC Public Health. Jun 10, 2025;25(1):2161. [doi: 10.1186/ s12889-025-23337-4] [Medline: 40495126]

78. Tang F, Uchendu I, Wang F, Dodge HH, Zhou J. Scalable diagnostic screening of mild cognitive impairment using AI dialogue agent. Sci Rep. Mar 31, 2020;10(1):5732. [doi: 10.1038/s41598-020-61994-0] [Medline: 32235884]

79. Fristed E, Skirrow C, Meszaros M, et al. A remote speech-based AI system to screen for early Alzheimer’s disease via smartphones. Alzheimers Dement (Amst). 2022;14(1):e12366. [doi: 10.1002/dad2.12366] [Medline: 36348974]

80. Lin D, Nazreen T, Rutowski T, et al. Feasibility of a machine learning-based smartphone application in detecting depression and anxiety in a generally senior population. Front Psychol. 2022;13(811517):811517. [doi: 10.3389/fpsyg. 2022.811517] [Medline: 35478769]

81. Kaywan P, Ahmed K, Ibaida A, Miao Y, Gu B. Early detection of depression using a conversational AI bot: a non- clinical trial. PLoS One. 2023;18(2):e0279743. [doi: 10.1371/journal.pone.0279743] [Medline: 36735701]

82. Kim D, Jang R, Jang SN, Park S. When a chatbot asks “How are you?”: A cross-sectional study of AI call conversations and depressive symptom detection among older adults in rural South Korea. BMC Geriatr. Jan 28, 2026;26(1):247. [doi: 10.1186/s12877-026-07038-0] [Medline: 41606503]

83. Haque RU, Pongos AL, Manzanares CM, Lah JJ, Levey AI, Clifford GD. Deep convolutional neural networks and transfer learning for measuring cognitive impairment using eye-tracking in a distributed tablet-based environment. IEEE Trans Biomed Eng. Jan 2021;68(1):11-18. [doi: 10.1109/TBME.2020.2990734] [Medline: 32340935]

84. Li Q, Yan J, Ye J, et al. Construction of a prediction model for Alzheimer’s disease using an AI-driven eye-tracking task on mobile devices. Aging Clin Exp Res. Dec 27, 2024;37(1):39725797. [doi: 10.1007/s40520-024-02882-9]

85. Vasan CS, Gupta S, Shekhar M, et al. Accuracy of an artificial intelligence-based mobile application for detecting cataracts: results from a field study. Indian J Ophthalmol (Poona City). 2023;71(8):2984-2989. [doi: 10.4103/IJO.IJO_3372_22]

86. Senthil S, Rao DP, Savoy FM, et al. Evaluating real-world performance of an automated offline glaucoma AI on a smartphone fundus camera across glaucoma severity stages. PLoS One. 2025;20(6):e0324883. [doi: 10.1371/journal. pone.0324883] [Medline: 40569941]

87. Jaworski D, Suwała K, Kaluzny BJ, Kaluzny JJ. Comparison of an AI-based mobile pupillometry system and NPi-200 for pupillary light reflex and correlation with glaucoma-related markers. Front Neurol. 2024;15:1426205. [doi: 10.3389/ fneur.2024.1426205] [Medline: 39850730]

88. Barbour DL, Howard RT, Song XD, et al. Online machine learning audiometry. Ear Hear. 2019;40(4):918-926. [doi: 10. 1097/AUD.0000000000000669] [Medline: 30358656]

89. Vercammen C, Strelcyk O. Development and validation of a self-administered online hearing test. Trends Hear. 2025;29:23312165251317923. [doi: 10.1177/23312165251317923] [Medline: 40101250]

90. Alves SA, Temme S, Motamedi S, et al. Evaluating the prognostic and clinical validity of the fall risk score derived from an AI-based mHealth app for fall prevention: retrospective real-world data analysis. JMIR Aging. Dec 4, 2024;7:e55681. [doi: 10.2196/55681] [Medline: 39631046]

91. Opoku Asare K, Terhorst Y, Vega J, Peltonen E, Lagerspetz E, Ferreira D. Predicting depression from smartphone behavioral markers using machine learning methods, hyperparameter optimization, and feature importance analysis: exploratory study. JMIR Mhealth Uhealth. Jul 12, 2021;9(7):e26540. [doi: 10.2196/26540] [Medline: 34255713]

92. Ferreira TJ, Salvador IC, Pessanha CR, et al. Advances in the estimation of body fat percentage using an artificial intelligence 2D-photo method. NPJ Digit Med. Jan 18, 2025;8(1):43. [doi: 10.1038/s41746-024-01380-6] [Medline: 39827323]

94. Obuchi SP, Kojima M, Suzuki H, et al. Artificial intelligence detection of cognitive impairment in older adults during walking. Alzheimers Dement (Amst). 2024;16(3):e70012. [doi: 10.1002/dad2.70012] [Medline: 39328904]

95. Mannini A, Trojaniello D, Cereatti A, Sabatini AM. A machine learning framework for gait classification using inertial sensors: application to elderly, post-stroke and Huntington’s disease patients. Sensors (Basel). Jan 21, 2016;16(1):26805847. [doi: 10.3390/s16010134] [Medline: 26805847]

96. Moon G, Cho J, Choi H, Kim Y, Kim GD, Jang SH. AI-based severity classification of dementia using gait analysis. Sensors (Basel). Oct 2, 2025;25(19):6083. [doi: 10.3390/s25196083] [Medline: 41094906]

97. Nguyen TQ, Young JH, Rodriguez A, Zupancic S, Lie DYC. Differentiation of patients with balance insufficiency (vestibular hypofunction) versus normal subjects using a low-cost small wireless wearable gait sensor. Biosensors (Basel). Feb 26, 2019;9(1):29. [doi: 10.3390/bios9010029] [Medline: 30813585]

98. Buisseret F, Catinus L, Grenard R, et al. Timed up and go and six-minute walking tests with wearable inertial sensor: one step further for the prediction of the risk of fall in elderly nursing home people. Sensors (Basel). Jun 5, 2020;20(11):3207. [doi: 10.3390/s20113207] [Medline: 32516995]

99. Jung D, Kim J, Mun KR. Identifying depression in the elderly using gait accelerometry. Annu Int Conf IEEE Eng Med Biol Soc. Jul 2022;2022(4946-9):4946-4949. [doi: 10.1109/EMBC48229.2022.9871877] [Medline: 36086152]

100. Seifer AK, Kuderle A, Dorschky E, Moradi H, Hannemann R, Eskofier BM. Step length and gait speed estimation using a hearing aid integrated accelerometer: a comparison of different algorithms. IEEE J Biomed Health Inform. Nov 2024;28(11):6619-6628. [doi: 10.1109/JBHI.2024.3454824] [Medline: 39236137]

101. Wipperman MF, Lin AZ, Gayvert KM, et al. Digital wearable insole-based identification of knee arthropathies and gait signatures using machine learning. Elife. Apr 30, 2024;13:e86132. [doi: 10.7554/eLife.86132] [Medline: 38686919]

102. Yan F, Gong J, Zhang Q, He H. Learning motion primitives for the quantification and diagnosis of mobility deficits. IEEE Trans Biomed Eng. Dec 2024;71(12):3339-3349. [doi: 10.1109/TBME.2024.3404357] [Medline: 38776195]

103. Cai J, Guan Z, Wang J, et al. Impact of gait parameters and their variability on fall risk assessment accuracy using wearable sensor. IEEE Trans Neural Syst Rehabil Eng. 2025;33(1996-2003):1996-2003. [doi: 10.1109/TNSRE.2025. 3572109] [Medline: 40397645]

104. Ghosh S, Fleiner T, Giannouli E, et al. Statistical learning of mobility patterns from long-term monitoring of locomotor behaviour with body-worn sensors. Sci Rep. May 4, 2018;8(1):7079. [doi: 10.1038/s41598-018-25523-4] [Medline: 29728658]

105. Gramkow MH, Brink-Kjær A, Clemmensen FK, et al. Diagnostic performance of actigraphy in Alzheimer’s disease using a machine learning classifier – a cross-sectional memory clinic study. Alz Res Therapy. May 21, 2025;17(1):40399918. [doi: 10.1186/s13195-025-01751-5]

106. Jacobson NC, Weingarden H, Wilhelm S. Digital biomarkers of mood disorders and symptom change. NPJ Digit Med. 2019;2(3):3. [doi: 10.1038/s41746-019-0078-0] [Medline: 31304353]

107. Rykov Y, Thach TQ, Bojic I, Christopoulos G, Car J. Digital biomarkers for depression screening with wearable devices: cross-sectional study with machine learning modeling. JMIR Mhealth Uhealth. Oct 25, 2021;9(10):e24872. [doi: 10.2196/24872] [Medline: 34694233]

108. Yang D, Hong KS. Quantitative assessment of resting-state for mild cognitive impairment detection: a functional near- infrared spectroscopy and deep learning approach. J Alzheimers Dis. 2021;80(2):647-663. [doi: 10.3233/JAD-201163] [Medline: 33579839]

109. Ho TKK, Kim M, Jeon Y, et al. Deep learning-based multilevel classification of Alzheimer’s disease using non-invasive functional near-infrared spectroscopy. Front Aging Neurosci. 2022;14:810125. [doi: 10.3389/fnagi.2022.810125] [Medline: 35557842]

110. Pu Z, Huang H, Li M, et al. Screening tools for subjective cognitive decline and mild cognitive impairment based on task-state prefrontal functional connectivity: a functional near-infrared spectroscopy study. Neuroimage. Apr 15, 2025;310:121130. [doi: 10.1016/j.neuroimage.2025.121130] [Medline: 40058532]

111. Choi J, Lee S, Kim S, Kim D, Kim H. Depressed mood prediction of elderly people with a wearable band. Sensors. May 31, 2022;22(11):4174. [doi: 10.3390/s22114174]

112. Lee TR, Kim GH, Choi MT. Geriatric depression and anxiety screening via deep learning using activity tracking and sleep data. Int J Geriatr Psychiatry. Feb 2024;39(2):e6071. [doi: 10.1002/gps.6071] [Medline: 38372966]

113. Rykov YG, Ng KP, Patterson MD, Gangwar BA, Kandiah N. Predicting the severity of mood and neuropsychiatric symptoms from digital biomarkers using wearable physiological data and deep learning. Comput Biol Med. Sep 2024;180:108959. [doi: 10.1016/j.compbiomed.2024.108959] [Medline: 39089109]

114. Han S, Xiao Q, Liang Y, et al. Using flexible-printed piezoelectric sensor arrays to measure plantar pressure during walking for sarcopenia screening. Sensors (Basel). Aug 11, 2024;24(16):5189. [doi: 10.3390/s24165189] [Medline: 39204885]

115. Lai DTH, Begg RK, Taylor S, Palaniswami M. Detection of tripping gait patterns in the elderly using autoregressive features and support vector machines. J Biomech. 2008;41(8):1762-1772. [doi: 10.1016/j.jbiomech.2008.02.037] [Medline: 18433757]

116. Ballesteros J, Urdiales C, Martinez AB, Tirado M. Automatic assessment of a rollator-user’s condition during rehabilitation using the i-Walker platform. IEEE Trans Neural Syst Rehabil Eng. Nov 2017;25(11):2009-2017. [doi: 10. 1109/TNSRE.2017.2698005] [Medline: 28459694]

117. Wu LW, OuYoung T, Chiu YC, Hsieh HF, Hsiu H. Discrimination between possible sarcopenia and metabolic syndrome using the arterial pulse spectrum and machine-learning analysis. Sci Rep. Dec 12, 2022;12(1):21452. [doi: 10. 1038/s41598-022-26074-5] [Medline: 36509825]

118. Dawadi PN, Cook DJ, Schmitter-Edgecombe M. Automated cognitive health assessment using smart home monitoring of complex tasks. IEEE Trans Syst Man Cybern Syst. Nov 2013;43(6):1302-1313. [doi: 10.1109/TSMC.2013.2252338] [Medline: 25530925]

119. König A, Crispim-Junior CF, Covella AGU, et al. Ecological assessment of autonomy in instrumental activities of daily living in dementia patients by the means of an automatic video monitoring system. Front Aging Neurosci. 2015;7(JUN):98. [doi: 10.3389/fnagi.2015.00098] [Medline: 26082715]

120. Alberdi A, Weakley A, Schmitter-Edgecombe M, et al. Smart home-based prediction of multidomain symptoms related to Alzheimer’s disease. IEEE J Biomed Health Inform. Nov 2018;22(6):1720-1731. [doi: 10.1109/JBHI.2018.2798062] [Medline: 29994359]

121. Zolfaghari S, Khodabandehloo E, Riboni D. TraMiner: vision-based analysis of locomotion traces for cognitive assessment in smart-homes. Cognit Comput. 2022;14(5):1549-1570. [doi: 10.1007/s12559-020-09816-3] [Medline: 33552305]

122. Dawadi PN, Cook DJ, Schmitter-Edgecombe M. Automated cognitive health assessment from smart home-based behavior data. IEEE J Biomed Health Inform. Jul 2016;20(4):1188-1194. [doi: 10.1109/JBHI.2015.2445754] [Medline: 26292348]

123. Alsubai S, Alqahtani A, Sha M, Abbas S, Gregus M, Furda R. Automated cognitive health assessment based on daily life functional activities. Comput Intell Neurosci. 2023;2023:5684914. [doi: 10.1155/2023/5684914] [Medline: 37455767]

124. Méndez JI, Meza-Sánchez AV, Ponce P, et al. Smart homes as enablers for depression pre-diagnosis using PHQ-9 on HMI through fuzzy logic decision system. Sensors (Basel). Nov 26, 2021;21(23):7864. [doi: 10.3390/s21237864] [Medline: 34883868]

125. Faruk T, Shum LC, Iaboni A, Khan SS. Walking path images from real-time location data predict degree of cognitive impairment. Artif Intell Med. Oct 2023;144:102657. [doi: 10.1016/j.artmed.2023.102657] [Medline: 37783548]

126. Khosroazad S, Abedi A, Hayes MJ. Sleep signal analysis for early detection of Alzheimer’s disease and related dementia (ADRD). IEEE J Biomed Health Inform. May 2023;27(5):2264-2275. [doi: 10.1109/JBHI.2023.3235391] [Medline: 37018587]

127. Toth L, Hoffmann I, Gosztolya G, et al. A speech recognition-based solution for the automatic detection of mild cognitive impairment from spontaneous speech. Curr Alzheimer Res. 2018;15(2):130-138. [doi: 10.2174/ 1567205014666171121114930] [Medline: 29165085]

128. Thomas JA, Burkhardt HA, Chaudhry S, et al. Assessing the utility of language and voice biomarkers to predict cognitive impairment in the Framingham heart study cognitive aging cohort data. J Alzheimers Dis. 2020;76(3):905-922. [doi: 10.3233/JAD-190783] [Medline: 32568190]

129. Badal VD, Nebeker C, Shinkawa K, et al. Do words matter? Detecting social isolation and loneliness in older adults using natural language processing. Front Psychiatry. 2021;12:728732. [doi: 10.3389/fpsyt.2021.728732] [Medline: 34867518]

130. Diaz-Asper C, Chandler C, Turner RS, Reynolds B, Elvevåg B. Increasing access to cognitive screening in the elderly: applying natural language processing methods to speech collected over the telephone. Cortex. Nov 2022;156:26-38. [doi: 10.1016/j.cortex.2022.08.005] [Medline: 36179481]

131. Amini S, Hao B, Zhang L, et al. Automated detection of mild cognitive impairment and dementia from voice recordings: a natural language processing approach. Alzheimers Dement. Mar 2023;19(3):946-955. URL: https://alz-journals. onlinelibrary.wiley.com/toc/15525279/19/3 [Accessed 2026-08-21] [doi: 10.1002/alz.12721]

132. Pourramezan Fard A, Mahoor MH, Alsuhaibani M, Dodge HH. Linguistic-based mild cognitive impairment detection using informative loss. Comput Biol Med. Jun 2024;176:108606. [doi: 10.1016/j.compbiomed.2024.108606] [Medline: 38763068]

133. Zhang Z, Wang T, Hu Z, Yang LZ, Li H. DEMENTIA: a hybrid attention-based multimodal and multi-task learning framework with expert knowledge for Alzheimer’s disease assessment from speech. IEEE J Biomed Health Inform. ;29(4):2957-2968. [doi: 10.1109/JBHI.2024.3509620]

134. Peng M, Zhang Y, Fan Y, Zhang M, Ishimaru M, Li C, et al. Rapid detection of mild cognitive impairment using natural language processing. Chinese Journal of Physical Medicine and Rehabilitation. 2023;45(7):592-597. [doi: 10.3760/cma. j.issn.0254-1424.2023.07.003]

135. Ambrosini E, Giangregorio C, Lomurno E, et al. Automatic spontaneous speech analysis for the detection of cognitive functional decline in older adults: multilanguage cross-sectional study. JMIR Aging. Apr 29, 2024;7:e50537. [doi: 10. 2196/50537] [Medline: 38386279]

136. Smith M, Dietrich BJ, Bai EW, Bockholt HJ. Vocal pattern detection of depression among older adults. Int J Ment Health Nurs. Jun 2020;29(3):440-449. [doi: 10.1111/inm.12678] [Medline: 31811697]

137. Mazur A, Costantino H, Tom P, Wilson MP, Thompson RG. Evaluation of an AI-based voice biomarker tool to detect signals consistent with moderate to severe depression. Ann Fam Med. Jan 27, 2025;23(1):60-65. [doi: 10.1370/afm. 240091] [Medline: 39805690]

138. Song XD, Wallace BM, Gardner JR, Ledbetter NM, Weinberger KQ, Barbour DL. Fast, continuous audiogram estimation using machine learning. Ear Hear. 2015;36(6):e326-35. [doi: 10.1097/AUD.0000000000000186] [Medline: 26258575]

139. Heisey KL, Walker AM, Xie K, Abrams JM, Barbour DL. Dynamically masked audiograms with machine learning audiometry. Ear Hear. 2020;41(6):1692-1702. [doi: 10.1097/AUD.0000000000000891] [Medline: 33136643]

140. Zhang M, Bi Z, Fu X, et al. A parsimonious approach for screening moderate-to-profound hearing loss in a community- dwelling geriatric population based on a decision tree analysis. BMC Geriatr. Aug 7, 2019;19(1):214. [doi: 10.1186/ s12877-019-1232-x] [Medline: 31390985]

141. Charih F, Bromwich M, Mark AE, Lefrançois R, Green JR. Data-driven audiogram classification for mobile audiometry. Sci Rep. Mar 3, 2020;10(1):3962. [doi: 10.1038/s41598-020-60898-3] [Medline: 32127604]

142. Kassjański M, Kulawiak M, Przewoźny T, et al. Automated hearing loss type classification based on pure tone audiometry data. Sci Rep. Jun 20, 2024;14(1):14203. [doi: 10.1038/s41598-024-64310-2] [Medline: 38902305]

143. Wallaert N, Perry A, Jean H, Creff G, Godey B, Paraouty N. Performance and reliability evaluation of an automated bone-conduction audiometry using machine learning. Trends Hear. 2024;28:23312165241286456. [doi: 10.1177/ 23312165241286456] [Medline: 39489159]

144. Souillard-Mandar W, Penney D, Schaible B, Pascual-Leone A, Au R, Davis R. DCTclock: clinically-interpretable and automated artificial intelligence analysis of drawing behavior for capturing cognition. Front Digit Health. 2021;3:750661. [doi: 10.3389/fdgth.2021.750661] [Medline: 34723243]

145. Kobayashi M, Yamada Y, Shinkawa K, Nemoto M, Nemoto K, Arai T. Automated early detection of Alzheimer’s disease by capturing impairments in multiple cognitive domains with multiple drawing tasks. J Alzheimers Dis. 2022;88(3):1075-1089. [doi: 10.3233/JAD-215714] [Medline: 35723100]

146. Ruengchaijatuporn N, Chatnuntawech I, Teerapittayanon S, et al. An explainable self-attention deep neural network for detecting mild cognitive impairment using multi-input digital drawing tasks. Alz Res Therapy. Aug 9, 2022;14(1):35945568. [doi: 10.1186/s13195-022-01043-2]

147. Kawa J, Bednorz A, Stępień P, Derejczyk J, Bugdol M. Spatial and dynamical handwriting analysis in mild cognitive impairment. Comput Biol Med. Mar 1, 2017;82(21-8):21-28. [doi: 10.1016/j.compbiomed.2017.01.004] [Medline: 28126631]

148. Kang L, Zhang X, Guan J, Huang K, Wu R. Early Alzheimer’s disease diagnosis via handwriting with self-attention mechanisms. J Alzheimers Dis. Nov 2024;102(1):173-180. [doi: 10.1177/13872877241283920] [Medline: 39497308]

149. Yoshii K, Nishimura M, Kimura D, et al. A study for detecting mild cognitive impairment by analyzing conversations with humanoid robots. Presented at: 2021 IEEE 3rd Global Conference on Life Sciences and Technologies (LIFETECH); 347-350; Nara, Japan. March 9-11, 2021.URL: https://ieeexplore.ieee.org/xpl/mostRecentIssue.jsp? punumber=9391771 [Accessed 2026-08-21] [doi: 10.1109/LifeTech52111.2021.9391934]

150. Chang YL, Luo DH, Huang TR, Goh JOS, Yeh SL, Fu LC. Identifying mild cognitive impairment by using human-robot interactions. J Alzheimers Dis. 2022;85(3):1129-1142. [doi: 10.3233/JAD-215015] [Medline: 34897086]

151. Nishiura Y, Inoue T, Takaeda K, Kamimura T. Inter- and intra-rater reliability of cognitive assessment conducted by assistive robot for older adults living in the community: a preliminary study. Psychogeriatrics. Jan 2025;25(1):e13203. [doi: 10.1111/psyg.13203] [Medline: 39444248]

152. Tsai CF, Chen CC, Wu EHK, et al. A machine-learning-based assessment method for early-stage neurocognitive impairment by an immersive virtual supermarket. IEEE Trans Neural Syst Rehabil Eng. 2021;29:2124-2132. [doi: 10. 1109/TNSRE.2021.3118918] [Medline: 34623270]

153. Kim SY, Park J, Choi H, Loeser M, Ryu H, Seo K. Digital marker for early screening of mild cognitive impairment through hand and eye movement analysis in virtual reality using machine learning: first validation study. J Med Internet Res. Oct 20, 2023;25:e48093. [doi: 10.2196/48093] [Medline: 37862101]

154. Wang Y, Yang P, Yu J, et al. An ensemble learning algorithm for cognitive evaluation by an immersive virtual reality supermarket. IEEE Trans Neural Syst Rehabil Eng. 2024;32:3761-3772. [doi: 10.1109/TNSRE.2024.3470802]

155. Fang L, Fan X, Li A, et al. Detecting Alzheimer’s disease using digital virtual reality cognitive tests. Alzheimers Dement. Dec 2025;21(12):41451798. URL: https://alz-journals.onlinelibrary.wiley.com/toc/15525279/21/12 [Accessed 2026-08-21] [doi: 10.1002/alz.71039]

156. Fraser KC, Lundholm Fors K, Eckerström M, Öhman F, Kokkinakis D. Predicting MCI status from multimodal language data using cascaded classifiers. Front Aging Neurosci. 2019;11(205):205. [doi: 10.3389/fnagi.2019.00205] [Medline: 31427959]

157. Shah HA, Andberg S, Koivisto AM, Bednarik R. A multimodal approach for early identification of mild cognitive impairment and Alzheimer’s disease with fusion network using eye movements and speech. IEEE Trans Neural Syst Rehabil Eng. 2025;33:1449-1459. [doi: 10.1109/TNSRE.2025.3561043] [Medline: 40232896]

158. Poor FF, Dodge HH, Mahoor MH. A multimodal cross-transformer-based model to predict mild cognitive impairment using speech, language and vision. Comput Biol Med. Nov 2024;182:109199. [doi: 10.1016/j.compbiomed.2024. 109199] [Medline: 39332117]

159. Zhang X, Zhang F, Hou S, et al. Effectiveness of digital screening tools in detecting cognitive impairment among community-dwelling elderly in Northern China: a large cohort study. J Prev Alzheimers Dis. Mar 2025;12(3):100080. [doi: 10.1016/j.tjpad.2025.100080] [Medline: 39922758]

160. Lin J, Xu T, Yang X, et al. A detection model of cognitive impairment via the integrated gait and eye movement analysis from a large Chinese community cohort. Alzheimer’s & Dementia. Feb 2024;20(2):1089-1101. [doi: 10.1002/alz.13517]

161. Guimarães V, Sousa I, Cunha R, et al. One-class classification with confound control for cognitive screening in older adults using gait, fingertapping, cognitive, and dual tasks. Comput Methods Programs Biomed. Feb 2025;259:108508. [doi: 10.1016/j.cmpb.2024.108508]

162. Lentzen M, Vairavan S, Muurling M, et al. RADAR-AD: assessment of multiple remote monitoring technologies for early detection of Alzheimer’s disease. Alz Res Therapy. Jan 27, 2025;17(1):39865315. [doi: 10.1186/s13195-025- 01675-0]

163. Khosroazad S, Gilbert CF, Aronis JB, et al. Sleep movements and respiratory coupling as a biobehavioral metric for early Alzheimer’s disease in independently dwelling adults. BMC Geriatr. Apr 27, 2023;23(1):252. [doi: 10.1186/ s12877-023-03983-2] [Medline: 37106470]

164. Butler PM, Yang J, Brown R, et al. Smartwatch- and smartphone-based remote assessment of brain health and detection of mild cognitive impairment. Nat Med. Mar 2025;31(3):829-839. [doi: 10.1038/s41591-024-03475-9] [Medline: 40038507]

165. Park I, Lee SK, Choi HC, et al. Machine learning-based estimation of the mild cognitive impairment stage using multimodal physical and behavioral measures. Sci Rep. Oct 9, 2025;15(1):35369. [doi: 10.1038/s41598-025-19364-1] [Medline: 41068341]

166. Ming A, Clemens V, Lorek E, et al. Game-based assessment of peripheral neuropathy combining sensor-equipped insoles, video games, and AI: proof-of-concept study. J Med Internet Res. Oct 1, 2024;26:e52323. [doi: 10.2196/52323] [Medline: 39353184]

167. Ming A, Schubert T, Marr V, Hötzsch J, Stober S, Mertens PR. Video game-based application for fall risk assessment: a proof-of-concept cohort study. EClinicalMedicine. Dec 2024;78:102947. [doi: 10.1016/j.eclinm.2024.102947] [Medline: 39677357]

168. Howcroft J, Kofman J, Lemaire ED. Prospective fall-risk prediction models for older adults based on wearable sensors. IEEE Trans Neural Syst Rehabil Eng. Oct 2017;25(10):1812-1820. [doi: 10.1109/TNSRE.2017.2687100] [Medline: 28358689]

169. Lincke A, Fagerström C, Ekstedt M, Löwe W, Backåberg S. A comparative study of the 2D- and 3D-based skeleton avatar technology for assessing physical activity and functioning among healthy older adults. Health Informatics J. 2023;29(4):14604582231214589. [doi: 10.1177/14604582231214589] [Medline: 37924210]

170. Kong HH, Shin K, Yang DS, Gu HY, Joo HS, Shon HC. Digital assessment of walking ability: Validity and reliability of the automated figure-of-eight walk test in older adults. PLoS One. 2025;20(2):e0316612. [doi: 10.1371/journal.pone. 0316612] [Medline: 39928640]

171. Kim S, Kim HS, Yoo JI. Sarcopenia classification model for musculoskeletal patients using smart insole and artificial intelligence gait analysis. J Cachexia Sarcopenia Muscle. Dec 2023;14(6):2793-2803. [doi: 10.1002/jcsm.13356] [Medline: 37884824]

172. Naseem MT, Kim NH, Seo H, et al. Sarcopenia diagnosis using skeleton-based gait sequence and foot-pressure image datasets. Front Public Health. 2024;12(1443188):1443188. [doi: 10.3389/fpubh.2024.1443188] [Medline: 39664552]

173. Wang X, Wang W, Chen M, et al. Using 3D facial information to predict malnutrition and consequent complications. Nutr Clin Pract. Dec 2024;39(6):1354-1363. [doi: 10.1002/ncp.11215] [Medline: 39319394]

174. Di Martino F, Delmastro F, Dolciotti C. Explainable AI for malnutrition risk prediction from m-Health and clinical data. Smart Health. Dec 2023;30:100429. [doi: 10.1016/j.smhl.2023.100429]

175. Armadá-Maresca F, Capote-Díaz M, Cidad-Betegón MDP, et al. Clinical performance of an interactive platform based on artificial intelligence in ophthalmology: experience in a third-level reference center. Front Med (Lausanne). 2025;12:1593556. [doi: 10.3389/fmed.2025.1593556] [Medline: 40837569]

176. Nickels S, Edwards MD, Poole SF, et al. Toward a mobile platform for real-world digital measurement of depression: user-centered design, data quality, and behavioral and clinical modeling. JMIR Ment Health. Aug 10, 2021;8(8):e27589. [doi: 10.2196/27589] [Medline: 34383685]

177. Kim H, Lee S, Lee S, Hong S, Kang H, Kim N. Depression prediction by using ecological momentary assessment, actiwatch data, and machine learning: observational study on older adults living alone. JMIR Mhealth Uhealth. Oct 16, 2019;7(10):e14149. [doi: 10.2196/14149] [Medline: 31621642]

178. Minaeva O, Riese H, Lamers F, Antypa N, Wichers M, Booij SH. Screening for depression in daily life: development and external validation of a prediction model based on actigraphy and experience sampling method. J Med Internet Res. Dec 1, 2020;22(12):e22634. [doi: 10.2196/22634] [Medline: 33258783]

179. Pedrelli P, Fedor S, Ghandeharioun A, et al. Monitoring changes in depression severity using wearable and mobile sensors. Front Psychiatry. 2020;11:584711. [doi: 10.3389/fpsyt.2020.584711] [Medline: 33391050]

180. Opoku Asare K, Moshe I, Terhorst Y, et al. Mood ratings and digital biomarkers from smartphone and wearable data differentiates and predicts depression status: a longitudinal data analysis. Pervasive Mob Comput. Jul 2022;83:101621. [doi: 10.1016/j.pmcj.2022.101621]

181. Song S, Seo Y, Hwang S, Kim HY, Kim J. Digital phenotyping of geriatric depression using a community-based digital mental health monitoring platform for socially vulnerable older adults and their community caregivers: 6-week living lab single-arm pilot study. JMIR Mhealth Uhealth. Jun 17, 2024;12:e55842. [doi: 10.2196/55842] [Medline: 38885033]

182. Zhang Y, Stewart C, Ranjan Y, et al. Large-scale digital phenotyping: Identifying depression and anxiety indicators in a general UK population with over 10,000 participants. J Affect Disord. Apr 2025;375:412-422. [doi: 10.1016/j.jad.2025. 01.124]

183. Little B, Alshabrawy O, Stow D, et al. Deep learning-based automated speech detection as a marker of social functioning in late-life depression. Psychol Med. Jul 2021;51(9):1441-1450. [doi: 10.1017/S0033291719003994] [Medline: 31944174]

184. Zhou Y, Han W, Yao X, Xue J, Li Z, Li Y. Developing a machine learning model for detecting depression, anxiety, and apathy in older adults with mild cognitive impairment using speech and facial expressions: a cross-sectional observational study. Int J Nurs Stud. Oct 2023;146:104562. [doi: 10.1016/j.ijnurstu.2023.104562] [Medline: 37531702]

185. Liu Y, Li X, Wang M, et al. Multimodal depression recognition and analysis: facial expression and body posture changes via emotional stimuli. J Affect Disord. Jul 2025;381(44-54):44-54. [doi: 10.1016/j.jad.2025.03.155]

186. Fan L, Wang FY, Zhao JH, et al. From physical activity patterns to cognitive status: development and validation of novel digital biomarkers for cognitive assessment in older adults. Int J Behav Nutr Phys Act. 2025;22:11. [doi: 10.1186/s12966-025-01706-x]
