# Supplementary material for: AI-Based Measurement Tools for Intrinsic Capacity in Older Adults: Scoping Review
Source: J Med Internet Res. 2026 Oct 5;28:e90411. doi: 10.2196/90411 (PMC13639135; doi:10.2196/90411)
Supplement: Supplementary file 4 [file jmir-v28-e90411-s002.doc]

Multimedia Appendix 2: supplementary figures

- Supplementary figure 1. Number of studies by published year.
- Supplementary figure 2. Number of studies by AI-based measurement tools for IC domain.
- Supplementary figure 3. Number of studies by AI techniques categories.
- Supplementary figure 4. Number of studies by AI techniques combination.

Supplementary figure 1. Number of studies by published year.

Supplementary figure 2. Number of studies by AI-based measurement tools for IC domain.

Supplementary figure 3. Number of studies by AI techniques categories.

Supplementary figure 4. Number of studies by AI techniques combination.
