# Supplementary material for: AI-Based Measurement Tools for Intrinsic Capacity in Older Adults: Scoping Review
Source: J Med Internet Res. 2026 Oct 5;28:e90411. doi: 10.2196/90411 (PMC13639135; doi:10.2196/90411)
Supplement: Supplementary file 5 [file jmir-v28-e90411-s005.doc]

- Glossary

1. **Older Adults**: In this article, older adults are defined as individuals aged ≥60 years.
2. **Intrinsic Capacity (IC)**: Intrinsic Capacity is a composite measure of an individual’s physical and mental capacities, reflecting the overall potential for healthy functioning in older age. It is a dynamic concept proposed by the World Health Organization and typically encompasses multiple domains, such as cognitive capacity, locomotor capacity, psychological capacity, sensory capacity, and vitality. IC emphasizes a person’s underlying reserves and functional abilities rather than the presence or absence of specific diseases.
3. **AI-based IC measurement tools**: This scoping review defines AI-based IC measurement tools as integrated systems that combine AI techniques with digital devices to assess one or more domains of IC through digital biomarkers.
4. **Multimodal data acquisition devices (MDAD)**: Multimodal data refers to a collection of information derived from different perceptual channels or data modalities. Therefore, multimodal data acquisition devices (MDAD) are formed by combining different types of AI-based IC measurement tools.
5. **Computer vision system**: A computer vision system refers to a system that uses computer vision (CV) technology to interpret and analyse visual data (images or videos), and these systems can measure vision, cognition, locomotion, psychological capacity, and vitality
6. **AI-driven health platform (AI platform)**: AI-driven health platforms (hereinafter “AI platforms”), combining computing devices and software, including smartphone and tablet applications, computer programs, online platforms, and chatbot have seen wide application in health promotion and disease prevention among older adults.
7. **Wearable device**: A wearable device is a body-worn sensor for data collection that has become a mature and cost-effective technology widely used in elderly care
8. **Smart home**: A smart home is a residential environment built upon connected sensors and automation technologies, in which AI serves as the core driver that transforms it from simple automation to intelligent adaptation
9. **Speech analysis system**: A speech analysis system typically comprises three stages: (1) speech acquisition, (2) extraction of acoustic or linguistic features (the latter via ASR or transcription), and (3) modeling and analysis with AI methods
10. **AI audiometer**: An AI audiometer is an audiometric assessment system that integrates artificial intelligence techniques to support or automate hearing evaluation. It typically uses algorithms to conduct adaptive hearing tests, analyze auditory responses, and interpret audiometric data (e.g., audiograms), thereby improving the efficiency, accuracy, and scalability of hearing capacity assessment, including in remote or unsupervised settings.
11. **Virtual reality system**: Virtual reality (VR) systems are computer-based technologies that create immersive, interactive three-dimensional environments in which users can experience and interact with simulated scenes through head-mounted displays, motion sensors, and other input devices.
12. **Fixed sensor device**: In this review, we define fixed sensor devices as non-wearable, contact-based measurement systems installed in fixed spatial positions that acquire biomechanical or physiological signals through direct human–device interaction
13. **Digital biomarkers (DBs)**: Digital biomarkers are objective, quantifiable physiological and behavioral data that are collected and measured using digital devices such as smartphones, wearables, sensors, or other connected technologies.
14. **Machine Learning (ML)**: Machine learning is a subfield of artificial intelligence that enables computer systems to learn patterns from data and improve their performance on specific tasks without being explicitly programmed. It encompasses a variety of algorithms that map inputs to outputs or discover structure in data. Machine learning encompasses supervised learning (SL), unsupervised learning (UL), deep learning (DL), and reinforcement learning (RL).
15. **Computer Vision (CV)**: Computer vision is a field of artificial intelligence that focuses on enabling machines to interpret, analyze, and understand visual information from images or videos. Typical tasks include object detection, image classification, tracking, and scene understanding.
16. **Natural Language Processing (NLP)**: Natural language processing is a branch of artificial intelligence that deals with the interaction between computers and human language. It aims to enable machines to understand, generate, and analyze text or speech, supporting tasks such as classification, translation, and dialogue.
17. **Robotics and chatbots**: Robotics refers to the design, construction, and use of robots that can sense, process information, and act in the physical world to perform tasks autonomously or semi-autonomously. Chatbots are conversational agents that use natural language interfaces to interact with users via text or speech, often providing information, guidance, or support.
18. **Knowledge Representation and Reasoning (KRR)**: Knowledge representation and reasoning is a subfield of artificial intelligence concerned with how to formally encode knowledge about the world and how to draw logical inferences from it. It supports tasks such as decision-making, problem solving, and explanation by using structured representations (e.g., ontologies, rules, or graphs).
19. **Supervised Learning (SL)**: Supervised learning is a machine learning paradigm in which models are trained on labeled data, where each input is paired with a known output. The goal is to learn a mapping that can accurately predict outputs for new, unseen inputs.
20. **Unsupervised Learning (UL)**: Unsupervised learning is a machine learning paradigm that analyzes unlabeled data to discover hidden patterns, structures, or groupings. Common tasks include clustering, dimensionality reduction, and anomaly detection.
21. **Deep Learning (DL)**: Deep learning is a subset of machine learning that uses neural networks with multiple layers to automatically learn hierarchical feature representations from large amounts of data. It has achieved state-of-the-art performance in areas such as computer vision, speech recognition, and natural language processing.
22. **Reinforcement Learning (RL)**: Reinforcement learning is a learning paradigm in which an agent interacts with an environment by taking actions and receiving rewards or penalties. The agent learns a policy that maximizes cumulative reward over time through trial and error.
